# Birthweight is associated with clinical characteristics in people with recently diagnosed type 2 diabetes

**DOI:** 10.1007/s00125-023-05936-1

**Published:** 2023-06-12

**Authors:** Aleksander L. Hansen, Reimar W. Thomsen, Charlotte Brøns, Helene M. L. Svane, Rasmus T. Jensen, Mette K. Andersen, Torben Hansen, Jens S. Nielsen, Peter Vestergaard, Kurt Højlund, Niels Jessen, Michael H. Olsen, Henrik T. Sørensen, Allan A. Vaag

**Affiliations:** 1 Steno Diabetes Center Copenhagen, Herlev, Denmark; 2 Department of Clinical Epidemiology, Aarhus University and Aarhus University Hospital, Aarhus, Denmark; 3 Novo Nordisk Foundation Center for Basic Metabolic Research, University of Copenhagen, Copenhagen, Denmark; 4 Steno Diabetes Center Odense, Odense University Hospital, Odense, Denmark; 5 Steno Diabetes Center North Denmark, Aalborg University Hospital, Aalborg, Denmark; 6 Steno Diabetes Center Aarhus University Hospital, Aarhus, Denmark; 7 Department of Regional Health Research, University of Southern Denmark, Odense, Denmark; 8 Department of Internal Medicine and Steno Diabetes Center Zealand, Holbæk Hospital, Holbæk, Denmark; 9 Lund University Diabetes Center, Lund University, Malmö, Sweden; 10 Department of Endocrinology, Skåne University Hospital, Malmö, Sweden

**Keywords:** Age at diagnosis, Birthweight, Epidemiology, Fetal programming, Polygenic risk score, Type 2 diabetes

## Abstract

**Aims/hypothesis:**

Low birthweight is a risk factor for type 2 diabetes but it is unknown whether low birthweight is associated with distinct clinical characteristics at disease onset. We examined whether a lower or higher birthweight in type 2 diabetes is associated with clinically relevant characteristics at disease onset.

**Methods:**

Midwife records were traced for 6866 individuals with type 2 diabetes in the Danish Centre for Strategic Research in Type 2 Diabetes (DD2) cohort. Using a cross-sectional design, we assessed age at diagnosis, anthropomorphic measures, comorbidities, medications, metabolic variables and family history of type 2 diabetes in individuals with the lowest 25% of birthweight (<3000 g) and highest 25% of birthweight (>3700 g), compared with a birthweight of 3000–3700 g as reference, using log-binomial and Poisson regression. Continuous relationships across the entire birthweight spectrum were assessed with linear and restricted cubic spline regression. Weighted polygenic scores (PS) for type 2 diabetes and birthweight were calculated to assess the impact of genetic predispositions.

**Results:**

Each 1000 g decrease in birthweight was associated with a 3.3 year (95% CI 2.9, 3.8) younger age of diabetes onset, 1.5 kg/m^2^ (95% CI 1.2, 1.7) lower BMI and 3.9 cm (95% CI 3.3, 4.5) smaller waist circumference. Compared with the reference birthweight, a birthweight of <3000 g was associated with more overall comorbidity (prevalence ratio [PR] for Charlson Comorbidity Index Score ≥3 was 1.36 [95% CI 1.07, 1.73]), having a systolic BP ≥155 mmHg (PR 1.26 [95% CI 0.99, 1.59]), lower prevalence of diabetes-associated neurological disease, less likelihood of family history of type 2 diabetes, use of three or more glucose-lowering drugs (PR 1.33 [95% CI 1.06, 1.65]) and use of three or more antihypertensive drugs (PR 1.09 [95% CI 0.99, 1.20]). Clinically defined low birthweight (<2500 g) yielded stronger associations. Most associations between birthweight and clinical characteristics appeared linear, and a higher birthweight was associated with characteristics mirroring lower birthweight in opposite directions. Results were robust to adjustments for PS representing weighted genetic predisposition for type 2 diabetes and birthweight.

**Conclusion/interpretation:**

Despite younger age at diagnosis, and fewer individuals with obesity and family history of type 2 diabetes, a birthweight <3000 g was associated with more comorbidities, including a higher systolic BP, as well as with greater use of glucose-lowering and antihypertensive medications, in individuals with recently diagnosed type 2 diabetes.

**Graphical Abstract:**

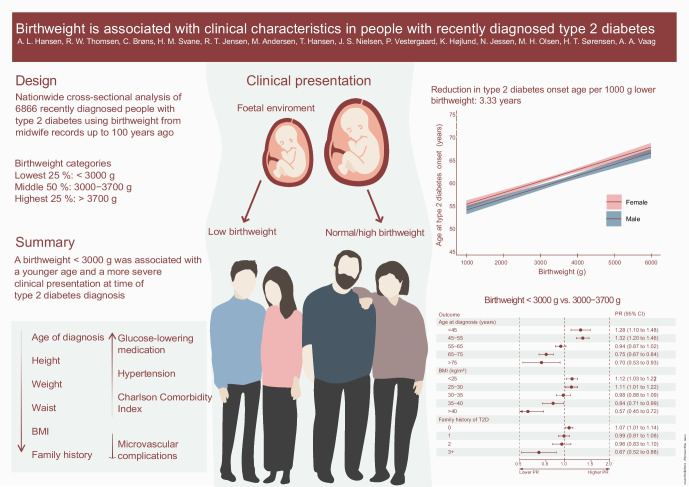

**Supplementary Information:**

The online version contains peer-reviewed but unedited supplementary material available at 10.1007/s00125-023-05936-1.


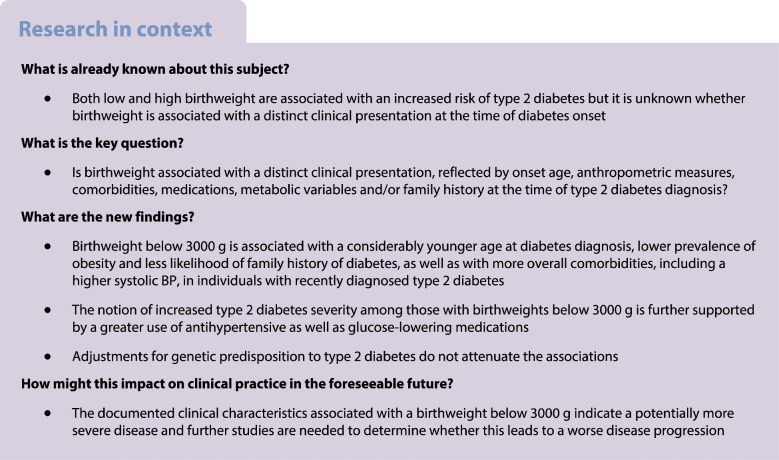


## Introduction

Type 2 diabetes is a multifactorial heterogeneous disease associated with a range of complications [1]. There is increasing evidence that both low and high birthweight are associated with increased risk of type 2 diabetes in adult life [2–6]. Furthermore, low birthweight is a known risk factor for hypertension, dyslipidaemia, cardiovascular disease and neurocognitive dysfunction, even among people without type 2 diabetes [2, 7, 8]. In precision medicine, there is a strong focus on understanding clinically relevant disease segmentation and identifying individuals with the highest need for care. With low birthweight thus being a risk factor for not only type 2 diabetes but also for many of the most important type 2 diabetes comorbidities, one may speculate that having a birthweight outside the normal range among individuals with type 2 diabetes is associated with a differential clinical presentation at onset, with more comorbidities.

The thrifty phenotype hypothesis proposes that exposure to an adverse fetal environment may lead to metabolic adaptations that help prepare the low-birthweight child for survival in a sparse environment [9, 10]. However, the same adaptive changes may subsequently become detrimental when the individual is exposed to an affluent lifestyle, increasing the risk of developing type 2 diabetes and associated complications [10]. The theoretical framework of developmental programming involving impaired development of multiple organs, including but not restricted to those involved in glucose homeostasis [2, 9–11], raises the question of whether a lower birthweight in type 2 diabetes may be characterised by a more severe clinical presentation that potentially includes more comorbidities.

We conducted a cross-sectional analysis examining the relationship between lower birthweight and clinical characteristics reflecting disease severity, such as age at diabetes diagnosis, anthropomorphic measures, glucose and lipid metabolism, medications, family history of type 2 diabetes, and comorbidities. Due to reports of a potential U- or J-shaped association between birthweight and risk of type 2 diabetes [2, 8–10], we also examined the impact of high compared with normal birthweight on type 2 diabetes characteristics. Further acknowledging the potential impact of genetics on the associations between birthweight and type 2 diabetes [2, 9, 11], we also used polygenic scores (PS) to assess the quantitative genetic impacts of birthweight and type 2 diabetes on associations between birthweight and clinical characteristics. Finally, using a case–control design, we reaffirmed the association of low birthweight with the risk of type 2 diabetes in the Danish Centre for Strategic Research in Type 2 Diabetes (DD2) cohort.

## Methods

### DD2 cohort

Since November 2010, individuals have been continuously enrolled into the nationwide DD2 cohort by general practitioners (GPs) and hospital outpatient clinics. The enrolment process, implementation, logistics, biobank and characteristics of the cohort have been described previously [12]. In brief, clinicians identify new type 2 diabetes patients and complete an online questionnaire. Urine and fasting blood samples are collected for storage in a biobank. From the beginning, the DD2 cohort aimed to enrol participants with newly diagnosed type 2 diabetes. In practice, however, the referral to DD2 has not always happened at the first identification of diabetes, when other clinical activity and therapy may have been more pertinent. Thus, the average time from the first-recorded glucose-lowering drug initiation after diabetes diagnosis to the enrolment date in the DD2 cohort is approximately 1 year [12]. The unique civil personal registration number assigned to all Danish residents can be used to link DD2 participants to different Danish health registries. Variables collected for the DD2 cohort have been described in previous publications [12, 13] and are presented at www.dd2.dk (accessed 8 March 2023). We do not have detailed information on ethnicity, but all individuals were born in Denmark between 1920 and 1988.

### Exposure: birthweight

The feasibility of extracting birthweight and associated variables for Danish residents using the Danish National Archive (DaNaAr) has been established [14, 15]. For all individuals in the DD2 cohort born in 1920–1988, and where data were potentially available in the DaNaAr, we extracted objectively ascertained midwife information on birthweight, non-singleton birth status and born-at-term status (see electronic supplementary material [ESM] Methods: birthweight data from original midwife records). Our main analysis focused on the lowest and highest quartiles of birthweight on the basis of considerations of statistical precision together with our assumption that associations would be observed in a dose–response relationship [2] across the entire birthweight spectrum. While our a priori hypothesis was that low birthweight would be associated with a more severe clinical presentation, we kept a parallel focus on high birthweight to avoid overlooking potential U- or J-shaped relationships. Birthweight was therefore divided into three categories: individuals below the lowest quartile (<25%, <3000 g); above the highest quartile (>75%, >3700 g); and between the lowest and highest quartile (25–75%, 3000–3700 g, as the reference group). In additional analyses, we reran our models, applying frequently used clinical definitions of low birthweight (often <2500 g) or high birthweight (often >4500 g) [16].

### Population controls

We ascertained birth data for birth-date-matched controls for each DD2 cohort diabetes participant available in the DaNaAr. We identified control individuals by randomly choosing two births from the same page of the midwife paper records where the corresponding individual with diabetes was recorded (one page typically contains birth information on 6–8 consecutive deliveries). This allowed for matching of diabetes cases and controls to the closest available date of birth (within the same week), geographic location, hospital and individual midwife (ESM Methods: birthweight data from original midwife records).

### Outcomes and covariates

Information on outcomes, covariates, definitions and codes is provided in ESM Table 1 [12]. This includes age at diagnosis, family history of type 2 diabetes, anthropomorphic measures (height, weight, BMI, waist circumference, waist/hip and waist/height ratios), BP, glucose and lipid metabolism, and comorbidities and diabetes-associated complications at time of disease onset. Variables were categorised based on prior publications [12]. If their distributions were substantially skewed, they were categorised based on their distributions. We defined outliers as values >5 SDs from the mean (ESM Table 1).

### Statistical analyses

#### Cross-sectional analysis of characteristics associated with birthweight in the diabetes cohort

Descriptive data are provided as medians (IQR) for continuous variables and as counts (percentages) for categorical variables according to the three birthweight categories. To examine the associations between preselected clinical type 2 diabetes variables and birthweight, we performed log-binomial and robust (modified) Poisson regression [17] analyses to calculate prevalence ratios (PRs) with 95% CIs using birthweight <3000 g and birthweight >3700 g as exposures and birthweight 3000–3700 g as the reference category. PRs for all outcomes were adjusted for sex, family history of type 2 diabetes and age at DD2 enrolment (except when focusing on age at diagnosis). We refrained from additional adjustment in our main model because different metabolic and lifestyle factors may act as intermediates in the incompletely understood pathways between birthweight and type 2 diabetes. Further adjustments for BMI, physical activity, smoking and/or alcohol consumption were performed in extended exploratory analyses.

To investigate associations across the full birthweight spectrum, we performed linear regression analyses. To allow for potential non-linear relationships, we used two- and three-degree polynomials and restricted cubic spline regression analyses (ESM Methods: Exploring linear and non-linear relationships). All models were adjusted for sex, family history of type 2 diabetes and age at DD2 enrolment (except when focusing on age at diagnosis). Logarithmic transformations are presented as the percentage change in the outcome per 1000 g birthweight. To account for missing data when performing regression analysis, we employed multivariate imputations by chained equations (MICE) using the MICE package version 3.14.0 from R [18]. The percentage of missing values varied between 0% and 57% (blood lipids), with a mean percentage missing values of 13.75% across all variables. The distribution of missingness was similar across birthweight, sex and age at enrolment. Predictive mean matching was used for imputation of continuous variables, logistic regression for binary variables and polytomous regression for multilevel categorical variables. See ESM Methods (Multivariate imputations by chained equations [MICE] model specification) for further specification of the method and the missing data pattern.

### Additional analyses

#### PS

Based on a DD2 subpopulation with available genotype data (*n=*2563), we calculated PS for developing type 2 diabetes and for higher birthweight [19, 20]. Genotyping was performed using the Global Screening Array-24 v2.0 (Illumina, San Diego, CA, USA). After removing individuals and variants with >5% missingness, the genotype data were imputed using the Haplotype Reference Consortium reference panel build GRCh37. PS was calculated using published weighted scores [19, 20]. We were able to include 94% of the variants in the type 2 diabetes PS (PGS000014) and 100% of the variants in the birthweight PS (PGS002105). We performed log-binomial regression analyses to calculate PRs with 95% CIs to investigate the associations between the PS for developing type 2 diabetes and the ascertained midwife record birthweight of the individuals with type 2 diabetes. All main analyses were then repeated in this subpopulation, further adjusting for the type 2 diabetes and birthweight PS. Self-reported information on family history of type 2 diabetes was validated against the PS for developing type 2 diabetes.

#### Case–control analysis of diabetes risk

Finally, in a case–control analysis of 6866 eligible individuals with type 2 diabetes (cases; see later) and the 17,780 birth-date-matched control individuals (ESM Methods: birthweight data from original midwife records), we performed logistic regression analysis to calculate ORs as a measure of relative risks of being diagnosed with type 2 diabetes, using <3000 g and >3700 g as exposures, and 3000–3700 g as the reference. We further re-analysed associations using conventional clinical cut-off points for low and high birthweight (<2500 g and >4500 g, respectively).

#### Sensitivity analyses

To investigate the potential impact of preterm birth on birthweight associations, we additionally adjusted our models for the variable ‘born-at-term’. We re-analysed the data using conventional clinical cut-off points for low and high birthweight (<2500 g and >4500 g). We further reran our analyses comparing individuals below the lowest and above the highest birthweight quartile with those between birthweight quartiles, while using observed quartiles specific to four subgroups: male and born-at-term (<25% [3200 g] and >75% [3800 g]); male and not born-at-term (<25% [2275 g] and >75% [2800 g]); female and born-at-term (<25% [3100 g] and >75% [3685 g]); and female and not born-at-term (<25% [2250 g] and >75% [2800 g]).

All analyses were performed using R statistical software version 4.1.2 [21]. We followed the Strengthening the Reporting of Observational Studies in Epidemiology (STROBE) guidelines.

### Ethics

This study was approved by the Danish Data Protection Agency (record number 2008–58–0035) and by the Regional Committees on Health Research Ethics for Southern Denmark (record number S-20100082). All cohort participants gave written informed consent.

## Results

**Fig. 1:**
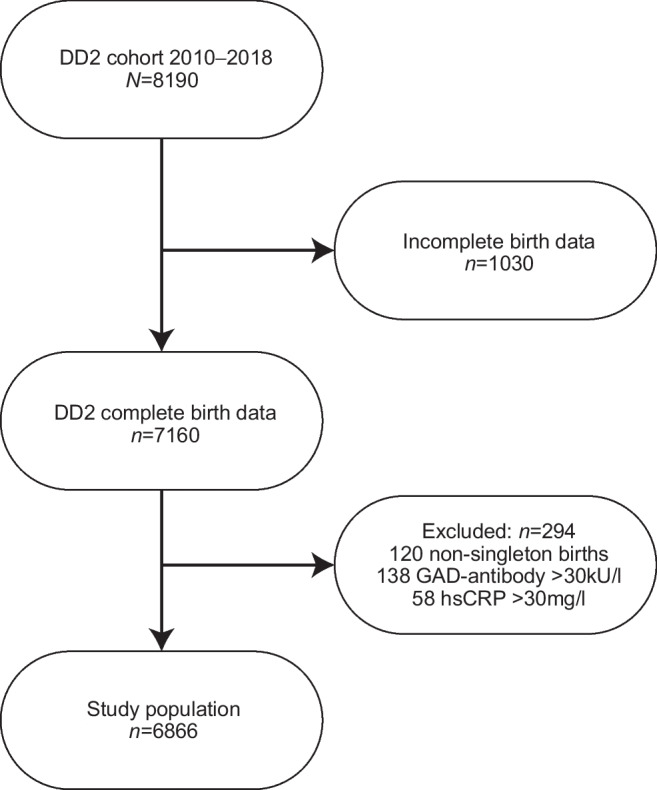
Flowchart of study population selection. Individuals may have fulfilled more than one of the exclusion criteria at DD2 enrolment

The DD2 cohort enrolled 8190 participants in the period 2010–2018. A total of 1030 participants were born after 1988, had an unknown birthplace or had incomplete birth data, leaving 7160 (87%) for whom we were able to trace complete midwife records. We excluded 294 individuals with non-singleton births, positive GAD (GAD) antibody (>30 kU/l) (to avoid potential misclassification of type 2 diabetes with autoimmune diabetes) or high-sensitivity C-reactive protein (hsCRP) >30 mg/l (indicating acute illness at time of enrolment). Our final study population included 6866 individuals with type 2 diabetes (Fig. 1), of whom *n=*1675 (24.4%) had a birthweight <3000 g, *n=*3525 (51.3%) had a birthweight of 3000–3700 g, and *n=*1666 (24.3%) had a birthweight >3700 g. Table 1 shows baseline characteristics overall and according to birthweight category. Median age at enrolment was 62 years; 8.4% were diagnosed with type 2 diabetes at <45 years of age and 8.0% at ≥75 years of age. The proportion of women was 41.2%, and 54.5% of the participants were enrolled by GPs. Further information on covariates according to birthweight categories can be found in ESM Tables 2–4.

**Table 1:** Baseline characteristics according to birthweight

Enrolment characteristic	<3000 g (*n*=1675)	3000–3700 g (*n*=3525)	>3700 g (*n*=1666)	Total (*n*=6866)
Sex				
Male	810 (48.36)	2099 (59.55)	1128 (67.71)	4037 (58.80)
Female	865 (51.64)	1426 (40.45)	538 (32.29)	2829 (41.20)
Age at enrolment, years	59.3 (51.30–66.51)	62.09 (53.46–68.39)	64.53 (55.61–70.08)	61.95 (53.18–68.52)
Age category at enrolment				
<45 years	183 (10.93)	296 (8.40)	100 (6.00)	579 (8.43)
45–55 years	438 (26.15)	707 (20.06)	298 (17.89)	1443 (21.02)
55–65 years	549 (32.78)	1153 (32.71)	465 (27.91)	2167 (31.56)
65–75 years	408 (24.36)	1097 (31.12)	620 (37.21)	2125 (30.95)
≥75 years	97 (5.79)	272 (7.72)	183 (10.98)	552 (8.04)
Age at diagnosis, years	57.16 (49.35–64.50)	59.83 (51.55–66.47)	62.15 (53.47–68.07)	59.8 (51.3–66.5)
Age category at diagnosis				
<45 years	241 (14.39)	410 (11.63)	139 (8.34)	790 (11.51)
45–55 years	483 (28.84)	787 (22.33)	330 (19.81)	1600 (23.30)
55–65 years	559 (33.37)	1249 (35.43)	547 (32.83)	2355 (34.30)
65–75 years	331 (19.76)	903 (25.62)	515 (30.91)	1749 (25.47)
≥75 years	61 (3.64)	176 (4.99)	135 (8.10)	372 (5.42)
Family history of type 2 diabetes				
*n*=0	816 (48.72)	1715 (48.65)	759 (45.56)	3290 (47.92)
*n*=1	538 (32.12)	1115 (31.63)	560 (33.61)	2213 (32.23)
*n*=2	250 (14.93)	506 (14.35)	249 (14.95)	1005 (14.64)
*n*=3+	71 (4.24)	189 (5.36)	98 (5.88)	358 (5.21)
Enrolment status				
GP clinic	864 (51.58)	1950 (55.32)	928 (55.70)	3742 (54.50)
Outpatient clinic	811 (48.42)	1575 (44.68)	738 (44.30)	3124 (45.50)
Weight^a^, kg	87 (75–100)	92 (81–106)	97 (86.0–111.8)	92 (80.7–106.0)
Weight category^a^				
<80 kg	452 (34.53)	695 (24.69)	218 (16.16)	1365 (24.94)
80–106 kg	632 (48.28)	1421 (50.48)	696 (51.59)	2749 (50.23)
≥106 kg	225 (17.19)	699 (24.83)	435 (32.25)	1359 (24.83)
Height^a^, cm	170 (163–176)	173 (166–179)	176 (169–182)	173 (166–180)
Height category^a^				
<166 cm	594 (37.98)	828 (25.21)	286 (18.31)	1708 (26.64)
166–180 cm	784 (50.13)	1785 (54.34)	778 (49.81)	3347 (52.21)
≥180 cm	186 (11.89)	672 (20.46)	498 (31.88)	1356 (21.15)
BMI^a^, kg/m^2^	30.02 (26.67–33.98)	30.64 (27.26–34.97)	31.42 (28.08–35.50)	30.76 (27.34–34.89)
BMI category^a^				
<25 kg/m^2^	586 (34.99)	1112 (31.55)	478 (28.69)	2176 (31.69)
25–30 kg/m^2^	447 (26.69)	911 (25.84)	387 (23.23)	1745 (25.42)
30–35 kg/m^2^	378 (22.57)	817 (23.18)	433 (25.99)	1628 (23.71)
35–40 kg/m^2^	181 (10.81)	423 (12.00)	237 (14.23)	841 (12.25)
≥40 kg/m^2^	83 (4.96)	262 (7.43)	131 (7.86)	476 (6.93)
Waist circumference, cm	104 (95–114)	107 (98–117)	110 (100–120)	107 (97–117)
Waist circumference category				
<94 cm male/<80 cm female	202 (12.06)	336 (9.53)	117 (7.02)	655 (9.54)
94–102 cm male/80–88 cm female	235 (14.03)	485 (13.76)	195 (11.70)	915 (13.33)
≥102 cm male/≥88 cm female	1238 (73.91)	2704 (76.71)	1354 (81.27)	5296 (77.13)
Waist/hip ratio	0.97 (0.91–1.03)	0.98 (0.92–1.04)	1 (0.93–1.05)	0.98 (0.92–1.04)
Waist/hip ratio category				
<0.92	491 (29.33)	827 (23.54)	341 (20.51)	1659 (24.22)
0.92–1.04	841 (50.24)	1787 (50.87)	868 (52.19)	3496 (51.04)
≥1.04	342 (20.43)	899 (25.59)	454 (27.30)	1695 (24.74)
Waist/height ratio^a^	0.61 (0.56–0.67)	0.62 (0.56–0.68)	0.62 (0.57–0.68)	0.62 (0.56–0.68)
Waist/height ratio category^a^				
<0.5	104 (6.64)	201 (6.13)	73 (4.67)	378 (5.90)
0.5–0.6	625 (39.91)	1177 (35.87)	551 (35.28)	2353 (36.71)
≥0.6	837 (53.45)	1903 (58.00)	938 (60.05)	3678 (57.39)
Alcohol consumption				
≤21/14 units per week male/female	1592 (95.04)	3266 (92.65)	1557 (93.46)	6415 (93.43)
>21/14 units per week male/female	83 (4.96)	259 (7.35)	109 (6.54)	451 (6.57)
Smoking status^a^				
Never	557 (48.48)	1130 (46.08)	496 (42.03)	2183 (45.66)
Former	352 (30.64)	890 (36.30)	448 (37.97)	1690 (35.35)
Current	240 (20.89)	432 (17.62)	236 (20.00)	908 (18.99)
Physical activity				
0 days per week	239 (14.27)	534 (15.15)	245 (14.71)	1018 (14.83)
1–2 days per week	351 (20.96)	683 (19.38)	329 (19.75)	1363 (19.85)
3–4 days per week	390 (23.28)	793 (22.50)	389 (23.35)	1572 (22.90)
5–6 days per week	254 (15.16)	547 (15.52)	222 (13.33)	1023 (14.90)
7 days per week	441 (26.33)	968 (27.46)	481 (28.87)	1890 (27.53)

Data are shown as *n* (%) or median (IQR)

^a^Contains missing data. The distribution of missing data can be found in ESM Methods: Multivariate imputations by chained equations (MICE) model specification

### Age at diagnosis, family history of type 2 diabetes, and body composition

**Fig. 2:**
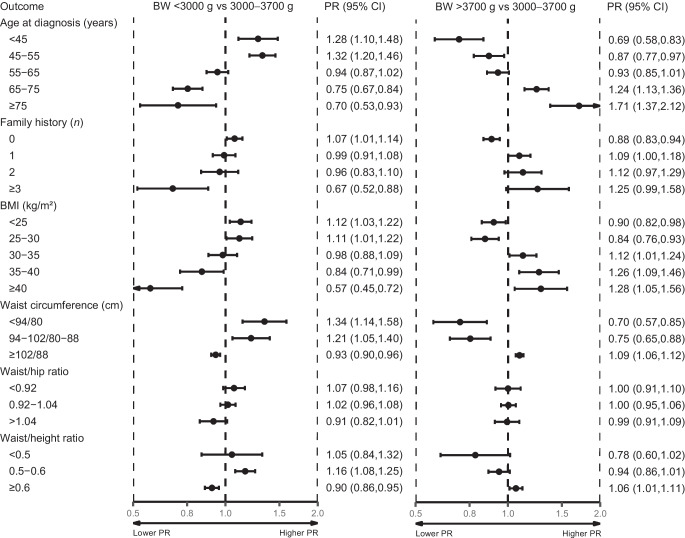
Forest plot of age at type 2 diabetes diagnosis, family history of type 2 diabetes, and body composition. Adjusted PRs for age at diagnosis, anthropomorphic measures (BMI, waist circumference, waist/hip ratio and waist/height ratio), and family history of type 2 diabetes according to birthweight are shown. Age at diagnosis was only adjusted for sex and family history of type 2 diabetes. Family history of type 2 diabetes was adjusted for sex and age at enrolment. Body composition variables were adjusted for sex, age at enrolment and family history of type 2 diabetes. Waist circumference is shown for male/female participants Birthweight categories included a population of 1675 for <3000 g, 3525 for 3000–3700 g and 1666 for >3700 g

Compared with birthweight 3000–3700 g, birthweight <3000 g was associated with younger age at type 2 diabetes diagnosis (Fig. 2). The PR for age <45 years was 1.28 (95% CI 1.10, 1.48), and the PR for age ≥75 years was 0.70 (95% CI 0.53, 0.93). Birthweight <3000 g was associated with reporting fewer individuals with a family history of type 2 diabetes, with a PR of 1.07 (95% CI 1.01, 1.14) for reporting no type 2 diabetes-affected relatives and a PR of 0.67 (95% CI 0.52, 0.88) for reporting three or more relatives with type 2 diabetes (Fig. 2). Similarly, birthweight <3000 g was associated with a lower BMI, with a PR of 1.12 (95% CI 1.03, 1.22) for BMI <25 kg/m^2^, decreasing to a PR of 0.57 (95% CI 0.45, 0.72) for presence of severe obesity (≥40 kg/m^2^). Participants with birthweight <3000 g also had a smaller waist circumference, with a PR of 1.34 (95% CI 1.14, 1.58) for male participants with a waist circumference of <94 cm/female participants with waist circumference <80 cm and a PR for waist/height ratio of 0.90 (95% CI 0.86, 0.95) for a ratio ≥0.6 (Fig. 2).

Compared with birthweight 3000–3700 g, birthweight >3700 g was associated with older age at type 2 diabetes diagnosis, with a PR for ≥75 years of 1.71 (95% CI 1.37, 2.12) (Fig. 2). Birthweight >3700 g was also associated with more family history of type 2 diabetes (PR for three or more affected relatives 1.25 [95% CI 0.99, 1.58]), higher BMI (PR for ≥ 40 kg/m^2^ was 1.28 [95% CI 1.05, 1.56]), larger waist circumference (PR for ≥102 cm [male]/88 cm [female] was 1.09 [95% CI 1.06, 1.12]) and higher waist/height ratio (PR for ≥0.6 was 1.06 [95% CI 1.01, 1.11]). No clear associations for birthweight <3000 g or >3700 g were observed for waist/hip ratio. All associations remained robust after further adjustment for BMI, alcohol, smoking status and physical activity (ESM Table 5).

### Hypertension and blood lipids

**Fig. 3:**
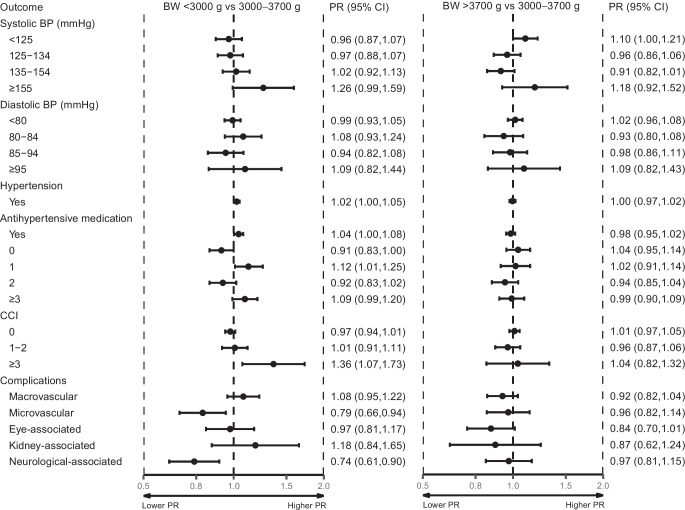
Forest plot of BP, comorbidities and diabetes-associated complications. Adjusted PRs for BP variables, comorbidities and diabetes-associated complications according to birthweight are shown. Adjusted for sex, age at enrolment and family history of type 2 diabetes. Birthweight categories included a population of 1675 for <3000 g, 3525 for 3000–3700 g and 1666 for >3700 g

Compared with birthweight 3000–3700 g, birthweight <3000 g was associated with higher systolic BP (SBP) (PR for ≥155 mmHg was 1.26 [95% CI 0.99, 1.59)] and with use of three or more antihypertensive drugs (PR 1.09 [95% CI 0.99, 1.20]) (Fig. 3). After further adjustment for BMI, alcohol, smoking status and physical activity, these associations became stronger (ESM Table 5). Compared with birthweight 3000–3700 g, birthweight >3700 g was associated with lower SBP (PR for <125 mmHg was 1.10 [95% CI 1.00, 1.21]) (Fig. 3) and with lower total cholesterol (PR for <3.70 mmol/l was 1.16 [95% CI 1.03, 1.32]) (ESM Fig. 1). These associations remained after adjusting for BMI, alcohol, smoking and physical activity (ESM Table 5). No associations of birthweight <3000 g or >3700 g were observed for diastolic BP (DBP) (Fig. 3), triglycerides, HDL-cholesterol, LDL-cholesterol or use of lipid-lowering drugs (ESM Fig. 1). Total cholesterol was associated only with high birthweight (ESM Fig. 1, ESM Table 5).

### Metabolic variables

Compared with birthweight 3000–3700 g, birthweight <3000 g was associated with a greater use of glucose-lowering drugs (PR for use of three or more medications was 1.33 [95% CI, 1.06, 1.65]) (ESM Fig. 1). No clear associations were observed for measures of glucose homeostasis (blood glucose, HbA_1c_, C-peptide, HOMA2 insulin sensitivity (IS), HOMA2-B or hsCRP (ESM Fig. 1, ESM Table 5). Compared with birthweight 3000–3700 g, birthweight >3700 g was associated with higher C-peptide levels (PR for ≥1550 pmol/l was 1.14 [95% CI 1.02, 1.27]) and with a higher HOMA2-B (PR for >121 was 1.15 [95% CI 1.03, 1.28]) (ESM Fig. 1). After further adjusting for BMI, alcohol, smoking and physical activity, this association remained for C-peptide but not for HOMA2-Beta (ESM Table 5).

### Comorbidities and diabetes complications

Compared with birthweight 3000–3700 g, birthweight <3000 g was associated with a greater burden of comorbidity, assessed by a Charlson Comorbidity Index (CCI) score of ≥3 (PR 1.36 [95% CI 1.07, 1.73]) (Fig. 3). With the qualification that PRs for individual diseases in the CCI would be too imprecise, we found that, in crude numbers, a greater proportion of participants with birthweight <3000 g, compared with birthweight 3000–3700 g, had myocardial infarctions, congestive heart failure, chronic pulmonary disease, and any malignant tumour at enrolment (ESM Table 4). Those with birthweight <3000 g were less likely to have been diagnosed with microvascular complications (PR 0.79 [95% CI 0.66, 0.94]) and specifically diabetes-associated neurological disease (PR 0.74 [95% CI, 0.61, 0.90]). No clear association was found for macrovascular complications or diabetes-associated eye or renal disease (Fig. 3). All associations remained after further adjustment for BMI, alcohol, smoking status and physical activity (ESM Table 5). For birthweight >3700 g compared with birthweight 3000–3700 g, no clear association was found for CCI or diabetes-associated complications (Fig. 3, ESM Table 5).

### Linear regression analyses

**Fig. 4:**
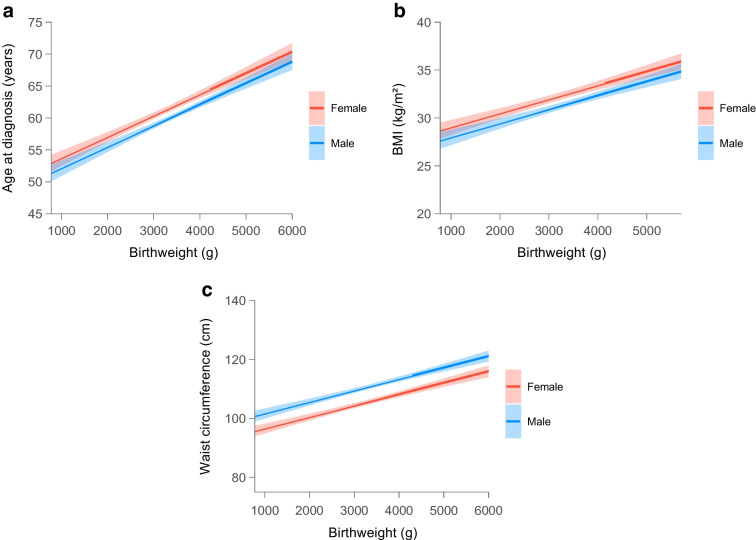
Linear regression plots of age at diagnosis (**a**), BMI (**b**) and waist circumference (**c**) according to birthweight stratified by sex and adjusted for age at enrolment and family history of type 2 diabetes. Estimate shows change in outcome per g change in birthweight with 95% CIs. Models were performed on the study population of 6866 individuals

In a linear regression model, each 1000 g decrease in birthweight was associated with a 3.33 year (95% CI 2.86, 3.80) younger age at type 2 diabetes diagnosis, a 1.46 kg/m^2^ (95% CI 1.19, 1.73) lower BMI and a 3.90 cm (95% CI 3.26, 4.54) smaller waist circumference (Fig. 4a–c and ESM Table 6). These associations became stronger after further adjustment for BMI, alcohol, smoking status and physical activity (ESM Table 6). Finally, in linear regression models, no clear associations were found between birthweight and SBP, DBP, triglycerides, LDL-cholesterol, HDL-cholesterol, HbA_1c_, C-peptide, hsCRP, HOMA2-IS, or HOMA2-B (ESM Table 6). Sensitivity analyses using two- and three-degree polynomials, and restricted cubic spline regression models, were consistent with the linear regression results (ESM Methods: Exploring linear and non-linear relationship).

### Extended analyses

#### PS

Adjusting for type 2 diabetes and/or birthweight PS in the subpopulation with available genotypes did not attenuate any associations (ESM Table 7). No associations between type 2 diabetes PS and the three birthweight groups were observed, nor when using a linear regression model (ESM Fig. 2, ESM Tables 7 and 8). Type 2 diabetes PS was associated with reduced likelihood of reporting no family history of type 2 diabetes (PR 0.94 [95% CI 0.90, 0.98]), and with increased likelihood of reporting multiple relatives with type 2 diabetes (PR for three or more affected relatives was 1.48 [95% CI 1.24, 1.75]) (ESM Fig. 2).

#### Case–control analysis of diabetes risk

**Fig. 5:**
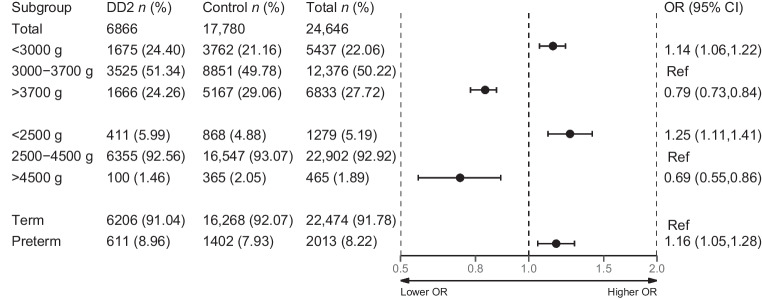
Forest plot of case–control analysis of diabetes risk. All DD2 participants included in the study are considered cases. All individuals in the matched control population are considered controls with the assumptions of being alive at enrolment of their matched DD2 participants and being free from type 2 diabetes. Logistic regressions were used to compute ORs for the association of birthweight with a type 2 diabetes diagnosis. Analysis was adjusted for sex and year at birth

Compared with participants with birthweight 3000–3700 g, those with birthweight <3000 g had a sex- and year-of-birth-adjusted OR of 1.14 (95% CI 1.06, 1.22) of being diagnosed with type 2 diabetes (Fig. 5). Conversely, participants with birthweight >3700 g had an adjusted OR of 0.79 (95% CI 0.73, 0.84) of being diagnosed with type 2 diabetes. Using clinical definitions of low and high birthweight yielded stronger associations with diabetes risk (Fig. 5).

#### Sensitivity analysis

Adjusting the main model further for born-at-term status did not attenuate the associations (ESM Table 9). Re-analysing our data using clinical definitions of low and high birthweight yielded similar or stronger associations compared with our main analyses (ESM Table 10). Further re-analysing our data with sex and born-at-term birthweight categories yielded similar associations (ESM Table 11).

## Discussion

Compared with participants with type 2 diabetes falling within the 50% middle range of birthweight, participants with the lowest 25% birthweight were younger at disease onset, had a lower prevalence of obesity, and a greater burden of comorbidity including an SBP ≥155 mmHg. These participants also displayed greater use of glucose-lowering and antihypertensive medications and were less likely to have a diabetes-associated neurological disease at enrolment. Additionally, participants with birthweight <3000 g were less likely to report a family history of type 2 diabetes. Opposite relationships were generally observed for participants with type 2 diabetes who were in the 25% highest compared with lowest birthweight groups. Adjustment for currently known genetic predispositions to type 2 diabetes and birthweight, using PS, did not attenuate the associations. Finally, the case–control analysis reconfirmed the association between low birthweight and type 2 diabetes risk in the current study population.

The extent to which a lower birthweight in type 2 diabetes is associated with a distinct subphenotype has previously only been studied in 1509 individuals born in the limited time between 1952 and 1966 [22]. Our findings, that a lower birthweight is associated with younger age and less obesity at type 2 diabetes onset, are in accordance with Paulina et al’s results [22]. However, because the DD2 cohort is not restricted to any narrow age interval, we were able to document a more than threefold higher estimate of the impact of 1000 g birthweight on age at type 2 diabetes diagnosis, when compared with Paulina et al (i.e. 3 years vs 0.8 years).

Despite younger age at diagnosis, participants with type 2 diabetes with birthweight <3000 g had an increased CCI score of ≥3 compared with those with birthweight 3000–3700 g. Several CCI diseases are known type 2 diabetes comorbidities as well as being diseases associated with low birthweight independent of type 2 diabetes [2, 8, 11, 15, 23–31]. Despite a greater use of antihypertensive medications, a birthweight <3000 g was also associated with an SBP ≥155 mmHg. Besides type 2 diabetes, hypertension is probably the disease most consistently associated with low birthweight [32, 33]. The pathophysiology of type 2 diabetes involves multiple organ dysfunctions [11], and the associations between low birthweight and several diseases [2, 7, 8] besides type 2 diabetes may likewise represent manifestations of aberrant organ development beyond those involved in glucose homeostasis.

A birthweight <3000 g was associated with a lower prevalence of diabetes-associated neurological disease, while it had no impact on the prevalence of diabetes-associated eye or renal disease. However, given their younger age at diagnosis, increased SBP, increased antihypertensive drug usage, and greater use of glucose-lowering therapies for similar glucose levels, participants with type 2 diabetes with lower birthweight (<3000 g) may be at increased risk of both micro- and macrovascular complications with increasing duration of diabetes [34].

Interestingly, a previous study of only 177 individuals with type 2 diabetes reported increased mortality rate among those with both lowest and highest birthweight [27]. While this supports the finding of an increased disease severity among individuals with the lowest birthweights, and to some extent also the reports of U- or J-shaped associations between birthweight and risk of developing type 2 diabetes, it does not support our findings of a relatively milder clinical disease presentation among those individuals with the highest birthweights. However, further data are needed to understand this and both type 2 diabetes and its comorbidities are in general more associated with lower as opposed to higher birthweight.

The fetal insulin hypothesis proposes that the association between low birthweight and type 2 diabetes could be confounded by genetic factors underlying both low birthweight and increased type 2 diabetes risk [8]. However, after adjustment for known genetic predispositions to type 2 diabetes and birthweight using PS, all associations remained unchanged or even strengthened (ESM Table 7). Moreover, the findings that the participants with type 2 diabetes with birthweight <3000 g less frequently reported a positive family history of type 2 diabetes and, conversely, that those with birthweight >3700 g more frequently reported a family history of type 2 diabetes, further support the conclusion that enrichment for genetic variants associated with type 2 diabetes is unlikely to explain the differential characteristics observed. Interestingly, recorded family history of type 2 diabetes was closely associated with type 2 diabetes PS (ESM Fig. 2), providing cross-validation of both measures as accurately reflecting the genetic predisposition to type 2 diabetes.

Strengths of this study include a large well-characterised cohort of individuals recently diagnosed with type 2 diabetes, with more than 50% recruited from Denmark’s primary healthcare sector. Birthweights were ascertained from digitised original midwife records. Adjustments for the currently known genetic predisposition to type 2 diabetes and birthweight (PS), as well as the finding of fewer individuals with a family history of type 2 diabetes among the lower-birthweight participants with type 2 diabetes, do not suggest that the associations are genetically determined. Results obtained using categorical birthweight groups were supported by continuous models.

Limitations include the cross-sectional study design, which has an inherent risk of sampling bias and precludes causal inferences and the exploration of temporal relationships. However, participant inclusion was nationwide and included only those with new-onset type 2 diabetes from both general practice and hospital clinics. Furthermore, birthweight registrations preceded determinations of clinical type 2 diabetes characteristics and our case–control analysis confirmed that low birthweight is associated with type 2 diabetes in this contemporary cohort. This result is noteworthy as the control group included individuals with unknown later type 2 diabetes disease status and (unlike previous studies reporting associations between low birthweight and type 2 diabetes prevalence) included live-born individuals who may have passed away in early childhood due to prematurity and/or low birthweight. Although the association between lower birthweights and younger age at diabetes diagnosis in theory could be explained by increased contact with and use of the healthcare system, this is unlikely to explain the large impact on type 2 diabetes onset age of 3.33 years per 1000 g birthweight. There were missing data for some covariates. However, characteristics with clear associations were missing relatively little data. Due to relevant treatment having already been started at enrolment, many participants had near normalised BP, lipids and/or glucose levels. Specifically, the increased use of glucose-lowering medication may have masked an association between low birthweight and elevated plasma glucose or HbA_1c_ levels. However, increased medication use in its own right reflects disease severity. The finding that individuals with birthweight <3000 g were more likely to have an SBP ≥155 mmHg in the face of increased use of antihypertensive drugs underscores the strength of the association between low birthweight and hypertension burden in those with recently diagnosed type 2 diabetes. Although we did not have information on ethnicity, all patients were born in Denmark, and our study population is therefore likely to be relatively homogeneous. Further studies in other ethnicities are needed to validate our findings. Finally, we lacked information on education, income and potential maternal/paternal factors related to birthweight and metabolic health.

## Conclusion

Birthweight across the entire spectrum was associated with distinct and clinically relevant type 2 diabetes characteristics. Specifically, a birthweight <3000 g was associated with younger age at diagnosis, lower prevalence of obesity, fewer individuals with a family history of type 2 diabetes, and greater use of glucose-lowering medications, as well as a larger burden of comorbidity including hypertension, in individuals recently diagnosed with type 2 diabetes. Further prospective studies are needed to elucidate the impact of birthweight on disease trajectories, comorbidities, complications and mortality in individuals with type 2 diabetes.

## Supplementary Information

Below is the link to the electronic supplementary material.

Supplementary file1 (PDF 6352 KB)

## Data Availability

Danish data protection legislation does not allow sharing of the individual-level personal data used for this study. However, a data dictionary for variables used in the study and analysis code is in preparation and will be shared and made publicly available on the DD2 website, www.dd2.dk (expected in late 2023). Requests to access the Danish health registries used in this study can be sent from researchers at authorised research institutions to the Danish Health Data Authority by e-mail to forskerservice@sundhedsdata.dk. Requests to use the primary collected DD2 data can be made at https://dd2.dk/forskning/ansoeg-om-data.

## Abbreviations

CCI: Charlson comorbidity index score
DaNaAr: Danish National Archive
DD2: Danish Centre for Strategic Research in Type 2 Diabetes
DBP: Diastolic BP
GP: General practitioner
hsCRP: High-sensitivity C-reactive protein
IS: Insulin sensitivity
MICE: Multivariate imputations using chained equations
PR: Prevalence ratio
PS: Polygenic score
SBP: Systolic BP
