# Supplementary material for: Birthweight is associated with clinical characteristics in people with recently diagnosed type 2 diabetes
Source: Diabetologia. 2023 Jun 12;66(9):1680–92. doi: 10.1007/s00125-023-05936-1 (PMC10390374; doi:10.1007/s00125-023-05936-1)
Supplement: Supplementary file 1 — Supplementary file1 (PDF 6352 KB) [file 125_2023_5936_MOESM1_ESM.pdf]

Electronic Supplementary material for: Birthweight is associated with patient clinical characteristics in recently diagnosed type 2 diabetes patients.

Table of contents

ESM Method: Birthweight data from original midwife records: data documentation from the Danish National Archive to the DD2 research group **2**
ESM Method: Exploring linear and non-linear relationships **5**
ESM Method: Multivariate imputations by chained equations (MICE) model specification **11**
ESM Table 1: DD2 inclusion criteria, definitions, and codes **16**
ESM Table 2: Hypertension, lipids, glucose homeostasis, and subclinical inflammation **22**
ESM Table 3: Medication usage **25**
ESM Table 4: Comorbidities and complications **26**
ESM Table 5: Stepwise adjustment in birthweight categorical analysis **29**
ESM Table 6: Stepwise adjustments in linear regression analyses **38**
ESM Table 7: Associations of birthweight with further adjustment for polygenic risk scores **42**
ESM Table 8: Linear regression analyses of polygenic risk scores and birthweight **45**
ESM Table 9: Further adjustments for born-at-term status **46**
ESM Table 10: Prevalence ratios (main analyses) using conventional birthweight definitions **49**
ESM Table 11: Prevalence ratios using sex and born-at-term birthweight categories **52**
ESM Figure 1: Forest plot of lipids, glucose homeostasis, and subclinical inflammation **55**
ESM Figure 2: Forest plot of polygenic risk scores and birthweight **56**
ESM References **57**

ESM Methods

Birthweight data from original midwife records: data documentation from the Danish National Archive to the DD2 research group

The Steno Diabetes Centre partnered with the Danish National Archive to ascertain birth information from original midwife records for the DD2 cohort participants. The majority of the following text has been directly copied (without linguistic edits) from text written in English by native Danish-speaking employees at the Danish National Archive, who in the current context are regarded as third parties.

To obtain precise birth information, three different sets of original midwife records were used. The original midwife, caring for a mother and infant, filled out the birth information on the day of the birth.

The birth data consist of:

- Birthweight
- Birth length
- Born at term: yes/no
- Twin: yes/no

Description of the cohort:

For the Danish National Archive to be able retrieve the correct birth information for the cohort participants, the following criteria had to be fulfilled:

- Being born in the period from ~1920 to 1988
- Being born in Denmark (not including the Faroe Islands or Greenland)
- Being identifiable through the biological mother's name.

After a complete examination of the entire 9549 cohort participants in the Danish Civil Registration System, a total of 8896 individuals matched the criteria and could potentially be documented through relevant sources in the Danish National Archives collection. Those not meeting those criteria included:

- Individuals who born after 1988
- Individuals who born abroad
- Without an official birthplace in the Danish Civil Registration System
- Individuals not identifiable through the biological mother's name.

Sources/methods of retrieving birth data in the Archives:

The following archive series were used:

- Midwife Records (Jordemoderprotokoller) – 1977 [13]
- Birth Reviews (Fødselsanmeldelser) for Copenhagen – 1977, Frederiksberg and Gentofte municipalities, including the Copenhagen district, 1953–1977 [14]
- Birth Reports (Fødselsindberetninger) / Reviews 1968–1988 [14]

The above archive series differ with respect to setup and data records, and for cases where one archive does not document a birth, another archive may contain and supply information. Especially in the period 1968–1988, births were registered most systematically and the archives are therefore more accurate with respect to the Birth Reports. For the births before 1968, the Midwife Records were primarily used with the support of the Birth Reviews.

Twins: When twins have different biological sex, identification in the Midwife Records is relatively simple, due to a box indicating boy or girl. It is more complicated in cases where twins are of the same sex. In these circumstances, the Parish Registers were used. The Parish Registers show, by way of name, which twin is A and B; that is, who was born first (A) and who was born last (B).

Description of the different sources:

1. The Danish Midwife Record system has a long history. In 1861 the midwives were responsible, by law, for keeping records of all the deliveries and births they were involved in. From 1877, they were required to fill in the Midwife Records on the day of birth[15], thus ensuring optimal accuracy of the

data. The predecessor to the Midwife Records was called "Fuldbårenhedsskema", the "At-term chart". This system can be traced, in some parts of the country, back to 1847. The At-term charts include the same type of information as the Midwife Records: the length at birth, weight at birth, and born-at-term status [15]. The Midwife Records are records in a chart ordered according to date and contain the births that a specific midwife and/or hospital/clinic have been involved in. The Midwife Records are very well preserved with only a few missing. It has been noted that there are some missing records from a limited number of named Danish regions and institutions including Usserød Hospital, the areas of Solrød and Køge, as well as in the entire Tønder medical doctor circle.
The Midwife Records are divided into two periods associated with the administrative "medical doctor districts" as well as "medical doctor circles". The medical doctor districts were discontinued in 1915, but the records have been "allowed" to live until they were complete. The medical doctor circles then took over but were smaller in area than the medical doctor districts. They kept the Midwife Records until around 1977.

2. The Birth Review was a paper note from the midwife or hospital/clinics that was sent to the parent's parish; that is, to the Parish Register. Starting in 1828, parents were required to inform the Parish Register of the birth of a child within 2 days after the birth in the cities and within 8 days in the countryside. Later the midwife took over this responsibility, which was implemented first in Copenhagen and later in the rest of the country [15]. The Birth Reviews contain information about each birth, including weight and length. The Birth Reviews are particularly useful in cases where a birth in the Copenhagen municipality or in the Copenhagen County needs to be documented. Due to the higher population density in the area of "Greater Copenhagen", there were larger discrepancies in the use of midwifes. In these circumstances, the Birth Reviews can provide the name of the exact midwife attending the birth, and/or the location of the clinic where the delivery took place. With this information, the exact Midwife Records can then be located.
3. The Birth Reports contain deliveries from the period 1968–1988. They consist of information provided by the midwife and subsequently sent to the Danish Health Authority. These reports contain information similar to that in the Midwife Records. The major difference is in the way in which it was filled out. The Birth Reports are arranged according to county, sorted by date of birth, and divided between males and females. There are a few areas and years that have been lost. In cases where this information has been lost, the Midwife Records were used to supply information up until around 1977.

Proofreading by the Danish National Archives:

Proofreading was primarily focused on ensuring the accuracy of the highest and lowest registered birthweights. In total, 70% (5849) of individual case records have been proofread.

- ≤2500 g [16]: 567 out of 567 = 100% proofread.
- 2501–4500 g: 5163 out of 7663 = 58% proofread.
- ≥4501 g: 116 out of 116 = 100% proofread.
- Twin status: 124 out of 124 = 100% proofread.

In the proofreading phase, there was a particular focus on alignment of the data. In the beginning of the 1900s the most common units of weight in Denmark were pound and length measured in inches. Pound was converted to grams with one pound equal to 500 grams [17]. In the few instances where inches were used, conversion to the length to the nearest complete cm has been registered [17].

The question of "born at term" in the Midwife Records is associated with some uncertainty, as the accuracy of information depends on the midwife's own judgement, and because information in regard to whether an individual was "born at term" can be written in different ways, which have changed over time. The midwife records have one check box for "born at term" and one for "preterm", and "yes" should be written in only one of the boxes. If the midwife for some reason wrote "yes" in both boxes, it was counted as missing data registration. For babies registered as "preterm", midwives could estimate and write how early the child had been born. However, this information was often missing and/or given as a range (e.g., "from 1 to 4 weeks early", etc.), and we therefore could not use the data for exact gestational age. Instead, we constructed an indicator variable "born-at-term: yes/no", from all available information in the records.

Results:

The Danish National Archives has birth information for about 94% or 8363 individual DD2 patients with

- 1186 individuals whose birth data could not be retrieved, and
- 192 incomplete sets of birth data.

In total, there were 8171 DD2 participants with complete birth data.

Control population
For every birth of any DD2 patient, we in addition extracted two random individual births from the same midwife information sheet (containing birth information for, on average, 6–8 different births), with the aim of establishing a control population. By extracting the controls from the same midwife record, they were matched according to date of birth, individual midwife, and geographical location.
This resulted in a total control population of 18,210 individuals, of whose records a total of 13,180 (72.3%) were proofread.

Exploring linear and non-linear relationships.

Spline knots were placed at fixed quantiles of the predictor's marginal distribution [18, 19]. Best models were chosen by visual inspection and lowest Akaike Information Criterion (AIC). If linear regression models had a p-value <0.05 and an AIC lower or similar to that of the other models, then linear regression models were chosen allowing the best possible interpretation of coefficients. Logarithmic transformations are presented as the percentage change in the outcome per 1000 g change in birthweight.

Scatterplots are stratified by sex (male, female), family history of type 2 diabetes (number of reported relatives) and age at enrolment in categories. Scatterplots have been jittered to add a small amount of noise to hide individual identification through outliers.

Default quantiles for knots placement of restricted cubic spline models:

k	Quantiles	Birthweights (g)
3	0.1, 0.5, 0.9	2750, 3400, 4000
4	0.05, 0.35, 0.65, 0.95	2500, 3200, 3550, 4250
5	0.05, 0.275, 0.5, 0.725, 0.95	2500, 3100, 3400, 3700, 4250
6	0.05, 0.23, 0.41, 0.59, 0.77, 0.95	2500, 3000, 3300, 3500, 3750, 4250
7	0.025, 0.1833, 0.3417, 0.5, 0.6583, 0.8167, 0.97	2250, 2950, 3200, 3400, 3600, 3850, 4450

In scatterplots, individual points have been jittered to ensure data protection. All graphs are presented with 95% confidence intervals.

Age at Diagnosis (years)

Age at Diagnosis	AIC
Linear	51949
Polynomial of degree 2	51950
Polynomial of degree 3	51950
Cubic Restricted Spline 3 knots	51950
Cubic Restricted Spline 4 knots	51951
Cubic Restricted Spline 5 knots	51953
Cubic Restricted Spline 6 knots	51955
Cubic Restricted Spline 7 knots	51956

Scatterplots of linear regression analysis

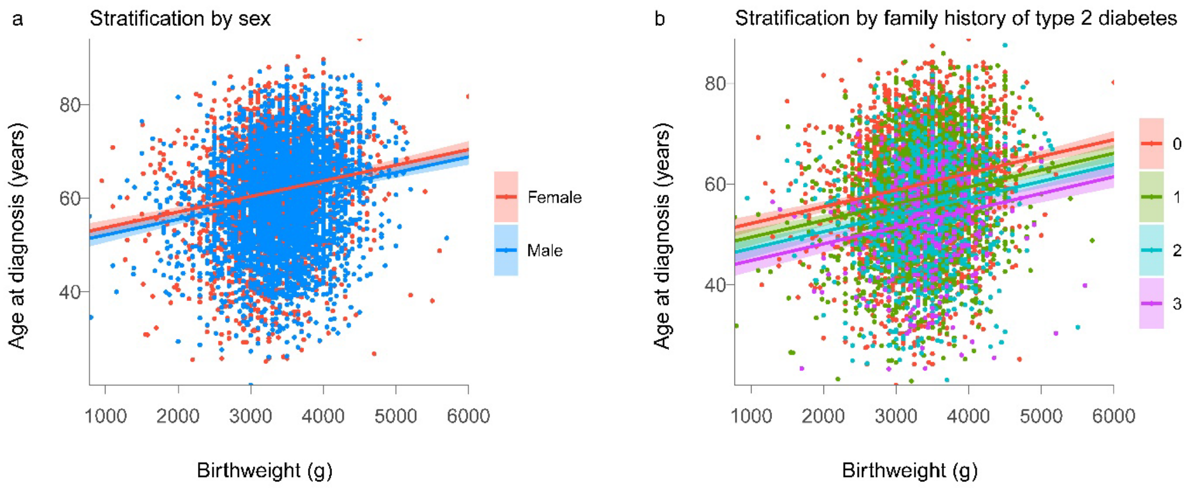

<u>Linear (a), 2-degree polynomial (b), 3-degree polynomial (c), and restricted cubic spline (d) regression plots</u>

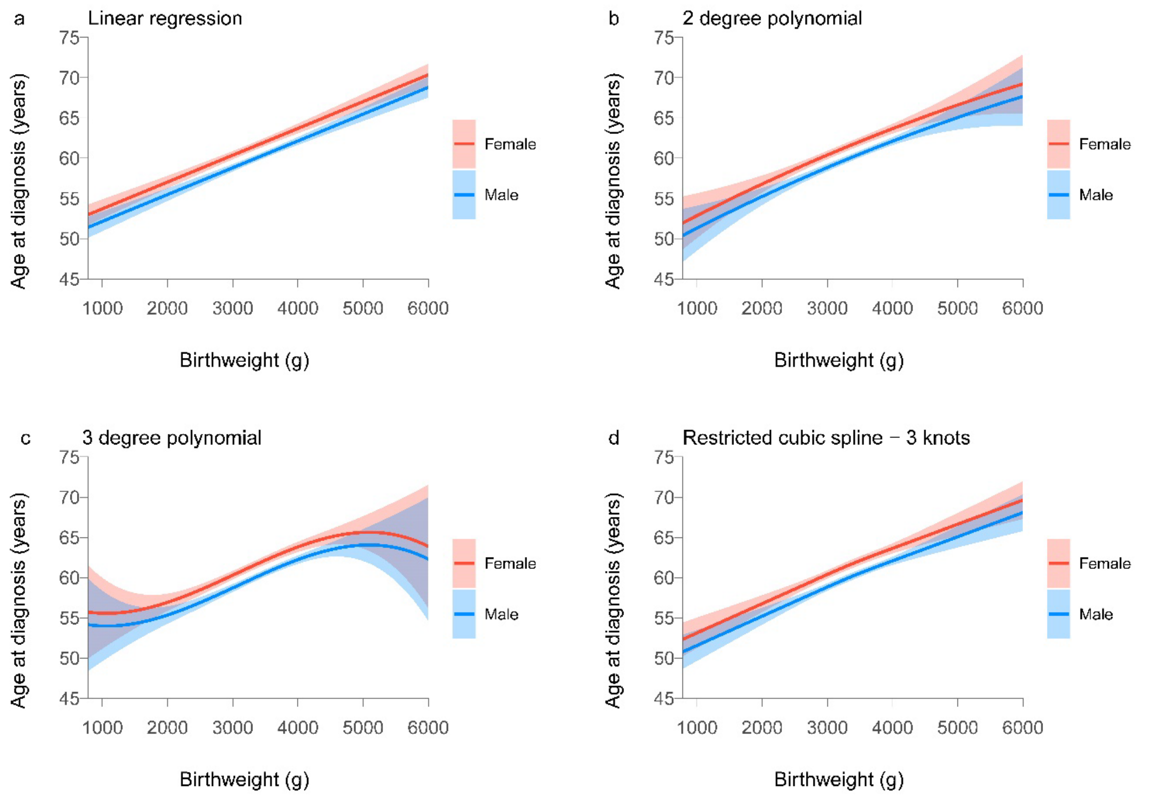

<u>Restricted cubic spline regression plots with 4 (a), 5 (b), 6 (c), and 7 (d) knots.</u>

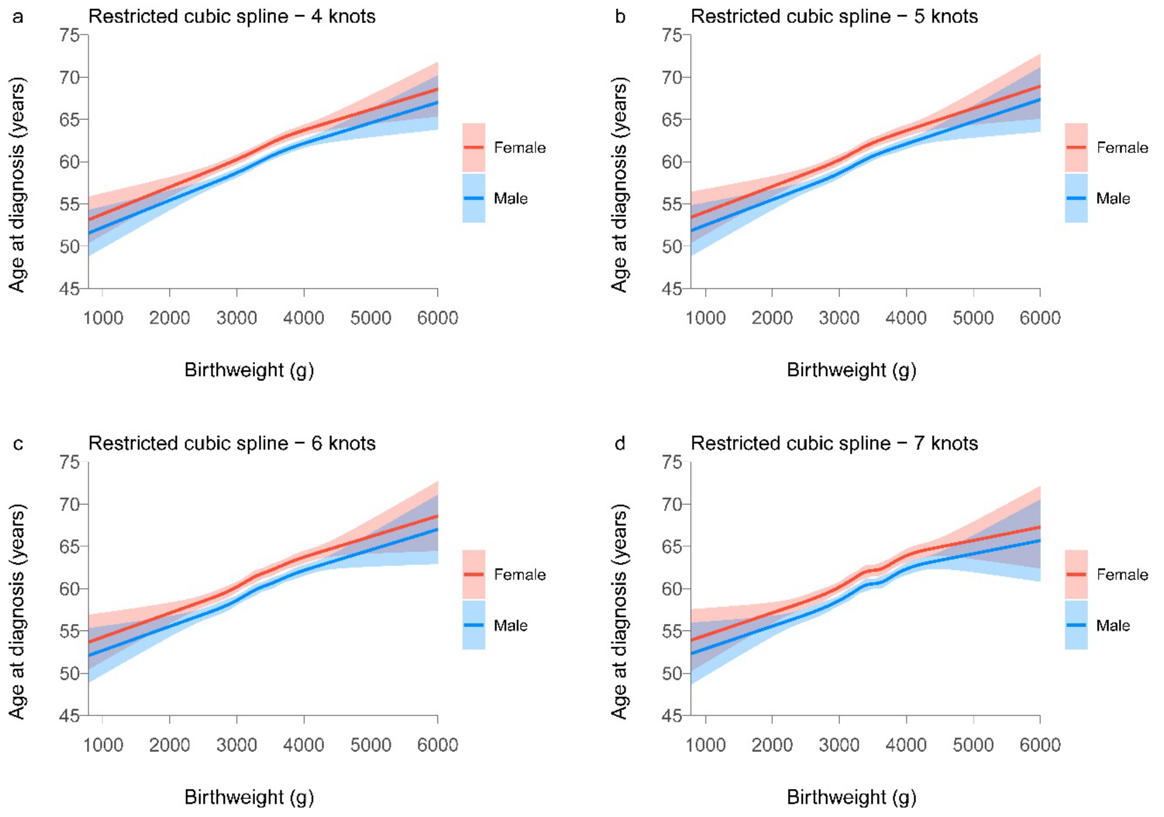

BMI (kg/m^2)

**BMI**	**AIC**
Linear	34068
Polynomial of degree 2	34068
Polynomial of degree 3	34065
Cubic Restricted Spline 3 knots	34069
Cubic Restricted Spline 4 knots	34065
Cubic Restricted Spline 5 knots	34066
Cubic Restricted Spline 6 knots	34067
Cubic Restricted Spline 7 knots	34063

Scatterplots of linear regression analysis

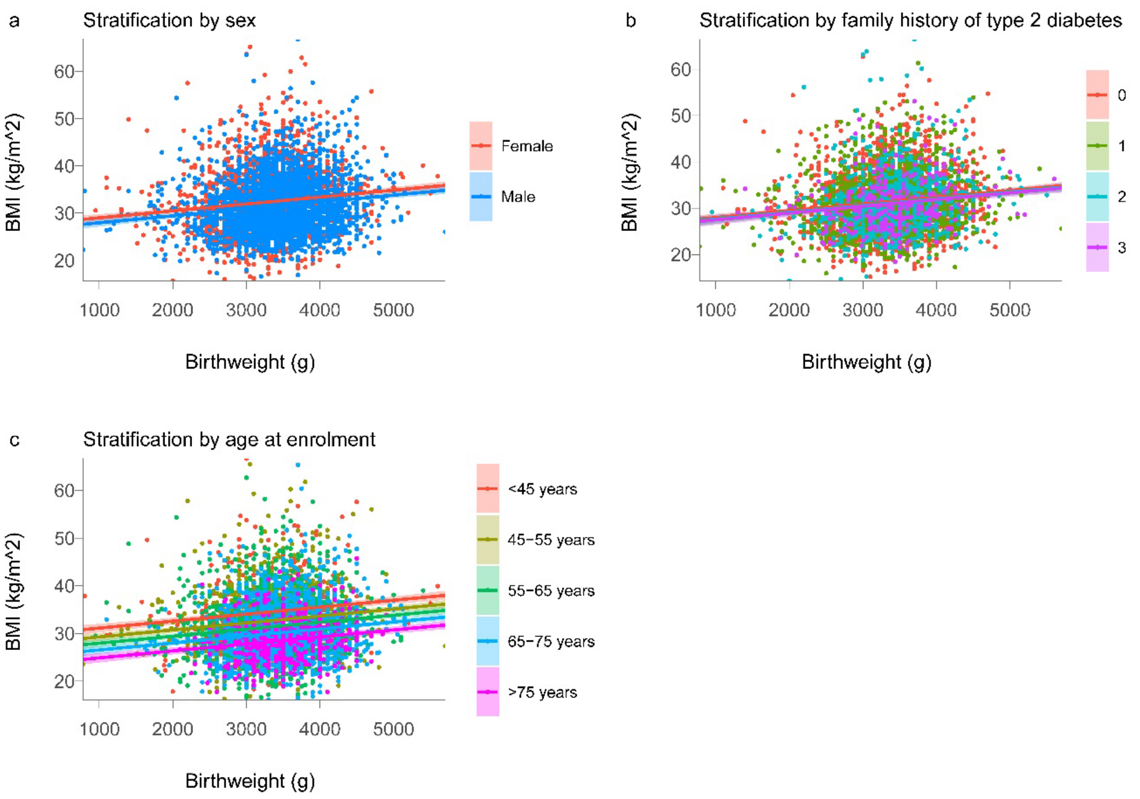

Linear (a), 2-degree polynomial (b), 3-degree polynomial (c), and restricted cubic spline (d) regression plots.

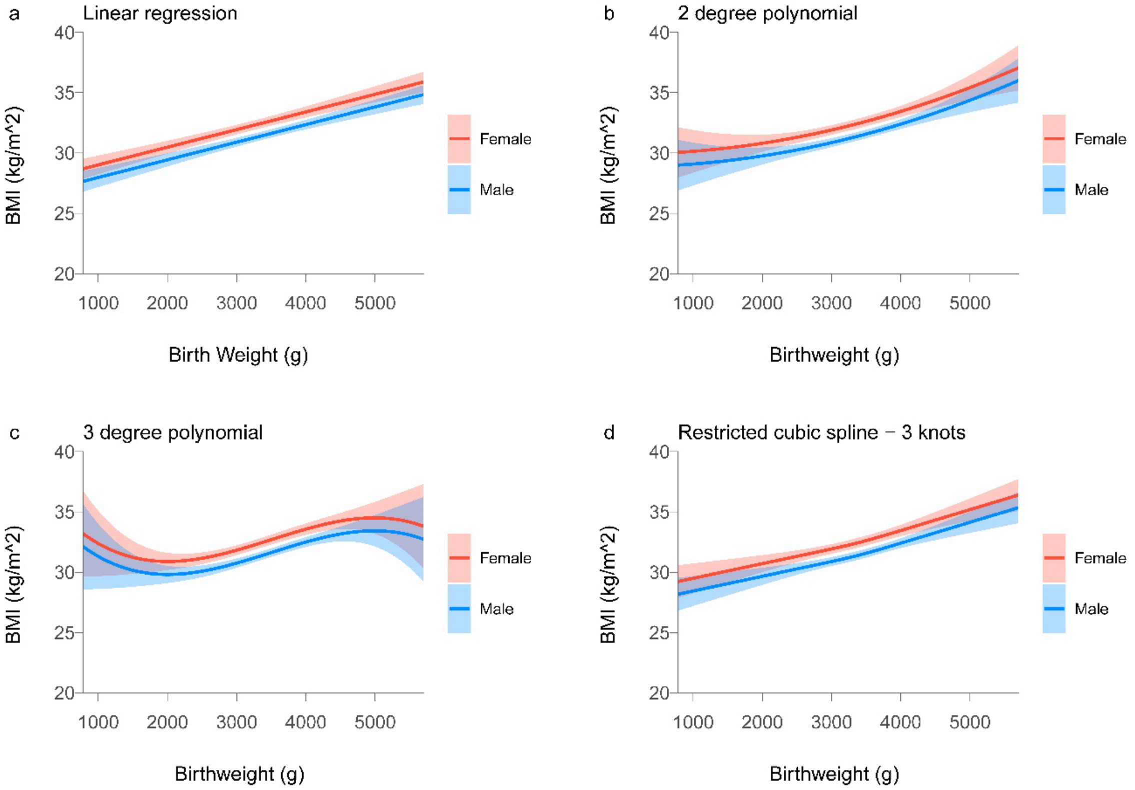

<u>Restricted cubic spline regression plots with 4 (a), 5 (b), 6 (c), and 7 (d) knots.</u>

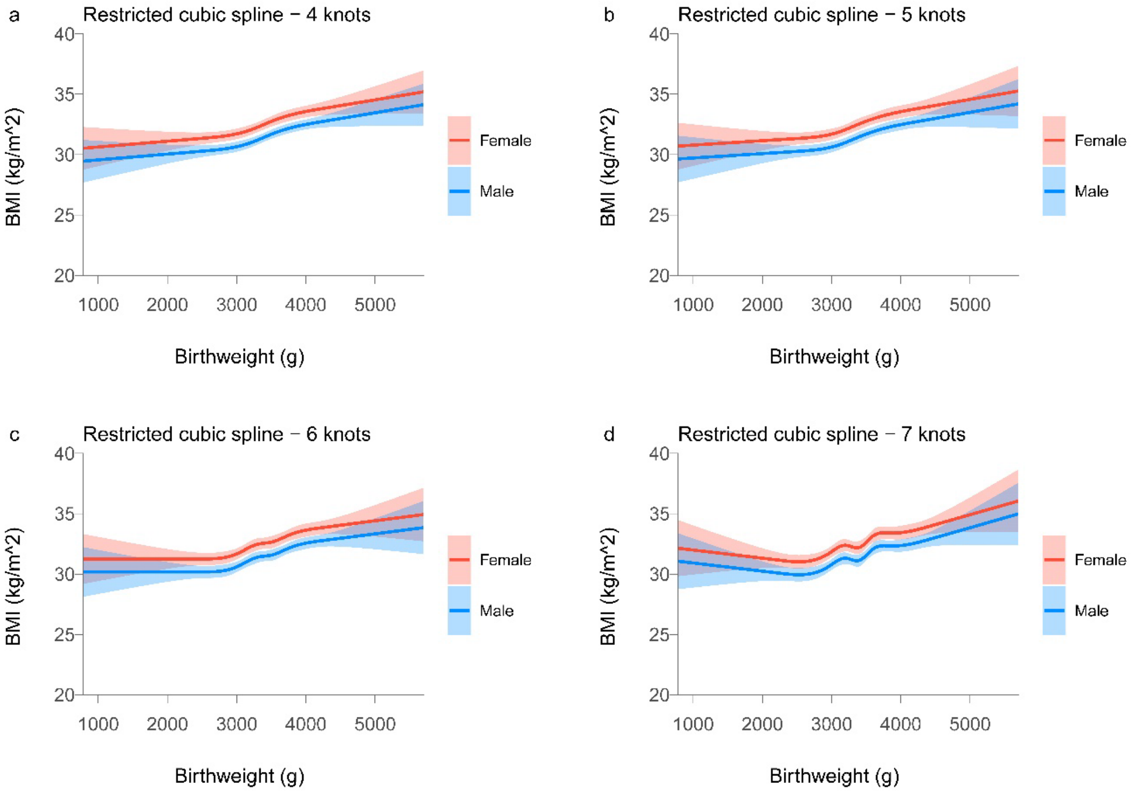

Waist Circumference (cm)

Waist Circumference	AIC
Linear	55816
Polynomial of degree 2	55813
Polynomial of degree 2	55807
Cubic Restricted Spline 3 knots	55814
Cubic Restricted Spline 4 knots	55809
Cubic Restricted Spline 5 knots	55810
Cubic Restricted Spline 6 knots	55810
Cubic Restricted Spline 7 knots	55807

Scatterplots of linear regression analysis

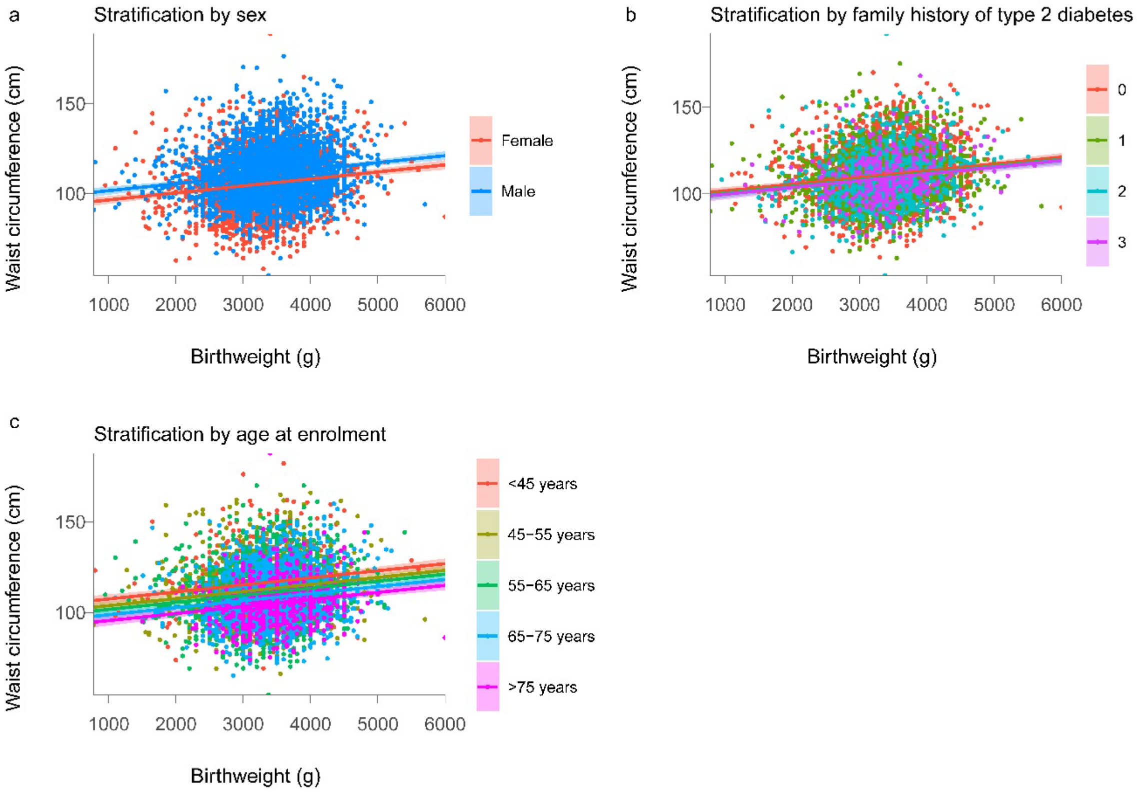

Linear (a), 2-degree polynomial (b), 3-degree polynomial (c), and restricted cubic spline (d) regression plots.

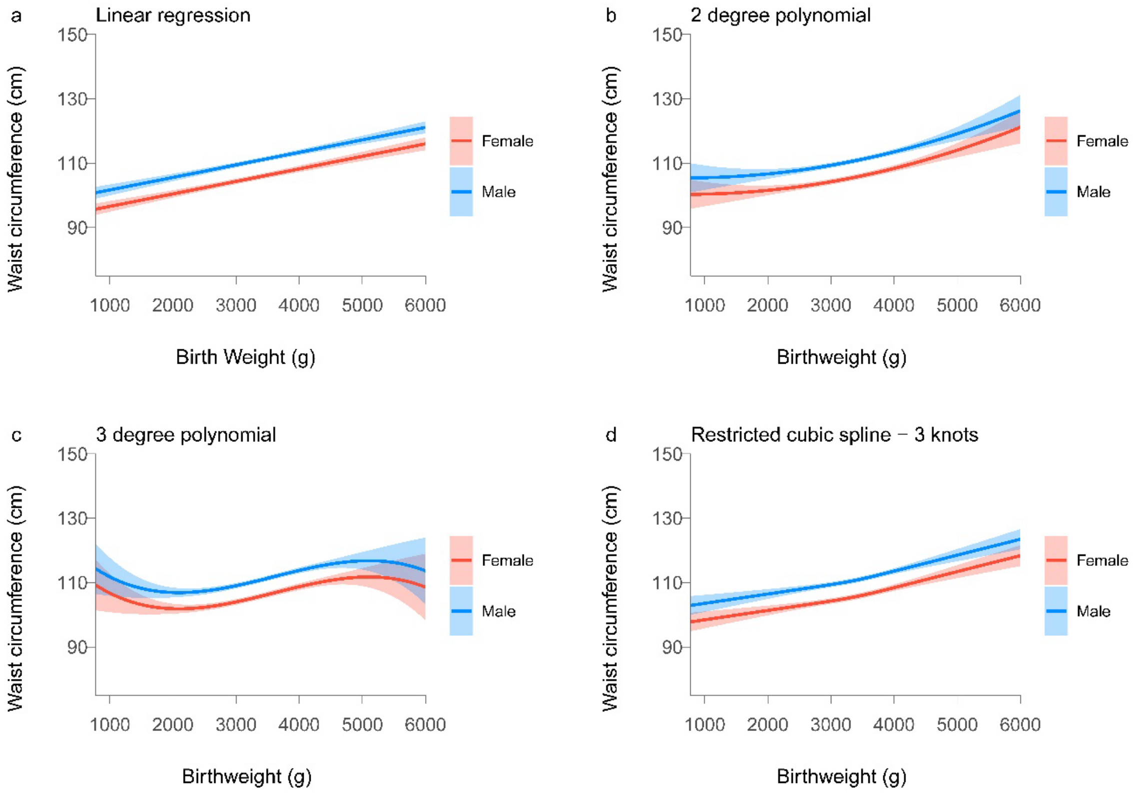

<u>Restricted cubic spline regression plots with 4 (a), 5 (b), 6 (c), and 7 (d) knots.</u>

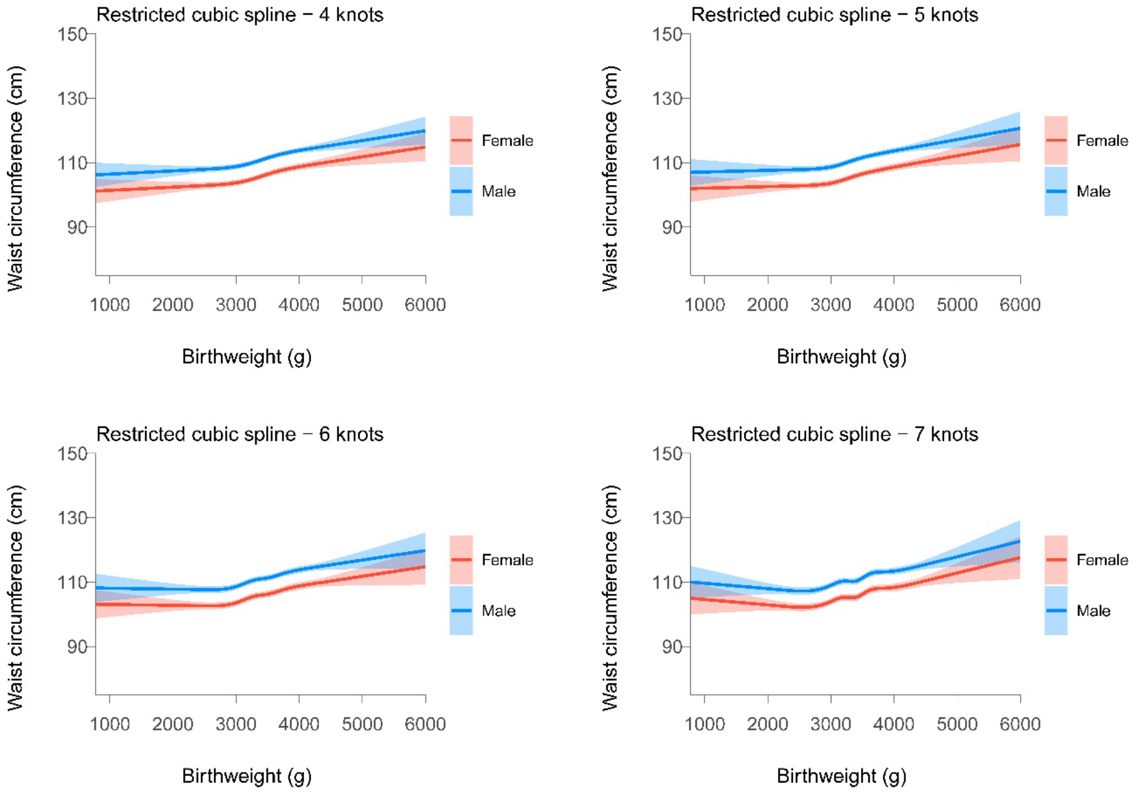

Multivariate imputations by chained equations (MICE) model specification

We employed multivariate imputations by chained equations using the MICE package from R[20].

The percentage of missing values across the dataset ranged from 0 to 57%, with an average of 13.75% missing values across all variables. Included below are plots showing the percentage of missing data per variable according to birthweight, age at enrolment, and sex. The plots show that the distributions of missing data according to birthweight categories, age at enrolment, and sex were similar, providing evidence against potential attrition bias.

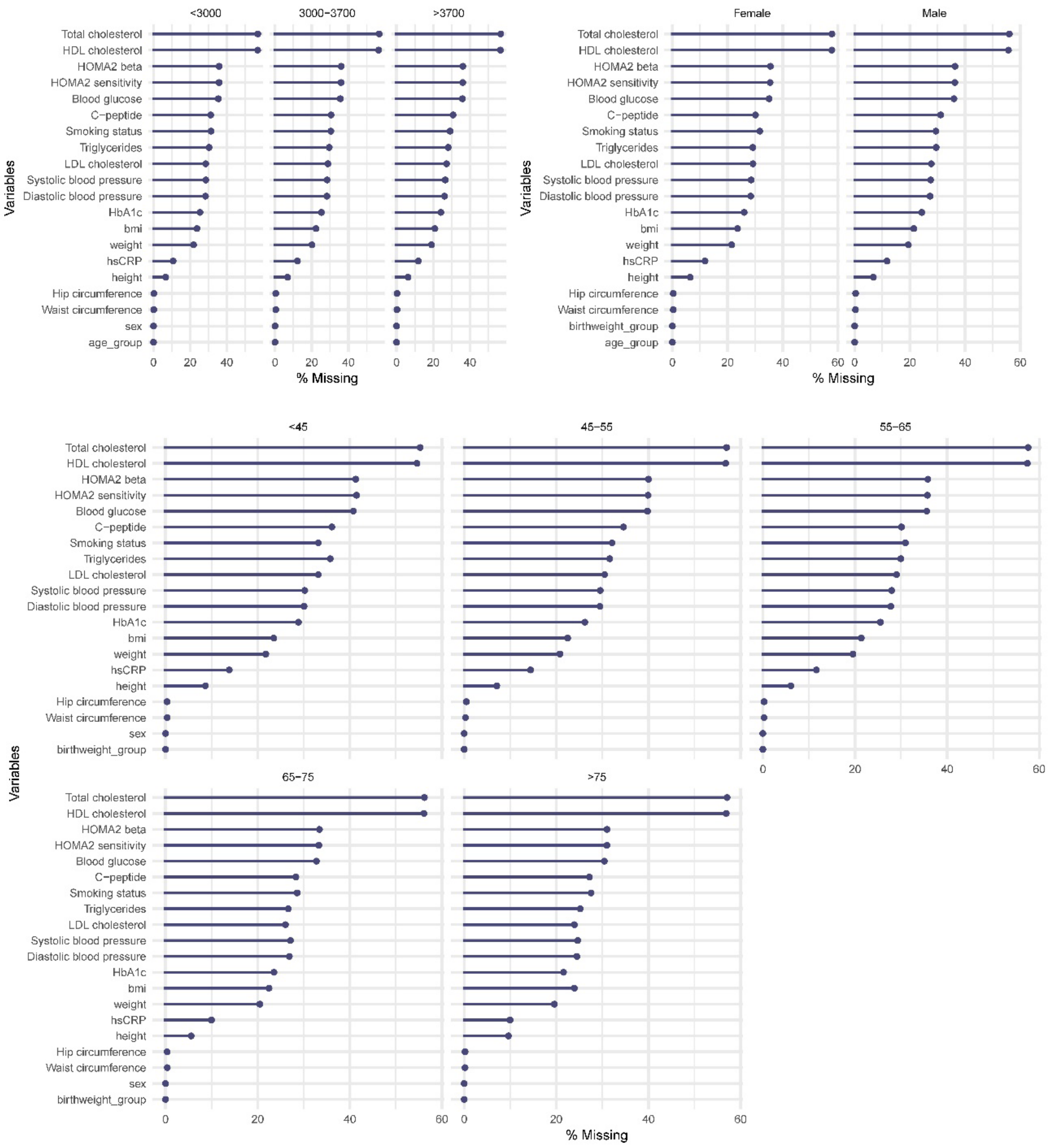

Legend: Percentage of missing data of variables used in the study. Each birthweight group is in kilograms. Abbreviations: BMI = Body Mass Index, HDL = high-density lipoprotein, LDL = low-density lipoprotein, hsCRP = high-sensitivity C-reactive protein, HOMA2 = Homeostasis Model Assessment 2, Log = natural logarithm, T2D = type 2 diabetes. Comorbidities and medication do not have any missing data.

We used multiple imputation to create and analyse 20 imputed datasets, with the number of iterations set to 10. Incomplete variables were imputed under fully conditional specification, using the default settings of the mice 3.14.0 package [20]. The parameters of substantive interest were estimated in each imputed dataset

separately and combined using Rubin's rules.
The following variables were used in the imputation model: age at enrolment, sex, place of enrolment, alcohol consumption, physical activity, weight, waist, height, BMI, waist–hip ratio, waist–height ratio, family history of type 2 diabetes, HOMA2-Beta, HOMA2 insulin sensitivity, C-peptide, blood glucose, hsCRP, birthweight, birth length, born-at-term status, total cholesterol, LDL cholesterol, HDL cholesterol, triglycerides, smoking status, systolic blood pressure, diastolic blood pressure, HbA_{1c}, number of glucose-lowering drugs, type of glucose-lowering drugs, lipid-lowering drugs, antihypertensive medication, number of antihypertensive drugs, Charlson Comorbidity Index Score, macrovascular complications, microvascular complications, diabetes-associated neurological disease, diabetes-associated eye disease, diabetes-associated renal disease.
The following variables were imputed by the following methods:
For continuous variables, predictive mean matching was used and included weight, waist, height, BMI, waist–hip ratio, waist–height ratio, HOMA2-Beta, HOMA2 insulin sensitivity, C-peptide, blood glucose, hsCRP, birth length, total cholesterol, HDL cholesterol, LDL cholesterol, triglycerides, systolic blood pressure, diastolic blood pressure, and HbA_{1c}.
For binary data logistic regression, imputation was used and included born-at-term status (only 49 individuals).
For categorical data with more than two levels, polytomous regression imputation was used and included smoking status.

We combined the raw data variables of weight, height, waist, and hip measurements into BMI, waist–hip ratio, and waist–height ratio before the imputation procedure. This procedure was justified by our findings that inclusion of only the raw variables for hip, weight, height, and waist gave rise to imprecise imputations, as can be seen below in the density plots. Specifically, hip circumference gave rise to imprecise imputations (bimodal density plots); therefore, it was not included in the imputation model as a single raw variable. Overall, the observed and imputed values had similar distributions, as shown in the density plots below.

Density plot of imputations – where BMI and waist–hip and waist–height ratios were calculated after imputation:

Red = Imputations, Blue = Original data.

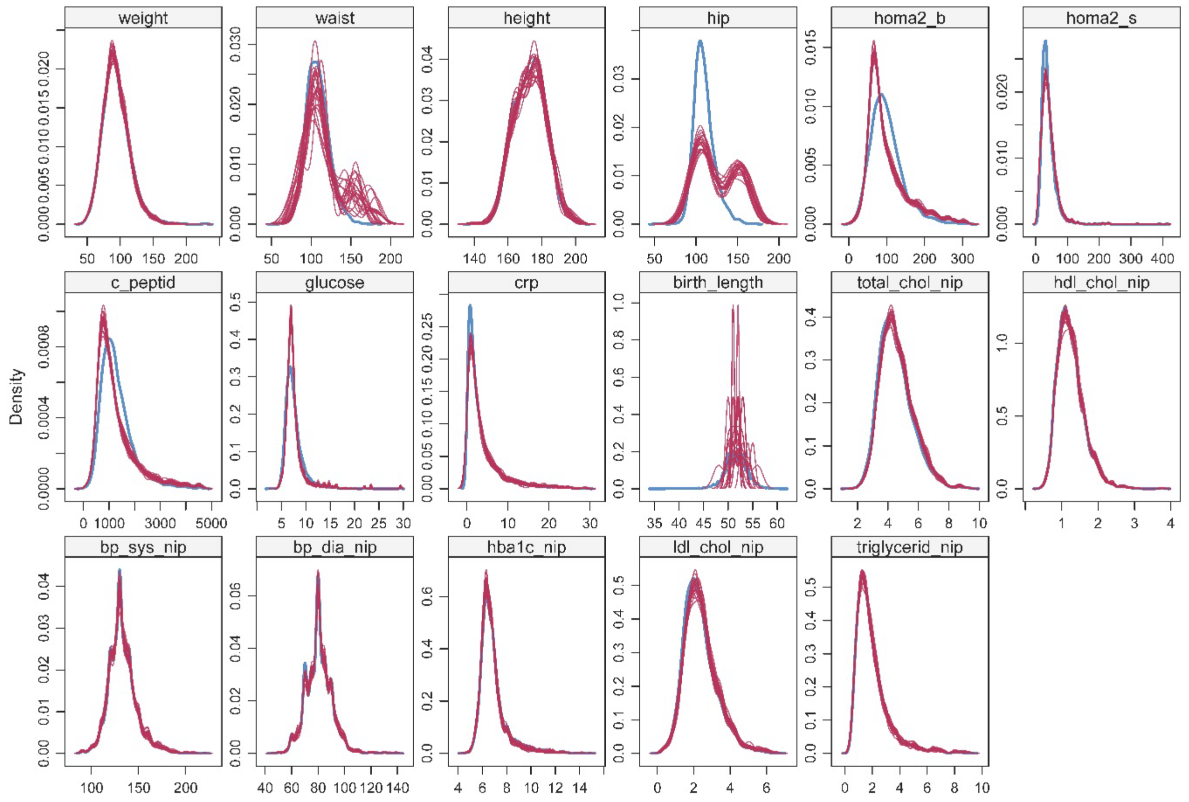

Variables in plot: weight; waist = waist circumference; height; hip = hip circumference; homa2_b = HOMA2 Beta; homa2_s = HOMA2-insulin sensitivity; c_peptid = C-peptide; glucose = blood glucose; crp = C-reactive protein; birth_length = birth length; total_chol_nip = total cholesterol; hdl_chol_nip = HDL cholesterol; bp_sys_nip = systolic blood pressure; bp_dia_np = diastolic blood pressure; hba1c_nip = HbA_{1c}; ldl_chol_nip = LDL cholesterol; triglycerid_nip = triglycerides

Density plot of imputations, where BMI and waist–hip and waist–height ratios were calculated before imputation and the raw hip measurement variable was excluded:

Red = imputations; blue = original data.

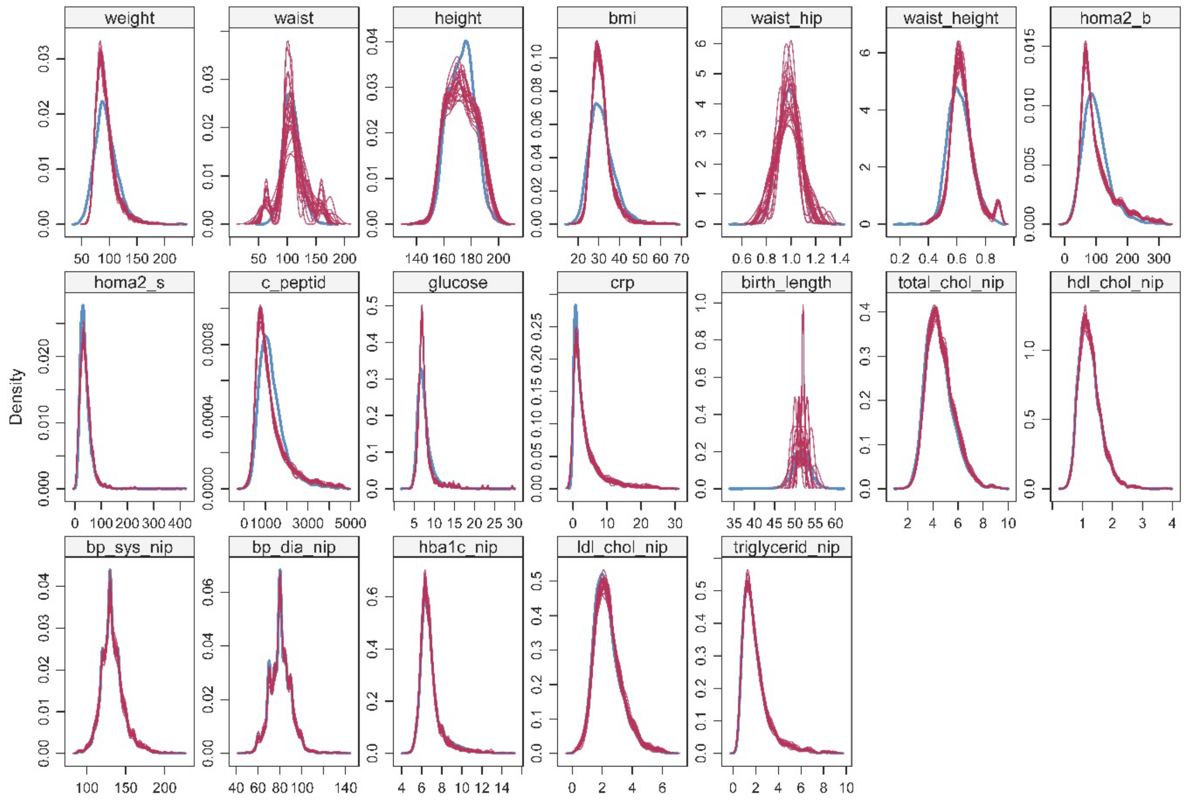

Variables in plot: weight, waist = waist circumference, height, bmi = body mass index, waist_hip = waist–hip ratio, waist_height = waist–height ratio, homa2_b = HOMA2 Beta, homa2_s = HOMA2-insulin sensitivity, c_peptid = C-peptide, glucose = blood glucose, crp = C-reactive protein, birth_length = birth length, total_chol_nip = total cholesterol, hdl_chol_nip = HDL cholesterol, bp_sys_nip = systolic blood pressure, bp_dia_np = diastolic blood pressure, hba1c_nip = HbA_{1c}, ldl_chol_nip = LDL cholesterol, triglycerid_nip = triglycerides

MICE	Min.	Q1	Median	Mean	Q3	Max.
**Waist–Hip Ratio**						
Complete cases	0.54	0.92	0.98	0.98	1.04	1.39
Computed before imputations	0.54	0.92	0.98	0.98	1.04	1.39
Computed after imputations	0.49	0.92	0.98	0.98	1.04	2.20
**Waist–Height Ratio**						
Complete cases	0.20	0.56	0.62	0.62	0.68	0.90
Computed before imputations	0.20	0.56	0.62	0.62	0.68	0.90
Computed after imputations	0.29	0.56	0.62	0.62	0.68	1.12
**BMI (kg/m^2)**						
Complete cases	16.65	27.32	30.76	31.50	34.87	66.33
Computed before imputations	16.65	27.47	30.61	31.39	34.34	66.33
Computed after imputations	13.13	27.27	30.73	31.48	34.89	75.10

Distribution of variables to be either computed before or after imputations.

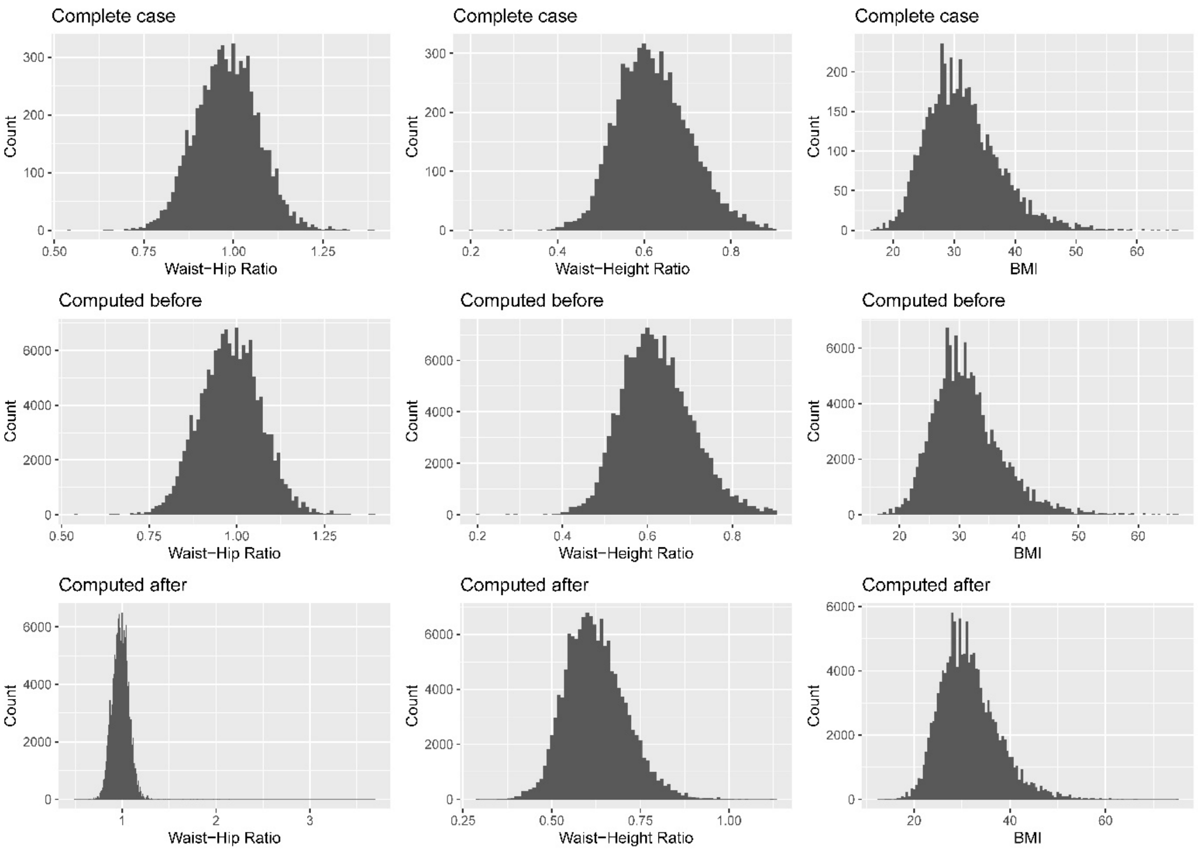
Complete case
Count
Waist−Hip Ratio
Complete case
Count
Waist−Height Ratio
Complete case
Count
BMI
Computed before
Count
Waist−Hip Ratio
Computed before
Count
Waist−Height Ratio
Computed before
Count
BMI
Computed after
Count
Waist−Hip Ratio
Computed after
Count
Waist−Height Ratio
Computed after
Count
BMI

ESM Tables

ESM Table 1. DD2 inclusion criteria, definitions, and codes.

**Inclusion criteria up to September 29, 2018**
1. During the entire DD2 enrolment period, the diagnosis of type 2 diabetes in routine clinical practice in Denmark has followed WHO criteria. Before 2012 this was primarily based on the oral glucose tolerance test (OGTT) or fasting plasma glucose measurements. Since 2012 it has primarily been based on glycosylated haemoglobin A (HbA_{1c}) >48 mmol/mol (6.5%). No further diagnostic criteria have been applied in the DD2 project. Moreover, patients are eligible whether or not they have initiated glucose-lowering therapy at the time of DD2 cohort enrolment.<br>Up to September 29, 2018, a "newly diagnosed" type 2 diabetes patient was principally defined in DD2 as a patient diagnosed later than 1 January 2009, with the recommendation to include only patients with a diabetes duration shorter than 1 year. Median diagnosed diabetes duration at enrolment in the total DD2 cohort is 1.3 years, interquartile range [IQR] 0.3–2.9 years.<br>2. Age ≥18 years<br>**3.** The patient can give informed consent.
**Inclusion criteria after September 29, 2018**
After September 29, 2018, the first criterion was changed to "A diagnosis of type 2 diabetes made within the last 24 months"; this is not relevant for our study period. The remaining 2 criteria are unchanged.
Recruitment was done at all Danish outpatient clinics and in approximately 462 general practitioner clinics (1853 clinics exists) through the courtesy of the clinicians at the recruiting sites.

**Definitions and codes used in this study**	
Variable	Definitions and codes
Biological sex	Male or female. Defined by social security number in registries.
Age at enrolment	Age at study enrolment in DD2<br>Continuous (years)
Age at type 2 diabetes diagnosis	Defined as age at whichever of the following events came first:<br>1. First prescription for glucose-lowering drugs<br>2. First diabetes-related diagnosis in the Danish National Patient Registry<br>3. DDDA registration<br>4. First measured HbA_{1c} >48 mmol/mol (6.5%)<br>5. DD2 enrolment<br>Continuous (years)<br>Categorical<br>• <45 years of age<br>• 45 to <55 years of age<br>• 55 to <65 years of age<br>• 65 to <75 years of age<br>• ≥75 years of age
Family history of type 2 diabetes	Self-reported at DD2 study enrolment. Includes any affected among mother, father, grandparents, and siblings<br>Categorical (total number)<br>• 0<br>• 1<br>• 2<br>• 3+
Enrolment status	Patients were enrolled at either general practices or hospital outpatient clinics

Weight at enrolment	In the few cases where weight recorded as part of the DD2 enrolment process was available (initially this was not part of the DD2 core data), we used that weight measure; otherwise the DDDA weight.<br>Weights below 35 and above 300 kg were considered outliers.
Height	Data on height is available from two different sources:<br>DD2 enrolment, DDDA data (repeated measures), DD2 Diabetic Neuropathy Questionnaire 2016 (self-reported).<br><br>Regarding DDDA data: a mean height based on all available DDDA heights was calculated for all patients 18 years or older.<br><br>We do not expect height to change over time among these adults. Thus, we used the available heights in a hierarchical order; height from DD2 enrolment (measured by health personnel, but very few available), DD2 questionnaire survey in 2016, and DDDA.<br>Heights below 130 cm and above 220 were considered outliers and not included in any calculations
Body mass index (BMI) at enrolment	BMI were calculated using the standard formula of weight/height (in meter)^2<br><br>Categories<br><br>• <25<br>• 25 to <30<br>• 30 to <35<br>• 35 to <40<br>• ≥40<br><br>BMI below 15 kg/m^2 and above 70 kg/m^2 were considered outliers and not included in any calculations
Waist circumference	Measured at DD2 enrolment. In cm<br>Categories:<br>Male/female:<br>• <94/80 cm<br>• 94 to <102/80 to <88 cm<br>• ≥102/88 cm<br>Reference:<br>WHO: World health Organ Tech Rep Ser 2000;894: i-xii, 1–253<br>IDF: Diabet Med 2006; 23:469-480<br><br>Waist circumferences below 50 cm and above 184 cm were considered outliers and not included in any calculations.
Hip circumference	Measured at DD2 enrolment. In cm.<br>Hip circumferences below 44 cm and above 175 cm were considered outliers and not included in any calculations.
Waist–hip ratio (WHR)	Waist and hip circumference were measured at DD2 enrolment. In cm. Categories according to lowest 25%, middle 50%, and highest 25%<br>• <0.92<br>• 0.92–1.04<br>• >1.04<br><br>References:<br>We based our categories on often-used waist–hip ratio classifications in the scientific literature together with observations of baseline WHR distributions in our cohort [1].<br>Waist–hip ratios below 0.5 and above 1.44 were considered outliers and not included in any calculations.
Waist–height ratio	Waist circumference measured at DD2 enrolment.<br>Height: See above.<br>Categories [2]:<br>• <0.5<br>• 0.5 to <0.6<br>• ≥ .6<br>Waist–height ratios above 0.9 were considered outliers and not included in any calculations.
Alcohol consumption	Self-reported at enrolment. Units/week<br>High risk intake: >14/21 drinks per week for female/male.

	High-risk alcohol consumption was categorized according to the Danish Health Authority's definitions as more than 21 and 14 drinks weekly for men and women, respectively, in 2010 when the DD2 was initiated.
Smoking Status	Information on smoking through DDDA data. Self-reported.<br>Categories:<br>• Never smoker<br>• Former smoker<br>• Current smoker (comprising daily and occasional use)
Physical activity	Physical activity – Self-reported at DD2 enrolment: “days per week of minimum 30 minutes of moderate to vigorous activity”<br>• 0<br>• 1–2<br>• 3–4<br>• 5–6<br>• 7 (all days in a week)
Metabolic profile	
Systolic blood pressure	Blood pressure in mmHg. Collected from DDDA, using the value obtained closest to the DD2 enrolment date.<br>Categories:<br>• <125<br>• 125–134<br>• 135–154<br>• ≥155<br>Based on hypertensive groups according to the Danish Society of Cardiology.<br>Systolic blood pressures below 90 mmHg and above 260 mmHg were considered outliers and not included in any calculations.
Diastolic blood pressure	Blood pressure in mmHg. Collected from DDDA, using the value obtained closest to DD2 enrolment date.<br>Categories:<br>• <80<br>• 80–84<br>• 85–94<br>• ≥95<br>Based on hypertensive groups according to the Danish Society of Cardiology.<br>Diastolic blood pressures below 30 mmHg and above 140 mmHg were considered outliers and not included in any calculations.
Hypertension	Hypertension, defined as systolic blood pressure ≥130 mmHg or diastolic blood pressure ≥80 mmHg, or antihypertensive medication usage, excluding loop diuretics.<br>Blood pressure information from DDDA. Antihypertensive medication usage from DNHSP. See below for ATC codes.
Total cholesterol	Collected from DDDA, using the value obtained closest to the DD2 enrolment date. In mmol/l<br>Categories according to lowest 25%, middle 50% and highest 25%<br>• < 3.70<br>• 3.70–5.10<br>• >5.10<br>Total cholesterol values above 9.87 mmol/l were considered outliers and not included in any calculations.
Triglycerides	Collected from DDDA, using the value obtained closest to the DD2 enrolment date. In mmol/l<br>Categories according to lowest 25 %, middle 50 % and highest 25 %<br>• <1.20<br>• 1.20–2.40<br>• >2.40<br>Triglycerides above 9.30 mmol/l were considered outliers and not included in any calculations.
HDL cholesterol	Collected from DDDA, using the value obtained closest to the DD2 enrolment date. In mmol/l<br>Categories (male/female)[1, 3]:<br>• <1.03/1.29<br>• ≥1.03/1.29<br>HDL cholesterol values above 3.08 mmol/l were considered outliers and not included in any calculations.
LDL cholesterol	Collected from DDDA, using the value closest to DD2 enrolment date. In mmol/l<br>Categories:<br>• <1.8<br>• 1.8 to < 2.6

	• ≥2.6<br>LDL cholesterol values above 7 mmol/l were considered outliers and not included in any calculations.
Dyslipidaemia	Collected from DDDA, using the value closest to the DD2 enrolment date and The Danish National Health Service Prescription Registry: Defined as LDL ≥2.6 mmol/l, or HDL <1.02 mmol/l, or triglycerides ≥1.7 mmol/l [1], or use of lipid-lowering drugs. Cholesterol cut off levels defined according to the American Diabetes Association [4]
Blood glucose	Fasting blood glucose in mmol/l. Measured at DD2 enrolment.<br>Categories:<br>• <6.5<br>• 6.5 to <7.0<br>• 7.0 to <7.5<br>• 7.5 to <9.0<br>• ≥9.0
HbA_{1c}	In mmol/mol (%). Collected from DDDA, using the value obtained closest to the enrolment date.<br>Categories:<br>• <48 (<6.5%)<br>• 48 to <53 (6.5 to <7.0%)<br>• 53 to <58 (7.0–7.5%)<br>• 58 - <75 (7.5–9.0%)<br>• ≥75 (≥9.0%)<br>HbA_{1c} values above 15% were considered outliers and not included in any calculations.
C-peptide	Measured at DD2 enrolment (pmol/l)<br>Categories:<br>• <850<br>• 850 to <1550<br>• ≥1550<br>C-peptide values above 4672 pmol/l were considered outliers and not included in any calculations. Categories according to previous publication [5]
hsCRP	High sensitivity low-grade inflammation: Excluding measures of hsCRP >30 mg/l in order to exclude values related to potential on-going infection. Measured at DD2 enrolment.<br>Categories:<br>• < 1.0<br>• 1.0–3.0<br>• ≥3.0<br>Categories according to previous publication[6]
HOMA2<br><br>- Insulin sensitivity<br>- Beta-cell function	We used the revised homeostatic assessment model version 2 (HOMA2) to estimate insulin sensitivity (HOMA2S) and the beta-cell function (HOMA2B) based on fasting C-peptide and plasma glucose values, based on the DD2 biobank [7].<br>Categories according to lowest 25%, middle 50% and highest 25%<br>HOMA2 Sensitivity<br>• <25.5<br>• 25.5–47<br>• >47<br>HOMA2 Beta<br>• <70<br>• 70–121<br>• >121<br>HOMA2 Sensitivity values above 512 and HOMA2 Beta above 320 were considered outliers and not included in any calculations.
HOMA2 based phenotypes	High and low values were defined as being above or below the median values for HOMA2-S and HOMA2-Beta in a non-diabetic background population selected from all residents (360,921) of one Danish County, as previously described [8]<br><br>Classical phenotype<br>• HOMA2B <115.3%<br>• HOMA2S <63.5%<br>Insulinopenic phenotype<br>• HOMA2B <115.3%<br>• HOMA2S ≥63.5%<br>Hypersinulinemic<br>• HOMA2B ≥115.3%<br>• HOMA2S <63.5%<br>Non-diabetes phenotype<br>• HOMA2B ≥115.3%

	• HOMA2S ≥63.5%
Medication usage (DNHSP)<br>*For all prescription data the relevant time period is around baseline = DD2 enrolment.*<br>*Thus, the look-back period is 1 year prior to the DD2 enrolment date*	
Antihypertensive drug usage	ATC:, C03A, C03B, C03D, C03E, C07, C08, C09A, C09B, C09C, C09D, C09X, G04CA03, C10BX04, C10BX06, C10BX07, C10BX11, C10BX12, C10BX13, C10BX14, C10BX15, C10BX10, C10BX07, C10BX09, C10BX11, C10BX14
Number of Antihypertensive drugs	One or more hits in one group counts as one agent: thiazides, potassium-sparing diuretics, beta-blockers, calcium channel antagonists, ACE inhibitors or ATII antagonists, renin inhibitors, alpha-blockers (Doxazosin), central adrenergic inhibition (Monoxidin, methyldopa).
Lipid-lowering drug usage	ATC: C10, A10BH51
Glucose-lowering drug usage	ATC: A10<br>Categories:<br>• No glucose-lowering drugs: no A10 prescription redemption<br>• Non-insulin only: ≥1 prescription redemption of A10B and NO prescription redemption of A10A<br>• Non-insulin + insulin or insulin only: ≥1 prescription redemption of A10B and ≥1 prescription redemption of A10A or ≥1 prescription redemption of A10A and NO prescription redemption of A10B
Number of different anti-diabetics including insulin	One hit or more hits in one group counts as one agent: sulfonylureas + meglitinides, metformin, alpha-glucosidase inhibitors, glitazones, DD4-inhibitors, GLP-1 analogues, SGLT-2 inhibitors, fast-acting insulin, long-acting insulin
Comorbidities and complications (DNPR)<br>*For all comorbidity and complications data the relevant time period is around baseline = DD2 enrolment.*<br>*Thus, the look-back period is 10 years prior to the DD2 enrolment date*	
Charlson Comorbidity Index Score<br>• Myocardial infarction<br>• Congestive heart failure<br>• Peripheral vascular disease<br>• Cerebrovascular disease<br>• Dementia<br>• Chronic pulmonary disease<br>• Connective tissue disease<br>• Ulcer disease<br>• Mild liver disease<br>• Hemiplegia<br>• Moderate to severe renal failure<br>• Any solid tumour<br>• Leukaemia<br>• Lymphoma<br>• Moderate to severe liver disease<br>• Metastatic solid tumour<br>• AIDS	Charlson comorbidity index score [9]. To assess the burden of comorbidities, the complete hospital contact history of each participant was obtained through linkage with the Danish National Patient Registry (DNPR), which contains discharge records from all Danish hospitalizations since 1977 and hospital outpatient visits since 1995, coded according to the International Classification of Diseases, 10th Edition [10]. We retrieved data on the 19 major comorbid disease categories included in the Charlson Comorbidity Index (CCI) [11, 12], and computed a CCI score for each person (excluding diabetes as it constituted the index disease of our study population). Linkage to the Danish National Patient Registry.
Any macrovascular complications	Type 2 diabetes with macrovascular complications was obtained through linkage with the Danish National Patient Registry. The following ICD-10 and procedure codes were used:<br>KFNA, KFNB, KFNC, KFND, KFNE, KFNF, KFNG, KFNH, KFNW, KFLF, KPAE, KPAF, KPAH, KPAN, KPAP, KPAW99, KPAU74, KPBE, KPBF, KPBH, KPBN, KPBP, KPBW, KPBQ, KPCE, KPCF, KPCH, KPCN, KPCP, KPCW99, KPCW20, KPCU74, KPDF, KPDH, KPDN, KPDP, KPDW, KPDQ, KPCU82, KPCU83, KPCU84, KPDE, KPDW99, KPDW20, KPEE, KPEF, KPEH, KPEN, KPEP, KPEW, KPEQ, KPFE, KPFH, KPFN, KPFP, KPFW, KPFQ, KPGH10, KPGH20, KPGH21, KPGH22, KPGH23, KPGH30, KPGH31, KPGH40, KPGH99, KPDU74, KPDU82, KPDU83, KPDU84, KPEU74, KPEU82, KPEU83, KPEU84, KPFU74, KPFU82, KPFU83, KPFU84, KPGU74, KPGU83, KPGU84, KPGU99, KPGW, KPWG, KAAL10, KAAL11, DI20, DI21, DI200, DT822A, DT823, DT822A, DT823, DG45, DI61, DI672, DI691, DI702, DI739A, DI739B, DI739C, DE105, DE115, DE125, DE135, DE145, KNBQ, KNCQ, KNDQ, KNEQ, KNFQ, KNGQ, KNHQ,

	DI700, DI708, DI709, DN280, DI701
Any microvascular complications	Type 2 diabetes with microvascular complications was obtained through linkage with the Danish National Patient Registry. The following ICD-10 codes were used:<br>DE104, DE114, DE124, DE134, DE144, DG590, DG632, DG603, DG609, DG618, DG619, DG620, DG621, DG622, DG628, DG629, DG630, DG631, DG632, DG634, DG635, DG636, DG638, DG990, DG561, DG562, DG563, DG568, DG569, DG570, DG572, DG573, DG574, DG576, DG578, DG579, DG580, DG587, DG588, DG589, DG598, DG900, DG560, DG575, DE103, DE113, DE123, DE133, DE143, DH280, DH334, DH450, DH360, DH25, DH268, DH269, DH430, DH431, DH438C, DH439, DH334, DH332, DH330, DH335, KCKC10, KCKC15, KCKD65, KCKD05B, DH360K, DH360J, DH540, DH541, DH544, DH545, DE102, DE112, DE122, DE132, DE142, DI120, DI131, DI132, DN083, DN06, DO084, DR809, BJFD2, BJFD0, DZ992
Diabetes-associated eye disease	Type 2 diabetes with eye-associated complications was obtained through linkage with the Danish National Patient Registry. The following ICD-10 codes were used:<br>DE103, DE113, DE123, DE133, DE143, DH280, DH334, DH450, DH360, DH25, DH268, DH269, DH430, DH431, DH438C, DH439, DH334, DH332, DH330, DH335, KCKC10, KCKC15, KCKD65, DH540, DH541, DH544, DH545, KCKD05B, DH360K, DH360J
Diabetes-associated kidney disease	Type 2 diabetes with kidney complications was obtained through linkage with the Danish National Patient Registry. The following ICD-10 codes were used:<br>DE102, DE112, DE122, DE132, DE142, DI120, DI131, DE132, DN083, DN06, DO084, DR809, BJFD2, BJFD0, DZ992
Diabetes-associated neurological disease	Type 2 diabetes with neurological complications was obtained through linkage with the Danish National Patient Registry. The following ICD-10 codes were used:<br>DE104, DE114, DE124, DE134, DE144, DG590, DG632, DG603, DG609, DG618, DG619, DG620, DG621, DG622, DG628, DG629, DG630, DG631, DG632, DG634, DG635, DG636, DG638, DG990, DG561, DG562, DG563, DG568, DG569, DG570, DG572, DG573, DG574, DG576, DG578, DG579, DG580, DG587, DG588, DG589, DG598, DG900, DG560, DG575, DG990

ESM Table 2: Hypertension, lipids and glucose homeostasis and subclinical inflammation

Metabolic profile	Birthweight (grams) n (%)			
	**<3000 (n=1675)**	**3000–3700 (n=3525)**	**>3700 (n=1666)**	**Total (n=6866)**
Systolic blood pressure (mmHg)				
median [iqr]	130 [125, 140]	130 [125, 140]	130 [124, 140]	130 [125, 140]
<125	361 (30.08)	755 (29.85)	402 (32.68)	1518 (30.61)
125–134	388 (32.33)	870 (34.40)	400 (32.52)	1658 (33.43)
135–154	358 (29.83)	755 (29.85)	333 (27.07)	1446 (29.16)
≥155	93 (7.75)	149 (5.89)	95 (7.72)	337 (6.80)
Diastolic blood pressure (mmHg)				
median [iqr]	80 [75, 86]	80 [75, 86]	80 [74, 85]	80 [75, 86]
<80	660 (55.00)	1443 (57.06)	736 (59.84)	2839 (57.25)
80–84	230 (19.17)	435 (17.20)	187 (15.20)	852 (17.18)
85–94	235 (19.58)	516 (20.40)	237 (19.27)	988 (19.92)
≥95	75 (6.25)	135 (5.34)	70 (5.69)	280 (5.65)
Hypertension				
No	73 (4.77)	169 (5.26)	84 (5.47)	326 (5.19)
Yes	1457 (95.23)	3042 (94.74)	1452 (94.53)	5951 (94.81)
Total Cholesterol (mmol/l)				
median [iqr]	4.4 [3.8, 5.2]	4.3 [3.8, 5.1]	4.2 [3.6, 4.9]	4.3 [3.7, 5.1]
<3.70	172 (23.79)	378 (24.79)	231 (32.08)	781 (26.31)
3.70–5.10	354 (48.96)	778 (51.02)	350 (48.61)	1482 (49.93)
>5.10	197 (27.25)	369 (24.20)	139 (19.31)	705 (23.75)
Triglycerides (mmol/l)				
median [iqr]	1.7 [1.2, 2.5]	1.7 [1.2, 2.4]	1.6 [1.2, 2.3]	1.7 [1.2, 2.4]
<1.20	325 (27.61)	711 (28.41)	359 (29.84)	1395 (28.57)
1.20–2.40	549 (46.64)	1198 (47.86)	577 (47.96)	2324 (47.59)
>2.40	303 (25.74)	594 (23.73)	267 (22.19)	1164 (23.84)
HDL Cholesterol (mmol/l)				
median [iqr]	1.2 [1.0, 1.5]	1.2 [1.0, 1.4]	1.2 [1.0, 1.4]	1.2 [1.0, 1.4]
<1.03/1.29 (male/female)	317 (43.78)	626 (40.81)	294 (40.66)	1237 (41.50)
≥1.03/1.29 (male/female)	407 (56.22)	908 (59.19)	429 (59.34)	1744 (58.50)
LDL Cholesterol (mmol/l)				
median [iqr]	2.2 [1.7, 2.9]	2.2 [1.7, 2.8]	2.1 [1.70, 2.73]	2.2 [1.7, 2.8]
<1.8	376 (31.31)	801 (31.90)	429 (35.40)	1606 (32.62)
1.8–2.6	439 (36.55)	961 (38.27)	448 (36.96)	1848 (37.53)
≥2.6	386 (32.14)	749 (29.83)	335 (27.64)	1470 (29.85)

Dyslipidaemia				
No	35 (2.37)	76 (2.45)	38 (2.60)	149 (2.47)
Yes	1444 (97.63)	3026 (97.55)	1425 (97.40)	5895 (97.53)
Blood glucose (mmol/l)				
median [iqr]	7.16 [6.37, 8.29]	7.18 [6.43, 8.23]	7.13 [6.36, 8.17]	7.15 [6.39, 8.24]
<6.5	321 (29.64)	618 (27.22)	326 (30.52)	1265 (28.61)
6.5–7.0	179 (16.53)	405 (17.84)	168 (15.73)	752 (17.01)
7.0–7.5	144 (13.30)	318 (14.01)	154 (14.42)	616 (13.93)
7.5–9.0	263 (24.28)	574 (25.29)	252 (23.60)	1089 (24.63)
≥9.0	176 (16.25)	355 (15.64)	168 (15.73)	699 (15.81)
HbA_{1c} (mmol/mol [%])				
median [iqr] mmol/mol	48.9 [44.7, 56.1]	48.9 [44.3, 55.0]	48.9 [44.7, 55.0]	48.9 [44.7, 55.0]
median [iqr] %	6.62 [6.24, 7.28]	6.62 [6.20, 7.18]	6.62 [6.24, 7.18]	6.62 [6.24, 7.18]
<48 (6.5%)	505 (40.34)	1150 (43.63)	558 (44.11)	2213 (42.95)
48–53 (6.5–7%)	340 (27.16)	719 (27.28)	334 (26.40)	1393 (27.03)
53–58 (7.0–7.5%)	161 (12.86)	294 (11.15)	144 (11.38)	599 (11.62)
58–75 (7.5–9.0%)	158 (12.62)	299 (11.34)	149 (11.78)	606 (11.76)
≥75 (9.0%)	88 (7.03)	174 (6.60)	80 (6.32)	342 (6.64)
C peptide (pmol/l)				
median [iqr]	1140 [872.15, 1558.50]	1176 [865.15, 1553.00]	1196 [889.6, 1632.0]	1171 [875.30, 1574.25]
<850	268 (23.20)	588 (23.90)	255 (22.04)	1111 (23.28)
850–1550	593 (51.34)	1256 (51.06)	572 (49.44)	2421 (50.73)
≥1550	294 (25.45)	616 (25.04)	330 (28.52)	1240 (25.98)
hsCRP (mg/l)				
median [iqr]	2.11 [0.91, 4.59]	1.97 [0.84, 4.41]	1.94 [0.84, 4.16]	2 [0.85, 4.40]
<1.0	409 (27.34)	925 (29.89)	434 (29.56)	1768 (29.18)
1.0–3.0	509 (34.02)	1061 (34.28)	508 (34.60)	2078 (34.30)
≥3.0	578 (38.64)	1109 (35.83)	526 (35.83)	2213 (36.52)
HOMA2 insulin sensitivity				
median [iqr]	35.2 [25.9, 47.1]	34.5 [25.8, 47.4]	33.5 [24.3, 46.0]	34.6 [25.5, 47.0]
<25.5	254 (23.58)	554 (24.53)	299 (28.08)	1107 (25.16)
25.5–47	552 (51.25)	1131 (50.09)	511 (47.98)	2194 (49.86)
>47	271 (25.16)	573 (25.38)	255 (23.94)	1099 (24.98)
HOMA2 Beta				
median [iqr]	92.3 [69.3, 118.5]	92.6 [70.1, 119.4]	96.4 [71.5, 125.8]	93.7 [70.20, 121.23]
<70	278 (25.81)	564 (24.98)	252 (23.66)	1094 (24.86)
70–121	549 (50.97)	1151 (50.97)	503 (47.23)	2203 (50.07)

>121	250 (23.21)	543 (24.05)	310 (29.11)	1103 (25.07)

Legend: Table of metabolic variables according to birthweight groups. Abbreviations: HDL = high-density lipoprotein, LDL = low-density lipoprotein, hsCRP = high-sensitivity C-reactive protein, HOMA2 = Homeostasis Model Assessment 2, iqr = interquantile range.

ESM Table 3: Medication usage

Medication	Birthweight (grams) n (%)			
	**<3000 (n=1675)**	**3000–3700 (n=3525)**	**>3700 (n=1666)**	**Total (n=6866)**
Use of antihypertensive drugs				
No	466 (27.82)	987 (28.00)	449 (26.95)	1902 (27.70)
Yes	1209 (72.18)	2538 (72.00)	1217 (73.05)	4964 (72.30)
Number of antihypertensive drugs				
0	466 (27.82)	987 (28.00)	449 (26.95)	1,902 (27.70)
1	402 (24.00)	753 (21.36)	363 (21.79)	1,518 (22.11)
2	378 (22.57)	890 (25.25)	406 (24.37)	1,674 (24.38)
3+	429 (25.61)	895 (25.39)	448 (26.89)	1,772 (25.81)
Use of lipid-lowering drugs				
No	486 (29.01)	1028 (29.16)	503 (30.19)	2017 (29.38)
Yes	1189 (70.99)	2497 (70.84)	1163 (69.81)	4849 (70.62)
Use of glucose-lowering drugs				
non-insulin only	1312 (78.33)	2771 (78.61)	1295 (77.73)	5378 (78.33)
no glucose-lowering drugs	241 (14.39)	539 (15.29)	268 (16.09)	1048 (15.26)
insulin and oral or insulin only	122 (7.28)	215 (6.10)	103 (6.18)	440 (6.41)
Number of glucose-lowering drugs				
0	241 (14.39)	539 (15.29)	268 (16.09)	1,048 (15.26)
1	1034 (61.73)	2257 (64.03)	1070 (64.23)	4,361 (63.52)
2	278 (16.60)	538 (15.26)	231 (13.87)	1,047 (15.25)
3+	122 (7.28)	191 (5.42)	97 (5.82)	410 (5.97)

Legend: Medication usage variables according to birthweight groups.

ESM Table 4: Comorbidities and diabetes-associated complications.

Comorbidities	**Birthweight (grams) n (%)**		
	**<3000 (n=1675)**	**3000–3700 (n=3525)**	**>3700 (n=1666)**
Charlson Comorbidity Index Score (CCI)			
0	1154 (68.90)	2419 (68.62)	1128 (67.71)
1–2	424 (25.31)	933 (26.47)	441 (26.47)
3+	97 (5.79)	173 (4.91)	97 (5.82)
Comorbidities used in CCI			
Myocardial infarction			
No	1593 (95.10)	3362 (95.38)	1580 (94.84)
Yes	82 (4.90)	163 (4.62)	86 (5.16)
Congestive heart failure			
No	1608 (96.00)	3393 (96.26)	1591 (95.50)
Yes	67 (4.00)	132 (3.74)	75 (4.50)
Peripheral vascular disease			
No	1610 (96.12)	3374 (95.72)	1590 (95.44)
Yes	65 (3.88)	151 (4.28)	76 (4.56)
Cerebrovascular disease			
No	1574 (93.97)	3290 (93.33)	1568 (94.12)
Yes	101 (6.03)	235 (6.67)	98 (5.88)
Dementia			
No	1672 (99.82)	3516 (99.74)	1662 (99.76)
Yes	<10	<10	<10
Chronic pulmonary disease			
No	1538 (91.82)	3260 (92.48)	1560 (93.64)
Yes	137 (8.18)	265 (7.52)	106 (6.36)
Connective tissue disease			
No	1639 (97.85)	3422 (97.08)	1624 (97.48)
Yes	36 (2.15)	103 (2.92)	42 (2.52)
Ulcer disease			
No	1648 (98.39)	3465 (98.30)	1633 (98.02)
Yes	27 (1.61)	60 (1.70)	33 (1.98)
Mild liver disease			
No	1647 (98.33)	3470 (98.44)	1644 (98.68)
Yes	28 (1.67)	55 (1.56)	22 (1.32)
Hemiplegia			
No	1672 (99.82)	3518 (99.80)	1662 (99.76)

Yes	<10	<10	<10
Moderate to severe renal disease			
No	1648 (98.39)	3470 (98.44)	1629 (97.78)
Yes	27 (1.61)	55 (1.56)	37 (2.22)
Any tumour			
No	1553 (92.72)	3279 (93.02)	1526 (91.60)
Yes	122 (7.28)	246 (6.98)	140 (8.40)
Leukaemia			
No	1672 (99.82)	3517 (99.77)	1662 (99.76)
Yes	<10	<10	<10
Lymphoma			
No	1668 (99.58)	3508 (99.52)	1659 (99.58)
Yes	<10	17 (0.48)	<10
Moderate to severe liver disease			
No	1672 (99.82)	3517 (99.77)	1661 (99.70)
Yes	<10	<10	<10
***Metastatic solid* tumour**			
No	1661 (99.16)	3506 (99.46)	1652 (99.16)
Yes	14 (0.84)	19 (0.54)	14 (0.84)
AIDS			
No	1673 (99.88)	3520 (99.86)	1666 (100.00)
Yes	<10	<10	<10
Diabetic macrovascular complications			
No	1382 (82.51)	2869 (81.39)	1344 (80.67)
Yes	293 (17.49)	656 (18.61)	322 (19.33)
Diabetic microvascular complications			
No	1520 (90.75)	3115 (88.37)	1481 (88.90)
Yes	155 (9.25)	410 (11.63)	185 (11.10)
Diabetes-associated eye disease			
No	1531 (91.40)	3187 (90.41)	1520 (91.24)
Yes	144 (8.60)	338 (9.59)	146 (8.76)
Diabetes-associated kidney disease			
No	1624 (96.96)	3423 (97.11)	1620 (97.24)
Yes	51 (3.04)	102 (2.89)	46 (2.76)
Diabetes-associated neurological disease			
No	1543 (92.12)	3161 (89.67)	1504 (90.28)
Yes	132 (7.88)	364 (10.33)	162 (9.72)

Legend: Comorbidities according to birthweight groups.

ESM Table 5: Stepwise adjustment in birth-weight categorical analysis

		Unadjusted	+ Sex	+ Age at enrolment	+ Family history of type 2 diabetes	+ BMI	+ lifestyle
Outcome	**Exposure**	**PR**	**aPR**	**aPR**	**aPR**	**aPR**	**aPR**
				Age at diagnosis (years)			
<45	<3000g	1.24 (1.07, 1.43)	1.25 (1.08, 1.45)	1.25, (1.08, 1.45)	1.28 (1.10, 1.48)	1.33 (1.15, 1.54)	1.33 (1.12, 1.58)
<45	>3700g	0.72 (0.60, 0.86)	0.71 (0.59, 0.85)	0.71, (0.59, 0.85)	0.69 (0.58, 0.83)	0.66 (0.55, 0.79)	0.65 (0.53, 0.81)
45–55	<3000g	1.29 (1.17, 1.42)	1.31 (1.19, 1.45)	1.31, (1.19, 1.45)	1.32 (1.20, 1.46)	1.34 (1.22, 1.48)	1.33 (1.20, 1.48)
45–55	>3700g	0.89 (0.79, 0.99)	0.88 (0.78, 0.98)	0.88, (0.78, 0.98)	0.87 (0.77, 0.97)	0.85 (0.76, 0.95)	0.85 (0.75, 0.95)
55–65	<3000g	0.94 (0.87, 1.02)	0.94 (0.87, 1.02)	0.94, (0.87, 1.02)	0.94 (0.87, 1.02)	0.94 (0.87, 1.02)	1.00 (0.92, 1.09)
55–65	>3700g	0.93 (0.85, 1.01)	0.93 (0.85, 1.01)	0.93, (0.85, 1.01)	0.93 (0.85, 1.01)	0.93 (0.86, 1.01)	0.85 (0.78, 0.93)
65–75	<3000g	0.77 (0.69, 0.86)	0.76 (0.68, 0.85)	0.76, (0.68, 0.85)	0.75 (0.67, 0.84)	0.73 (0.65, 0.82)	0.77 (0.70, 0.85)
65–75	>3700g	1.21 (1.10, 1.32)	1.22 (1.11, 1.34)	1.22, (1.11, 1.34)	1.24 (1.13, 1.36)	1.28 (1.17, 1.40)	1.24 (1.15, 1.34)
≥75	<3000g	0.73 (0.55, 0.97)	0.71 (0.53, 0.94)	0.71, (0.53, 0.94)	0.70 (0.53, 0.93)	0.67 (0.50, 0.89)	0.70 (0.56, 0.87)
≥75	>3700g	1.62 (1.31, 2.02)	1.65 (1.33, 2.05)	1.65, (1.33, 2.05)	1.71 (1.37, 2.12)	1.77 (1.43, 2.20)	1.54 (1.29, 1.84)
				Family history of type 2 diabetes (n)			
0	<3000g	1.00 (0.94, 1.06)	1.03 (0.98, 1.10)	1.07, (1.01, 1.14)	1.07 (1.01, 1.14)	1.08 (1.02, 1.14)	1.08 (1.02, 1.15)
0	>3700g	0.94 (0.88, 1.00)	0.92 (0.87, 0.98)	0.88, (0.83, 0.94)	0.88 (0.83, 0.94)	0.88 (0.83, 0.93)	0.88 (0.83, 0.94)
1	<3000g	1.02 (0.93, 1.11)	1.00 (0.92, 1.09)	0.99, (0.91, 1.08)	0.99 (0.91, 1.08)	0.98 (0.90, 1.07)	0.98 (0.90, 1.07)
1	>3700g	1.06 (0.98, 1.15)	1.07 (0.99, 1.17)	1.09, (1.00, 1.18)	1.09 (1.00, 1.18)	1.10 (1.01, 1.19)	1.09 (1.01, 1.19)
2	<3000g	1.04 (0.90, 1.20)	1.01 (0.87, 1.16)	0.96, (0.83, 1.10)	0.96 (0.83, 1.10)	0.96 (0.83, 1.10)	0.95 (0.83, 1.10)
2	>3700g	1.04 (0.91, 1.20)	1.07 (0.93, 1.23)	1.12, (0.97, 1.29)	1.12 (0.97, 1.29)	1.12 (0.98, 1.29)	1.11 (0.97, 1.28)
3+	<3000g	0.79 (0.61, 1.03)	0.74 (0.56, 0.96)	0.67, (0.52, 0.88)	0.67 (0.52, 0.88)	0.67 (0.51, 0.87)	0.67 (0.51, 0.88)
3+	>3700g	1.10 (0.87, 1.39)	1.16 (0.92, 1.47)	1.25, (0.99, 1.58)	1.25 (0.99, 1.58)	1.26 (1.00, 1.60)	1.27 (1.01, 1.61)
				Height (cm)			
<166	<3000g	1.48 (1.36, 1.61)	1.19 (1.12, 1.27)	1.26, (1.18, 1.35)	1.26 (1.18, 1.36)	1.26 (1.18, 1.36)	1.26 (1.18, 1.35)

<166	>3700g	0.73 (0.65, 0.82)	0.87 (0.79, 0.96)	0.84, (0.76, 0.92)	0.84 (0.76, 0.92)	0.84 (0.76, 0.92)	0.84 (0.76, 0.92)
166–180	<3000g	0.93 (0.87, 0.98)	1.01 (0.95, 1.06)	1.02, (0.96, 1.08)	1.02 (0.96, 1.08)	1.02 (0.96, 1.08)	1.02 (0.96, 1.08)
166–180	>3700g	0.92 (0.87, 0.98)	0.88 (0.83, 0.93)	0.87, (0.82, 0.92)	0.87 (0.82, 0.92)	0.87 (0.82, 0.92)	0.87 (0.82, 0.92)
>180	<3000g	0.60 (0.52, 0.70)	0.72 (0.63, 0.83)	0.68, (0.59, 0.78)	0.67 (0.59, 0.77)	0.67 (0.59, 0.77)	0.67 (0.58, 0.77)
>180	>3700g	1.53 (1.38, 1.69)	1.36 (1.24, 1.49)	1.46, (1.33, 1.59)	1.46 (1.34, 1.60)	1.46 (1.34, 1.60)	1.46 (1.33, 1.60)
	Weight (kg)						
<80	<3000g	1.38 (1.25, 1.52)	1.22 (1.12, 1.34)	1.35, (1.23, 1.48)	1.36 (1.24, 1.49)	1.36 (1.24, 1.49)	1.35 (1.23, 1.48)
<80	>3700g	0.67 (0.59, 0.77)	0.73 (0.64, 0.83)	0.67, (0.59, 0.76)	0.67 (0.59, 0.76)	0.67 (0.59, 0.76)	0.66 (0.59, 0.75)
80–106	<3000g	0.96 (0.90, 1.02)	0.98 (0.92, 1.04)	0.98, (0.92, 1.04)	0.98 (0.92, 1.04)	0.98 (0.92, 1.04)	0.98 (0.92, 1.04)
80–106	>3700g	1.02 (0.96, 1.08)	1.00 (0.94, 1.06)	1.00, (0.94, 1.06)	1.00 (0.94, 1.06)	1.00 (0.94, 1.06)	0.99 (0.94, 1.06)
>106	<3000g	0.69 (0.61, 0.79)	0.75 (0.66, 0.85)	0.68, (0.60, 0.78)	0.68 (0.60, 0.78)	0.68 (0.60, 0.78)	0.69 (0.61, 0.78)
>106	>3700g	1.31 (1.19, 1.44)	1.24 (1.13, 1.37)	1.35, (1.23, 1.49)	1.36 (1.24, 1.50)	1.36 (1.24, 1.50)	1.36 (1.24, 1.50)
	BMI (kg/m2)						
<25	<3000g	1.11 (1.02, 1.20)	1.09 (1.01, 1.19)	1.12, (1.04, 1.22)	1.12 (1.03, 1.22)	1.12 (1.03, 1.22)	1.12 (1.03, 1.22)
<25	>3700g	0.91 (0.83, 0.99)	0.91 (0.83, 1.00)	0.90, (0.82, 0.98)	0.90 (0.82, 0.98)	0.90 (0.82, 0.98)	0.90 (0.82, 0.98)
25–30	<3000g	1.03 (0.94, 1.14)	1.07 (0.97, 1.17)	1.10, (1.00, 1.22)	1.11 (1.01, 1.22)	1.11 (1.01, 1.22)	1.10 (1.00, 1.21)
25–30	>3700g	0.90 (0.81, 1.00)	0.88 (0.79, 0.98)	0.85, (0.77, 0.94)	0.84 (0.76, 0.93)	0.84 (0.76, 0.93)	0.84 (0.76, 0.94)
30–35	<3000g	0.97 (0.87, 1.08)	0.98 (0.88, 1.09)	0.98, (0.88, 1.09)	0.98 (0.88, 1.09)	0.98 (0.88, 1.09)	0.98 (0.88, 1.09)
30–35	>3700g	1.12 (1.01, 1.24)	1.12 (1.01, 1.24)	1.12, (1.01, 1.24)	1.12 (1.01, 1.24)	1.12 (1.01, 1.24)	1.12 (1.01, 1.24)
35–40	<3000g	0.90 (0.76, 1.06)	0.88 (0.75, 1.04)	0.84, (0.71, 0.99)	0.84 (0.71, 0.99)	0.84 (0.71, 0.99)	0.84 (0.71, 0.98)
35–40	>3700g	1.19 (1.02, 1.37)	1.20 (1.04, 1.39)	1.25, (1.08, 1.45)	1.26 (1.09, 1.46)	1.26 (1.09, 1.46)	1.28 (1.10, 1.48)
≥40	<3000g	0.67 (0.53, 0.86)	0.64 (0.50, 0.81)	0.57, (0.45, 0.73)	0.57 (0.45, 0.72)	0.57 (0.45, 0.72)	0.57 (0.45, 0.73)
≥40	>3700g	1.08 (0.88, 1.32)	1.12 (0.92, 1.38)	1.27, (1.04, 1.54)	1.28 (1.05, 1.56)	1.28 (1.05, 1.56)	1.24 (1.02, 1.52)
	Waist circumference (male/female in cm)						
<94/80	<3000g	1.26 (1.07, 1.48)	1.28 (1.08, 1.50)	1.33, (1.13, 1.57)	1.34 (1.14, 1.58)	1.34 (1.14, 1.58)	1.33 (1.13, 1.57)

<94/80	>3700g	0.74 (0.61, 0.90)	0.73 (0.60, 0.90)	0.71, (0.58, 0.86)	0.70 (0.57, 0.85)	0.70 (0.57, 0.85)	0.70 (0.57, 0.86)
94–102/80–88	<3000g	1.02 (0.88, 1.18)	1.16 (1.01, 1.34)	1.21, (1.05, 1.39)	1.21 (1.05, 1.40)	1.21 (1.05, 1.40)	1.20 (1.04, 1.38)
94–102/80–88	>3700g	0.85 (0.73, 1.00)	0.78 (0.67, 0.91)	0.76, (0.65, 0.88)	0.75 (0.65, 0.88)	0.75 (0.65, 0.88)	0.76 (0.65, 0.88)
≥102/88	<3000g	0.96 (0.93, 1.00)	0.94 (0.91, 0.97)	0.93, (0.90, 0.96)	0.93 (0.90, 0.96)	0.93 (0.90, 0.96)	0.93 (0.90, 0.96)
≥102/88	>3700g	1.06 (1.03, 1.09)	1.08 (1.05, 1.11)	1.09, (1.06, 1.12)	1.09 (1.06, 1.12)	1.09 (1.06, 1.12)	1.09 (1.06, 1.12)
	Waist–hip ratio						
<0.92	<3000g	1.24 (1.13, 1.37)	1.04 (0.96, 1.13)	1.07, (0.98, 1.16)	1.07 (0.98, 1.16)	1.07 (0.98, 1.16)	1.06 (0.97, 1.15)
<0.92	>3700g	0.87 (0.78, 0.97)	1.01 (0.92, 1.12)	1.00, (0.91, 1.10)	1.00 (0.91, 1.10)	1.00 (0.91, 1.10)	1.01 (0.91, 1.11)
0.92–1.04	<3000g	0.99 (0.93, 1.04)	1.02 (0.96, 1.08)	1.02, (0.96, 1.08)	1.02 (0.96, 1.08)	1.02 (0.96, 1.08)	1.01 (0.96, 1.07)
0.92–1.04	>3700g	1.02 (0.97, 1.08)	1.01 (0.95, 1.06)	1.00, (0.95, 1.06)	1.00 (0.95, 1.06)	1.00 (0.95, 1.06)	1.00 (0.95, 1.06)
>1.04	<3000g	0.80 (0.72, 0.89)	0.93 (0.84, 1.03)	0.91, (0.82, 1.01)	0.91 (0.82, 1.01)	0.91 (0.82, 1.01)	0.91 (0.82, 1.01)
>1.04	>3700g	1.07 (0.97, 1.18)	0.98 (0.89, 1.07)	1.00, (0.91, 1.09)	0.99 (0.91, 1.09)	0.99 (0.91, 1.09)	1.00 (0.91, 1.09)
	Waist–height ratio						
<0.5	<3000g	1.09 (0.87, 1.37)	1.04 (0.83, 1.31)	1.05, (0.84, 1.32)	1.05 (0.84, 1.32)	1.05 (0.84, 1.32)	1.04 (0.83, 1.30)
<0.5	>3700g	0.76 (0.58, 0.98)	0.79 (0.61, 1.02)	0.78, (0.60, 1.01)	0.78 (0.60, 1.02)	0.78 (0.60, 1.02)	0.79 (0.61, 1.02)
0.5–0.6	<3000g	1.10 (1.02, 1.19)	1.13 (1.05, 1.22)	1.16, (1.07, 1.25)	1.16 (1.08, 1.25)	1.16 (1.08, 1.25)	1.15 (1.07, 1.24)
0.5–0.6	>3700g	0.98 (0.91, 1.06)	0.96 (0.89, 1.04)	0.94, (0.87, 1.02)	0.94 (0.86, 1.01)	0.94 (0.86, 1.01)	0.94 (0.87, 1.02)
≥0.6	<3000g	0.93 (0.88, 0.98)	0.92 (0.87, 0.97)	0.91, (0.86, 0.96)	0.90 (0.86, 0.95)	0.90 (0.86, 0.95)	0.91 (0.86, 0.96)
≥0.6	>3700g	1.04 (0.99, 1.09)	1.04 (0.99, 1.10)	1.06, (1.01, 1.11)	1.06 (1.01, 1.11)	1.06 (1.01, 1.11)	1.06 (1.01, 1.11)
	Systolic blood pressure (mmHg)						
<125	<3000g	1.01 (0.91, 1.12)	0.99 (0.89, 1.09)	0.96, (0.87, 1.07)	0.96 (0.87, 1.07)	0.94 (0.85, 1.05)	0.94 (0.85, 1.04)
<125	>3700g	1.06 (0.96, 1.16)	1.08 (0.98, 1.19)	1.10, (1.00, 1.21)	1.10 (1.00, 1.21)	1.12 (1.02, 1.23)	1.11 (1.01, 1.22)
125–134	<3000g	0.96 (0.88, 1.06)	0.97 (0.88, 1.07)	0.97, (0.88, 1.07)	0.97 (0.88, 1.07)	0.97 (0.88, 1.07)	0.97 (0.88, 1.07)
125–134	>3700g	0.96 (0.87, 1.07)	0.96 (0.87, 1.06)	0.96, (0.87, 1.06)	0.96 (0.86, 1.06)	0.96 (0.87, 1.06)	0.96 (0.87, 1.06)
135–154	<3000g	0.99 (0.90, 1.10)	1.01 (0.91, 1.12)	1.02, (0.92, 1.13)	1.02 (0.92, 1.13)	1.04 (0.93, 1.15)	1.04 (0.94, 1.15)

135–154	>3700g	0.93 (0.84, 1.03)	0.92 (0.83, 1.02)	0.91, (0.82, 1.01)	0.91 (0.82, 1.01)	0.90 (0.81, 1.00)	0.90 (0.81, 1.00)
≥155	<3000g	1.18 (0.93, 1.50)	1.21 (0.95, 1.53)	1.25, (0.99, 1.59)	1.26 (0.99, 1.59)	1.29 (1.02, 1.63)	1.29 (1.02, 1.64)
≥155	>3700g	1.24 (0.97, 1.59)	1.22 (0.95, 1.56)	1.18, (0.92, 1.51)	1.18 (0.92, 1.52)	1.15 (0.90, 1.48)	1.15 (0.90, 1.49)
	Diastolic blood pressure (mmHg)						
<80	<3000g	0.96 (0.91, 1.03)	0.95 (0.90, 1.01)	0.99, (0.93, 1.05)	0.99 (0.93, 1.05)	0.98 (0.92, 1.05)	0.98 (0.92, 1.04)
<80	>3700g	1.05 (0.99, 1.11)	1.05 (1.00, 1.12)	1.02, (0.96, 1.08)	1.02 (0.96, 1.08)	1.03 (0.97, 1.09)	1.02 (0.97, 1.08)
80–84	<3000g	1.09 (0.95, 1.25)	1.11 (0.96, 1.27)	1.07, (0.93, 1.24)	1.08 (0.93, 1.24)	1.08 (0.93, 1.24)	1.07 (0.93, 1.24)
80–84	>3700g	0.91 (0.79, 1.06)	0.90 (0.78, 1.05)	0.93, (0.80, 1.08)	0.93 (0.80, 1.08)	0.93 (0.80, 1.08)	0.93 (0.80, 1.08)
85–94	<3000g	0.98 (0.86, 1.12)	1.00 (0.87, 1.14)	0.94, (0.82, 1.08)	0.94 (0.82, 1.08)	0.95 (0.83, 1.09)	0.96 (0.83, 1.10)
85–94	>3700g	0.94 (0.82, 1.07)	0.93 (0.81, 1.06)	0.98, (0.86, 1.11)	0.98 (0.86, 1.11)	0.96 (0.84, 1.10)	0.97 (0.85, 1.10)
≥95	<3000g	1.15 (0.87, 1.51)	1.19 (0.90, 1.57)	1.09, (0.83, 1.44)	1.09 (0.82, 1.44)	1.12 (0.85, 1.49)	1.13 (0.85, 1.49)
≥95	>3700g	1.03 (0.78, 1.36)	1.01 (0.76, 1.32)	1.08, (0.82, 1.42)	1.09 (0.82, 1.43)	1.07 (0.81, 1.41)	1.07 (0.81, 1.41)
	Hypertension						
Yes	<3000g	1.01 (0.98, 1.03)	1.01 (0.99, 1.03)	1.02, (1.00, 1.05)	1.02 (1.00, 1.05)	1.03 (1.01, 1.06)	1.03 (1.01, 1.06)
Yes	>3700g	1.01 (0.99, 1.03)	1.01 (0.99, 1.03)	1.00, (0.97, 1.02)	1.00 (0.97, 1.02)	0.99 (0.97, 1.01)	0.99 (0.97, 1.01)
	Total cholesterol (mmol/l)						
<3.70	<3000g	0.91 (0.80, 1.04)	0.96 (0.84, 1.10)	0.98, (0.86, 1.12)	0.98 (0.86, 1.12)	0.99 (0.86, 1.13)	0.98 (0.85, 1.12)
<3.70	>3700g	1.23 (1.08, 1.39)	1.18 (1.05, 1.34)	1.17, (1.03, 1.32)	1.16 (1.03, 1.32)	1.16 (1.02, 1.31)	1.15 (1.02, 1.30)
3.70–5.10	<3000g	0.99 (0.93, 1.06)	0.98 (0.92, 1.05)	0.99, (0.92, 1.05)	0.99 (0.92, 1.05)	0.98 (0.92, 1.05)	0.98 (0.92, 1.05)
3.70–5.10	>3700g	0.97 (0.90, 1.05)	0.98 (0.91, 1.05)	0.98, (0.90, 1.05)	0.98 (0.90, 1.05)	0.98 (0.91, 1.06)	0.98 (0.91, 1.05)
>5.10	<3000g	1.09 (0.98, 1.22)	1.05 (0.95, 1.17)	1.04, (0.93, 1.16)	1.04 (0.93, 1.16)	1.04 (0.93, 1.16)	1.04 (0.94, 1.16)
>5.10	>3700g	0.85 (0.75, 0.97)	0.88 (0.77, 1.00)	0.89, (0.78, 1.01)	0.89 (0.78, 1.01)	0.89 (0.78, 1.01)	0.89 (0.79, 1.02)
	Triglycerides (mmol/l)						
<1.20	<3000g	0.96 (0.87, 1.07)	0.96 (0.87, 1.06)	1.00, (0.90, 1.11)	1.00 (0.91, 1.11)	0.96 (0.86, 1.06)	0.96 (0.87, 1.06)
<1.20	>3700g	1.06 (0.96, 1.17)	1.06 (0.96, 1.17)	1.01, (0.92, 1.12)	1.01 (0.91, 1.12)	1.06 (0.96, 1.18)	1.07 (0.97, 1.18)

1.20–2.40	<3000g	0.98 (0.91, 1.05)	0.97 (0.90, 1.04)	0.97, (0.91, 1.04)	0.97 (0.91, 1.04)	0.98 (0.92, 1.05)	0.98 (0.92, 1.05)
1.20–2.40	>3700g	1.00 (0.94, 1.07)	1.01 (0.94, 1.08)	1.00, (0.94, 1.07)	1.01 (0.94, 1.08)	1.00 (0.93, 1.07)	1.00 (0.93, 1.07)
>2.40	<3000g	1.10 (0.98, 1.23)	1.11 (0.99, 1.25)	1.05, (0.94, 1.18)	1.05 (0.94, 1.18)	1.09 (0.97, 1.22)	1.09 (0.97, 1.22)
>2.40	>3700g	0.92 (0.82, 1.04)	0.91 (0.81, 1.03)	0.96, (0.86, 1.08)	0.96 (0.86, 1.08)	0.93 (0.83, 1.05)	0.92 (0.82, 1.04)
	HDL cholesterol (mmol/l)						
≥1.03/1.29	<3000g	0.94 (0.85, 1.04)	0.95 (0.86, 1.05)	0.99, (0.89, 1.09)	0.99 (0.89, 1.09)	0.97 (0.87, 1.07)	0.97 (0.88, 1.08)
≥1.03/1.29	>3700g	1.00 (0.91, 1.10)	1.00 (0.90, 1.10)	0.96, (0.87, 1.06)	0.96 (0.87, 1.06)	0.98 (0.89, 1.08)	0.99 (0.89, 1.09)
	LDL cholesterol (mmol/l)						
<1.8	<3000g	0.97 (0.88, 1.07)	0.99 (0.89, 1.09)	1.01, (0.92, 1.12)	1.02 (0.92, 1.12)	1.02 (0.92, 1.12)	1.02 (0.92, 1.12)
<1.8	>3700g	1.08 (0.99, 1.19)	1.07 (0.97, 1.17)	1.04, (0.95, 1.14)	1.03 (0.94, 1.14)	1.03 (0.94, 1.14)	1.04 (0.95, 1.14)
1.8–2.6	<3000g	0.96 (0.89, 1.05)	0.96 (0.89, 1.05)	0.97, (0.89, 1.05)	0.97 (0.89, 1.05)	0.96 (0.89, 1.05)	0.96 (0.89, 1.05)
1.8–2.6	>3700g	0.98 (0.90, 1.06)	0.98 (0.90, 1.06)	0.97, (0.90, 1.06)	0.98 (0.90, 1.06)	0.98 (0.90, 1.06)	0.98 (0.90, 1.06)
≥2.6	<3000g	1.08 (0.98, 1.18)	1.06 (0.96, 1.16)	1.03, (0.94, 1.12)	1.02 (0.94, 1.12)	1.03 (0.94, 1.12)	1.03 (0.94, 1.12)
≥2.6	>3700g	0.94 (0.85, 1.04)	0.96 (0.86, 1.06)	0.98, (0.89, 1.09)	0.98 (0.89, 1.09)	0.98 (0.89, 1.09)	0.98 (0.89, 1.09)
	Dyslipidaemia						
Yes	<3000g	1.00 (0.98, 1.03)	1.00 (0.98, 1.03)	1.01, (0.99, 1.04)	1.01 (0.99, 1.04)	1.01 (0.99, 1.04)	1.01 (0.99, 1.04)
Yes	>3700g	1.00 (0.97, 1.02)	1.00 (0.97, 1.02)	0.99, (0.97, 1.01)	0.99 (0.97, 1.01)	0.99 (0.96, 1.01)	0.99 (0.96, 1.01)
	Blood glucose (mmol/l)						
<6.5	<3000g	1.06 (0.95, 1.18)	1.04 (0.93, 1.16)	1.07, (0.96, 1.19)	1.07 (0.96, 1.19)	1.06 (0.95, 1.18)	1.06 (0.95, 1.18)
<6.5	>3700g	1.11 (0.99, 1.24)	1.13 (1.01, 1.27)	1.10, (0.98, 1.24)	1.10 (0.98, 1.24)	1.12 (1.00, 1.25)	1.11 (0.99, 1.24)
6.5–7.0	<3000g	0.96 (0.83, 1.10)	0.94 (0.82, 1.09)	0.96, (0.84, 1.11)	0.96 (0.83, 1.11)	0.96 (0.83, 1.11)	0.96 (0.83, 1.10)
6.5–7.0	>3700g	0.96 (0.84, 1.10)	0.97 (0.85, 1.12)	0.95, (0.83, 1.09)	0.95 (0.83, 1.09)	0.96 (0.83, 1.10)	0.95 (0.83, 1.09)
7.0–7.5	<3000g	0.95 (0.81, 1.12)	0.96 (0.81, 1.13)	0.96, (0.81, 1.13)	0.96 (0.82, 1.14)	0.96 (0.81, 1.13)	0.96 (0.82, 1.13)
7.0–7.5	>3700g	1.02 (0.87, 1.20)	1.01 (0.87, 1.19)	1.01, (0.86, 1.18)	1.01 (0.86, 1.18)	1.02 (0.87, 1.19)	1.02 (0.87, 1.19)
7.5–9.0	<3000g	0.98 (0.87, 1.11)	1.00 (0.88, 1.13)	0.99, (0.87, 1.12)	0.99 (0.87, 1.12)	1.00 (0.88, 1.12)	1.00 (0.88, 1.13)

7.5–9.0	>3700g	0.91 (0.80, 1.04)	0.90 (0.79, 1.03)	0.91, (0.80, 1.04)	0.91 (0.80, 1.04)	0.90 (0.79, 1.03)	0.90 (0.79, 1.03)
≥9.0	<3000g	1.03 (0.88, 1.20)	1.07 (0.91, 1.25)	0.99, (0.85, 1.16)	1.00 (0.85, 1.17)	1.01 (0.87, 1.18)	1.02 (0.87, 1.19)
≥9.0	>3700g	0.98 (0.84, 1.16)	0.95 (0.81, 1.12)	1.02, (0.87, 1.19)	1.02 (0.87, 1.20)	1.01 (0.86, 1.19)	1.02 (0.87, 1.20)
	HbA_{1c} (mmol/mol)						
<48	<3000g	0.93 (0.86, 1.00)	0.92 (0.85, 0.99)	0.94, (0.87, 1.01)	0.94 (0.87, 1.01)	0.93 (0.86, 1.00)	0.93 (0.87, 1.01)
<48	>3700g	1.02 (0.96, 1.10)	1.03 (0.96, 1.11)	1.01, (0.94, 1.08)	1.01 (0.94, 1.09)	1.02 (0.96, 1.10)	1.03 (0.96, 1.10)
48–53	<3000g	1.00 (0.91, 1.11)	0.99 (0.89, 1.09)	1.01, (0.91, 1.11)	1.01 (0.91, 1.11)	1.01 (0.91, 1.12)	1.01 (0.91, 1.11)
48–53	>3700g	0.98 (0.88, 1.08)	0.98 (0.89, 1.09)	0.97, (0.88, 1.08)	0.97 (0.88, 1.08)	0.97 (0.87, 1.07)	0.96 (0.87, 1.07)
53–58	<3000g	1.13 (0.95, 1.35)	1.16 (0.97, 1.38)	1.14, (0.95, 1.36)	1.15 (0.96, 1.37)	1.16 (0.97, 1.39)	1.16 (0.97, 1.39)
53–58	>3700g	0.99 (0.83, 1.20)	0.98 (0.81, 1.18)	0.99, (0.83, 1.20)	0.99 (0.82, 1.19)	0.98 (0.81, 1.18)	0.98 (0.81, 1.18)
58–75	<3000g	1.10 (0.92, 1.32)	1.13 (0.94, 1.35)	1.05, (0.88, 1.26)	1.06 (0.89, 1.27)	1.09 (0.91, 1.31)	1.09 (0.91, 1.30)
58–75	>3700g	1.01 (0.84, 1.21)	0.99 (0.83, 1.19)	1.05, (0.88, 1.26)	1.05 (0.87, 1.26)	1.02 (0.85, 1.23)	1.01 (0.84, 1.22)
≥75	<3000g	1.07 (0.84, 1.36)	1.12 (0.88, 1.43)	1.01, (0.80, 1.28)	1.00 (0.79, 1.28)	1.03 (0.81, 1.31)	1.03 (0.81, 1.32)
≥75	>3700g	0.93 (0.72, 1.20)	0.90 (0.69, 1.16)	0.98, (0.76, 1.27)	0.99 (0.77, 1.28)	0.98 (0.76, 1.26)	0.98 (0.76, 1.26)
	C-peptide (pmol/l)						
<850	<3000g	0.99 (0.89, 1.10)	0.99 (0.89, 1.10)	1.01, (0.91, 1.12)	1.02 (0.91, 1.13)	0.91 (0.83, 1.01)	0.92 (0.83, 1.01)
<850	>3700g	0.98 (0.88, 1.09)	0.98 (0.88, 1.09)	0.97, (0.87, 1.08)	0.96 (0.86, 1.07)	1.07 (0.97, 1.19)	1.07 (0.97, 1.19)
850–1550	<3000g	1.01 (0.94, 1.08)	1.00 (0.94, 1.08)	1.00, (0.94, 1.08)	1.01 (0.94, 1.08)	1.02 (0.95, 1.09)	1.02 (0.95, 1.09)
850–1550	>3700g	0.96 (0.89, 1.02)	0.96 (0.89, 1.03)	0.96, (0.89, 1.03)	0.95 (0.89, 1.02)	0.94 (0.88, 1.01)	0.94 (0.88, 1.01)
≥1550	<3000g	1.00 (0.88, 1.13)	1.00 (0.89, 1.13)	0.98, (0.87, 1.11)	0.98 (0.87, 1.10)	1.06 (0.94, 1.19)	1.06 (0.94, 1.19)
≥1550	>3700g	1.11 (1.00, 1.23)	1.10 (0.99, 1.23)	1.13, (1.01, 1.25)	1.14 (1.02, 1.27)	1.06 (0.95, 1.18)	1.06 (0.95, 1.17)
	HOMA2 insulin sensitivity						
<25.5	<3000g	0.98 (0.87, 1.09)	0.98 (0.88, 1.10)	0.96, (0.86, 1.08)	0.95 (0.85, 1.07)	1.03 (0.92, 1.15)	1.03 (0.92, 1.15)
<25.5	>3700g	1.07 (0.96, 1.20)	1.07 (0.95, 1.20)	1.09, (0.98, 1.23)	1.10 (0.99, 1.24)	1.03 (0.92, 1.15)	1.02 (0.92, 1.14)
25.5–47	<3000g	1.02 (0.95, 1.09)	1.01 (0.95, 1.08)	1.01, (0.95, 1.08)	1.02 (0.95, 1.09)	1.02 (0.96, 1.10)	1.03 (0.96, 1.10)

25.5–47	>3700g	0.95 (0.88, 1.02)	0.95 (0.88, 1.02)	0.95, (0.88, 1.02)	0.95 (0.88, 1.02)	0.94 (0.87, 1.01)	0.94 (0.87, 1.01)
>47	<3000g	0.99 (0.89, 1.11)	0.99 (0.89, 1.11)	1.01, (0.91, 1.14)	1.02 (0.91, 1.14)	0.92 (0.83, 1.02)	0.92 (0.83, 1.03)
>47	>3700g	1.03 (0.93, 1.14)	1.03 (0.93, 1.14)	1.01, (0.91, 1.12)	1.00 (0.90, 1.11)	1.12 (1.01, 1.23)	1.11 (1.01, 1.23)
				HOMA2-beta			
<70	<3000g	1.03 (0.93, 1.14)	1.06 (0.96, 1.17)	1.03, (0.93, 1.13)	1.03 (0.94, 1.14)	0.97 (0.88, 1.07)	0.98 (0.89, 1.08)
<70	>3700g	0.96 (0.87, 1.07)	0.95 (0.85, 1.05)	0.97, (0.88, 1.08)	0.97 (0.87, 1.07)	1.03 (0.93, 1.14)	1.03 (0.93, 1.14)
70–121	<3000g	1.00 (0.93, 1.08)	0.99 (0.92, 1.07)	1.00, (0.93, 1.09)	1.01 (0.93, 1.09)	1.01 (0.94, 1.09)	1.01 (0.94, 1.09)
70–121	>3700g	0.96 (0.89, 1.03)	0.96 (0.90, 1.04)	0.95, (0.88, 1.02)	0.95 (0.88, 1.02)	0.95 (0.88, 1.02)	0.95 (0.88, 1.02)
>121	<3000g	0.96 (0.85, 1.08)	0.95 (0.84, 1.07)	0.96, (0.84, 1.08)	0.95 (0.84, 1.07)	1.01 (0.90, 1.14)	1.01 (0.89, 1.14)
>121	>3700g	1.13 (1.01, 1.26)	1.14 (1.02, 1.27)	1.13, (1.01, 1.26)	1.15 (1.03, 1.28)	1.08 (0.97, 1.20)	1.07 (0.96, 1.19)
				hsCRP (mg/l)			
<1.0	<3000g	0.92 (0.84, 1.02)	0.96 (0.87, 1.06)	0.99, (0.90, 1.09)	0.99 (0.90, 1.09)	0.92 (0.84, 1.01)	0.92 (0.84, 1.01)
<1.0	>3700g	1.00 (0.90, 1.10)	0.97 (0.88, 1.07)	0.94, (0.86, 1.04)	0.94 (0.85, 1.03)	1.02 (0.92, 1.12)	1.02 (0.92, 1.12)
1.0–3.0	<3000g	0.99 (0.91, 1.08)	1.00 (0.91, 1.09)	1.01, (0.92, 1.10)	1.01 (0.92, 1.10)	1.00 (0.92, 1.09)	1.00 (0.92, 1.09)
1.0–3.0	>3700g	1.01 (0.92, 1.10)	1.00 (0.92, 1.09)	0.99, (0.91, 1.08)	0.99 (0.91, 1.08)	1.00 (0.91, 1.09)	1.00 (0.91, 1.08)
>3.0	<3000g	1.07 (0.99, 1.16)	1.04 (0.96, 1.12)	1.01, (0.93, 1.09)	1.00 (0.93, 1.08)	1.06 (0.98, 1.15)	1.06 (0.99, 1.15)
>3.0	>3700g	1.00 (0.92, 1.08)	1.02 (0.94, 1.11)	1.05, (0.97, 1.13)	1.06 (0.98, 1.15)	1.00 (0.93, 1.08)	1.00 (0.92, 1.08)
				Antihypertensive drug usage			
Yes	<3000g	1.00 (0.97, 1.04)	1.00 (0.97, 1.04)	1.04, (1.00, 1.08)	1.04 (1.00, 1.08)	1.06 (1.02, 1.09)	1.06 (1.02, 1.10)
Yes	>3700g	1.01 (0.98, 1.05)	1.01 (0.98, 1.05)	0.98, (0.95, 1.01)	0.98 (0.95, 1.02)	0.97 (0.93, 1.00)	0.97 (0.94, 1.00)
0	<3000g	0.99 (0.90, 1.09)	0.99 (0.90, 1.09)	0.91, (0.83, 1.00)	0.91 (0.83, 1.00)	0.88 (0.80, 0.96)	0.85 (0.77, 0.95)
0	>3700g	0.96 (0.88, 1.06)	0.96 (0.87, 1.06)	1.04, (0.95, 1.14)	1.04 (0.95, 1.14)	1.09 (0.99, 1.19)	1.09 (0.98, 1.21)
1	<3000g	1.12 (1.01, 1.25)	1.12 (1.01, 1.25)	1.12, (1.00, 1.24)	1.12 (1.01, 1.25)	1.10 (0.99, 1.23)	1.07 (0.95, 1.21)
1	>3700g	1.02 (0.91, 1.14)	1.02 (0.91, 1.14)	1.03, (0.92, 1.15)	1.02 (0.91, 1.14)	1.04 (0.93, 1.16)	0.99 (0.87, 1.13)
2	<3000g	0.89 (0.80, 0.99)	0.89 (0.80, 0.99)	0.92, (0.83, 1.02)	0.92 (0.83, 1.02)	0.94 (0.84, 1.04)	0.99 (0.88, 1.12)

2	>3700g	0.97 (0.87, 1.07)	0.97 (0.88, 1.07)	0.94, (0.85, 1.04)	0.94 (0.85, 1.04)	0.92 (0.83, 1.02)	0.97 (0.87, 1.09)
3+	<3000g	1.01 (0.91, 1.11)	1.02 (0.92, 1.12)	1.10, (0.99, 1.21)	1.09 (0.99, 1.20)	1.14 (1.03, 1.25)	1.11 (0.99, 1.24)
3+	>3700g	1.06 (0.96, 1.17)	1.05 (0.96, 1.16)	0.98, (0.89, 1.08)	0.99 (0.90, 1.09)	0.95 (0.86, 1.04)	0.93 (0.84, 1.04)
	Glucose-lowering drug usage						
None	<3000g	0.94 (0.82, 1.08)	0.93 (0.81, 1.07)	0.97, (0.84, 1.12)	0.98 (0.85, 1.12)	0.96 (0.84, 1.11)	0.97 (0.84, 1.11)
None	>3700g	1.05 (0.92, 1.20)	1.06 (0.93, 1.21)	1.01, (0.89, 1.16)	1.01 (0.88, 1.16)	1.03 (0.90, 1.18)	1.03 (0.90, 1.18)
Oral	<3000g	1.00 (0.97, 1.03)	0.99 (0.96, 1.02)	0.99, (0.96, 1.02)	0.99 (0.96, 1.02)	0.99 (0.96, 1.02)	0.99 (0.96, 1.02)
Oral	>3700g	0.99 (0.96, 1.02)	0.99 (0.96, 1.02)	0.99, (0.96, 1.02)	0.99 (0.96, 1.02)	0.99 (0.96, 1.02)	0.99 (0.96, 1.02)
Insulin + Oral	<3000g	1.19 (0.96, 1.48)	1.25 (1.01, 1.55)	1.18, (0.95, 1.46)	1.17 (0.94, 1.45)	1.17 (0.94, 1.45)	1.17 (0.95, 1.45)
Insulin + Oral	>3700g	1.01 (0.81, 1.27)	0.98 (0.78, 1.23)	1.04, (0.83, 1.31)	1.05 (0.84, 1.32)	1.05 (0.84, 1.32)	1.04 (0.83, 1.31)
0	<3000g	0.94 (0.82, 1.08)	0.93 (0.81, 1.07)	0.97, (0.85, 1.12)	0.98 (0.85, 1.12)	0.96 (0.84, 1.11)	0.92 (0.77, 1.09)
0	>3700g	1.05 (0.92, 1.20)	1.06 (0.93, 1.21)	1.02, (0.89, 1.16)	1.01 (0.89, 1.16)	1.03 (0.90, 1.18)	1.00 (0.84, 1.17)
1	<3000g	0.96 (0.92, 1.01)	0.96 (0.91, 1.00)	0.96, (0.92, 1.01)	0.96 (0.92, 1.01)	0.96 (0.92, 1.01)	0.95 (0.90, 1.01)
1	>3700g	1.00 (0.96, 1.05)	1.01 (0.97, 1.05)	1.01, (0.96, 1.05)	1.01 (0.96, 1.05)	1.01 (0.96, 1.05)	1.00 (0.95, 1.05)
2	<3000g	1.09 (0.95, 1.24)	1.11 (0.98, 1.27)	1.07, (0.93, 1.22)	1.07 (0.94, 1.22)	1.08 (0.94, 1.23)	1.15 (0.99, 1.33)
2	>3700g	0.91 (0.79, 1.05)	0.89 (0.78, 1.03)	0.93, (0.81, 1.07)	0.93 (0.81, 1.07)	0.93 (0.80, 1.07)	0.96 (0.82, 1.13)
3+	<3000g	1.34 (1.08, 1.67)	1.41 (1.13, 1.76)	1.32, (1.06, 1.65)	1.33 (1.06, 1.65)	1.34 (1.08, 1.68)	1.29 (1.02, 1.63)
3+	>3700g	1.07 (0.85, 1.36)	1.04 (0.82, 1.32)	1.11, (0.88, 1.41)	1.11 (0.87, 1.40)	1.09 (0.86, 1.38)	1.07 (0.84, 1.36)
	Lipid-lowering drug usage						
Yes	<3000g	1.00 (0.97, 1.04)	1.00 (0.97, 1.04)	1.02, (0.99, 1.06)	1.02 (0.99, 1.06)	1.02 (0.99, 1.06)	1.03 (0.99, 1.06)
Yes	>3700g	0.99 (0.95, 1.02)	0.99 (0.95, 1.02)	0.97, (0.93, 1.00)	0.97 (0.93, 1.00)	0.96 (0.93, 1.00)	0.96 (0.93, 1.00)
	Charlson Comorbidity Index score						
0	<3000g	1.00 (0.97, 1.04)	1.00 (0.96, 1.04)	0.97, (0.94, 1.01)	0.97 (0.94, 1.01)	0.97 (0.93, 1.01)	0.97 (0.93, 1.01)
0	>3700g	0.99 (0.95, 1.03)	0.99 (0.95, 1.03)	1.02, (0.98, 1.06)	1.01 (0.97, 1.05)	1.02 (0.98, 1.06)	1.02 (0.98, 1.06)
1–2	<3000g	0.96 (0.87, 1.06)	0.96 (0.87, 1.06)	1.01, (0.92, 1.12)	1.01 (0.91, 1.11)	1.02 (0.93, 1.13)	1.03 (0.93, 1.13)

1–2	>3700g	1.00 (0.91, 1.10)	1.00 (0.90, 1.10)	0.95, (0.87, 1.05)	0.96 (0.87, 1.06)	0.94 (0.85, 1.03)	0.93 (0.84, 1.02)
3+	<3000g	1.18 (0.93, 1.50)	1.21 (0.95, 1.54)	1.37, (1.08, 1.74)	1.36 (1.07, 1.73)	1.36 (1.07, 1.72)	1.36 (1.07, 1.73)
3+	>3700g	1.19 (0.93, 1.51)	1.17 (0.92, 1.49)	1.02, (0.80, 1.30)	1.04 (0.82, 1.32)	1.04 (0.82, 1.32)	1.05 (0.83, 1.34)
	Macrovascular complications						
Yes	<3000g	0.94 (0.83, 1.06)	0.99 (0.87, 1.12)	1.08, (0.95, 1.22)	1.08 (0.95, 1.22)	1.08 (0.96, 1.23)	1.08 (0.96, 1.22)
Yes	>3700g	1.04 (0.92, 1.17)	1.00 (0.89, 1.13)	0.92, (0.82, 1.04)	0.92 (0.82, 1.04)	0.92 (0.81, 1.03)	0.91 (0.81, 1.03)
	Microvascular complications						
Yes	<3000g	0.80 (0.67, 0.95)	0.78 (0.66, 0.94)	0.79, (0.66, 0.94)	0.79 (0.66, 0.94)	0.80 (0.67, 0.95)	0.80 (0.67, 0.95)
Yes	>3700g	0.95 (0.81, 1.12)	0.96 (0.82, 1.14)	0.96, (0.81, 1.13)	0.96 (0.82, 1.14)	0.95 (0.81, 1.12)	0.95 (0.81, 1.12)
	Diabetes-associated eye disease						
Yes	<3000g	0.90 (0.74, 1.08)	0.89 (0.73, 1.07)	0.99, (0.82, 1.19)	0.97 (0.81, 1.17)	0.97 (0.81, 1.17)	0.97 (0.80, 1.16)
Yes	>3700g	0.91 (0.76, 1.10)	0.92 (0.77, 1.11)	0.83, (0.69, 1.00)	0.84 (0.70, 1.01)	0.85 (0.70, 1.02)	0.85 (0.71, 1.02)
	Diabetes-associated kidney disease						
Yes	<3000g	1.05 (0.76, 1.47)	1.12 (0.80, 1.56)	1.18, (0.85, 1.65)	1.18 (0.84, 1.65)	1.22 (0.88, 1.71)	1.22 (0.87, 1.70)
Yes	>3700g	0.95 (0.68, 1.34)	0.91 (0.65, 1.29)	0.87, (0.61, 1.23)	0.87 (0.62, 1.24)	0.84 (0.59, 1.20)	0.84 (0.59, 1.20)
	Diabetes-associated neurological disease						
Yes	<3000g	0.76 (0.63, 0.92)	0.75 (0.62, 0.90)	0.74, (0.61, 0.90)	0.74 (0.61, 0.90)	0.75 (0.62, 0.91)	0.75 (0.62, 0.90)
Yes	>3700g	0.94 (0.79, 1.12)	0.96 (0.80, 1.14)	0.97, (0.81, 1.15)	0.97 (0.81, 1.15)	0.96 (0.80, 1.14)	0.95 (0.80, 1.14)

Legend: Stepwise prevalence ratio adjustment. Lifestyle model = + alcohol, smoking status, physical activity. Abbreviations: BMI = body mass index, BP = blood pressure, HDL = high density lipoprotein, LDL = low density lipoprotein, hsCRP = high-sensitivity C-reactive protein, HOMA2 = Homeostasis Model Assessment 2, PR = prevalence ratio.

ESM Table 6: Stepwise adjustment for linear regression

	Adjustment level	Coefficient (95% CI)	P-value	R^2 (%)
Age at type 2 diabetes diagnosis (years)		3.06 (2.58, 3.53)	<0.0001	2.26
	Sex	3.17 (2.70, 3.65)	<0.0001	2.45
	+ Family history of type 2 diabetes	3.33 (2.86, 3.80)	<0.0001	6.41
	+ BMI	3.79 (3.33, 4.24)	<0.0001	13.56
	+ Alcohol, smoking status, physical activity	3.68 (3.23, 4.13)	<0.0001	17.03
Height (cm)		3.97 (3.55, 4.38)	<0.0001	5.10
	Sex	2.39 (2.07, 2.71)	<0.0001	47.28
	+ Family history of type 2 diabetes	3.00 (2.69, 3.31)	<0.0001	52.17
	+ Age at enrolment	3.02 (2.71, 3.33)	<0.0001	52.22
	+ Alcohol, smoking status, physical activity	3.02 (2.71, 3.33)	<0.0001	52.27
Weight (kg)		3.35 (2.99, 3.71)	<0.0001	52.31
	Sex	6.59 (5.68, 7.49)	<0.0001	3.12
	+ Family history of type 2 diabetes	5.17 (4.28, 6.053)	<0.0001	10.69
	+ Age at enrolment	7.115 (6.265, 7.97)	<0.0001	21.62
	+ Alcohol, smoking status, physical activity	7.20 (6.35, 8.06)	<0.0001	21.75
BMI (kg/m²)		0.83 (0.56, 1.10)	<0.0001	0.54
	Sex	0.94 (0.67, 1.213	<0.0001	1.04
	+ Family history of type 2 diabetes	1.43 (1.16, 1.70)	<0.0001	8.59
	+ Age at enrolment	1.46 (1.19, 1.73)	<0.0001	8.75
	+ Alcohol, smoking status, physical activity	1.47 (1.20, 1.73)	<0.0001	11.79
Waist circumference (cm)		3.63 (2.98, 4.28)	<0.0001	1.72
	Sex	2.93 (2.29, 3.58)	<0.0001	5.03

	+ Family history of type 2 diabetes	3.80 (3.16, 4.44)	<0.0001	8.95
	+ Age at enrolment	3.90 (3.26, 4.54)	<0.0001	9.19
	+ Alcohol, smoking status, physical activity	3.88 (3.25, 4.51)	<0.0001	12.70
Waist–hip ratio		0.01 (0.01, 0.02)	<0.0001	0.74
	Sex	0.002 (−0.001, 0.006)	0.1549	27.09
	+ Family history of type 2 diabetes	0.004 (0.001, 0.007)	0.0145	27.56
	+ Age at enrolment	0.004 (0.001, 0.008)	0.0127	27.57
	+ Alcohol, smoking status, physical activity	0.004 (0.001, 0.007)	0.0179	28.77
Waist–height ratio		0.007 (0.003, 0.011)	0.0002	0.20
	Sex	0.009 (0.005, 0.012)	<0.0001	0.82
	+ Family history of type 2 diabetes	0.011 (0.008, 0.015)	<0.0001	2.15
	+ Age at enrolment	0.012 (0.008, 0.016)	<0.0001	2.38
	+ Alcohol, smoking status, physical activity	0.012 (0.008, 0.015)	<0.0001	6.39
Systolic blood pressure (mmHg)		0.17 (−0.62, 0.96)	0.6669	0.01
	Sex	-0.01 (−0.88, 0.68)	0.8029	0.55
	+ Family history of type 2 diabetes	-0.39 (−1.17, 0.38)	0.3182	1.04
	+ Age at enrolment	-0.37 (−1.15, 0.40)	0.345	1.15
	+ BMI	-0.65 (−1.43, 0.13)	0.1040	1.74
	+ Alcohol, smoking status, physical activity	-0.35 (−1.13, 0.42)	0.3685	1.49
Diastolic blood pressure (mmHg)		-0.54 (−1.03, −0.05)	0.0325	0.09
	Sex	−0.75 (−1.24, −0.26)	0.0030	0.79
	+ Family history of type 2 diabetes	−0.04 (−0.52, 0.44)	0.8735	6.98
	+ Age at enrolment	−0.02 (−0.50, 0.46)	0.9224	7.07
	+ BMI	−0.22 (−0.70, 0.25)	0.3607	7.73
	+ Alcohol, smoking status, physical activity	−0.01 (−0.49, 0.47)	0.9766	7.30
Total cholesterol (mmol/l)		−0.12 (−0.18, −0.07)	<0.0001	0.38
	Sex	−0.09 (−0.14, −0.03)	0.0043	2.37
	+ Family history of type 2 diabetes	−0.07 (−0.12, −0.01)	0.0222	2.71
	+ Age at enrolment	−0.07 (−0.12, −0.01)	0.0239	2.76
	+ BMI	−0.06 (−0.12, −0.01)	0.0265	2.78

	+ Alcohol, smoking status, physical activity	−0.06 (−0.12, −0.01)	0.0280	3.45
Triglycerides (log) % change		−4.28 (−6.78, −1.71)	0.0013	0.20
	Sex	−4.66 (−7.16, −2.09)	0.0005	0.29
	+ Family history of type 2 diabetes	−1.83 (−4.38, 0.79)	0.1689	3.82
	+ Age at enrolment	−1.79 (−4.34, 0.82)	0.1772	3.89
	+ BMI	−4.29 (−6.72, −1.79)	0.0009	7.60
	+ Alcohol, smoking status, physical activity	−4.37 (−6.80, −1.88)	0.0007	8.96
HDL (log) % change		−1.34 (−3.08, 0.44)	0.1380	0.07
	Sex	0.51 (−1.25, 2.30)	0.5660	6.69
	+ Family history of type 2 diabetes	−1.56 (−3.35, 0.26)	0.0908	13.08
	+ Age at enrolment	−1.49 (−3.30, 0.35)	0.1100	13.17
	+ BMI	−0.16 (−1.98, 1.70)	0.8669	16.82
	+ Alcohol, smoking status, physical activity	−0.05 (−1.88, 1.81)	0.9551	20.96
HbA_{1c} (log) % change		−0.89 (−1.61, −0.17)	0.0163	0.10
	Sex	−1.15 (−1.87, −0.42)	0.0020	0.56
	+ Family history of type 2 diabetes	−0.39 (−1.11, 0.34)	0.2928	3.52
	+ Age at enrolment	−0.424 (−1.149, 0.307)	0.2545	3.68
	+ BMI	−0.69 (−1.41, 0.03)	0.0637	4.17
	+ Alcohol, smoking status, physical activity	−0.68 (−1.41, 0.04)	0.0652	4.67
C-peptide (log) % change		1.11 (−1.42, 3.71)	0.3911	0.01
	Sex	1.00 (−1.57, 3.63)	0.4473	0.03
	+ Family history of type 2 diabetes	1.49 (−1.17, 4.21)	0.2735	0.14
	+ Age at enrolment	1.91 (−0.76, 4.64)	0.1619	0.59
	+ BMI	−3.51 (−5.92, −1.04)	0.0060	19.70
	+ Alcohol, smoking status, physical activity	−3.40 (−5.79, −0.94)	0.0073	20.81
hsCRP (log) % change		−3.67 (−8.67, 1.61)	0.1699	0.03
	Sex	0.53 (−4.68, 6.03)	0.8451	2.09
	+ Family history of type 2 diabetes	4.70 (−0.77, 10.47)	0.0934	3.52
	+ Age at enrolment	5.49 (−0.04, 11.31)	0.0516	3.76
	+ BMI	−5.03 (−9.79, −0.03)	0.0487	16.74
	+ Alcohol, smoking status, physical activity	−5.09 (−9.82, −0.11)	0.0454	18.75
HOMA2 insulin sensitivity (log) % change		−0.33 (−2.85, 2.26)	0.8018	0.00

	Sex	−0.13 (−2.71, 2.51)	0.9219	0.03
	+ Family history of type 2 diabetes	−1.04 (−3.65, 1.64)	0.4428	0.40
	+ Age at enrolment	−1.35 (−3.94, 1.31)	0.3146	0.68
	+ BMI	4.37 (1.77, 7.04)	0.0010	19.70
	+ Alcohol, smoking status, physical activity	4.19 (1.61, 6.84)	0.0015	21.00
HOMA2 beta (log) % change		1.83 (−0.59, 4.30)	0.1386	0.04
	Sex	2.76 (0.32, 5.26)	0.0266	0.64
	+ Family history of type 2 diabetes	1.58 (−0.87, 4.08)	0.2068	1.38
	+ Age at enrolment	1.92 (−0.55, 4.45)	0.1270	1.84
	+ BMI	−1.57 (−3.86, 0.78)	0.1881	10.89
	+ Alcohol, smoking status, physical activity	−1.64 (−3.93, 0.71)	0.1695	11.47

Legend: Stepwise adjustment for linear regression models. Coefficient = unit increase per 1000 g increase in birthweight. For logarithmic transformation, the coefficient represents percentage increase per increase in birthweight. Abbreviations: BMI = body mass index, BP = blood pressure, HDL = high-density lipoprotein, LDL = low-density lipoprotein, hsCRP = high-sensitivity C-reactive protein, HOMA2 = Homeostasis Model Assessment 2, log = natural logarithm.

ESM Table 7: Associations of birthweight with further adjustment for polygenic risk scores

		**Original**	**+ PRS type 2 diabetes and PRS BW**
Outcome	**Exposure**	**aPR (95% CI)**	**aPR (95% CI)**
	Age at diagnosis (years)		
<45	<3000g	1.28 (1.10, 1.48)	1.38 (1.08, 1.76)
<45	>3700g	0.69 (0.58, 0.83)	0.74 (0.54, 1.01)
45–55	<3000g	1.32 (1.20, 1.46)	1.35 (1.15, 1.59)
45–55	>3700g	0.87 (0.77, 0.97)	0.79 (0.65, 0.97)
55–65	<3000g	0.94 (0.87, 1.02)	0.99 (0.87, 1.13)
55–65	>3700g	0.93 (0.85, 1.01)	1.01 (0.88, 1.15)
65–75	<3000g	0.75 (0.67, 0.84)	0.66 (0.55, 0.80)
65–75	>3700g	1.24 (1.13, 1.36)	1.13 (0.97, 1.32)
≥75	<3000g	0.70 (0.53, 0.93)	0.72 (0.46, 1.11)
≥75	>3700g	1.71 (1.37, 2.12)	1.79 (1.27, 2.53)
	Family history of type 2 diabetes (n)		
0	<3000g	1.07 (1.01, 1.14)	1.02 (0.93, 1.12)
0	>3700g	0.88 (0.83, 0.94)	0.83 (0.75, 0.92)
1	<3000g	0.99 (0.91, 1.08)	1.07 (0.93, 1.22)
1	>3700g	1.09 (1.00, 1.18)	1.11 (0.96, 1.27)
2	<3000g	0.96 (0.83, 1.10)	0.89 (0.69, 1.14)
2	>3700g	1.12 (0.97, 1.29)	1.30 (1.03, 1.65)
3+	<3000g	0.67 (0.52, 0.88)	0.74 (0.47, 1.17)
3+	>3700g	1.25 (0.99, 1.58)	1.29 (0.87, 1.93)
	BMI (kg/m2)		
<25	<3000g	1.12 (1.03, 1.22)	1.11 (0.97, 1.26)
<25	>3700g	0.90 (0.82, 0.98)	0.75 (0.64, 0.88)
25–30	<3000g	1.11 (1.01, 1.22)	1.17 (1.00, 1.37)
25–30	>3700g	0.84 (0.76, 0.93)	0.94 (0.80, 1.12)
30–35	<3000g	0.98 (0.88, 1.09)	1.03 (0.87, 1.23)
30–35	>3700g	1.12 (1.01, 1.24)	1.22 (1.04, 1.44)
35–40	<3000g	0.84 (0.71, 0.99)	0.64 (0.48, 0.85)
35–40	>3700g	1.26 (1.09, 1.46)	1.24 (0.96, 1.60)
≥40	<3000g	0.57 (0.45, 0.72)	0.58 (0.39, 0.87)
≥40	>3700g	1.28 (1.05, 1.56)	1.23 (0.89, 1.69)
	Waist circumference (male/female in cm)		
<94/80	<3000g	1.34 (1.14, 1.58)	1.37 (1.05, 1.78)
<94/80	>3700g	0.70 (0.57, 0.85)	0.72 (0.52, 1.00)

94–102/80–88	<3000g	1.21 (1.05, 1.40)	1.25 (1.01, 1.54)
94–102/80–88	>3700g	0.75 (0.65, 0.88)	0.81 (0.64, 1.03)
>102/88	<3000g	0.93 (0.90, 0.96)	0.91 (0.86, 0.97)
>102/88	>3700g	1.09 (1.06, 1.12)	1.08 (1.02, 1.13)
	Systolic blood pressure (mmHg)		
<125	<3000g	0.97 (0.87, 1.07)	0.98 (0.83, 1.16)
<125	>3700g	1.12 (1.01, 1.23)	1.25 (1.08, 1.45)
125–134	<3000g	0.97 (0.88, 1.08)	0.94 (0.80, 1.10)
125–134	>3700g	0.95 (0.86, 1.05)	0.87 (0.74, 1.01)
135–154	<3000g	1.02 (0.92, 1.12)	1.00 (0.85, 1.18)
135–154	>3700g	0.90 (0.81, 1.00)	0.83 (0.70, 0.98)
≥155	<3000g	1.28 (1.01, 1.61)	1.37 (0.95, 1.96)
≥155	>3700g	1.19 (0.93, 1.54)	1.33 (0.95, 1.87)
	Antihypertensive drug usage		
Yes	<3000g	1.04 (1.00, 1.08)	1.04 (0.98, 1.10)
Yes	>3700g	0.98 (0.95, 1.02)	1.01 (0.95, 1.07)
0	<3000g	0.91 (0.83, 1.00)	0.91 (0.79, 1.06)
0	>3700g	1.04 (0.95, 1.14)	0.96 (0.82, 1.12)
1	<3000g	1.12 (1.01, 1.25)	0.98 (0.82, 1.17)
1	>3700g	1.02 (0.91, 1.14)	0.90 (0.74, 1.09)
2	<3000g	0.92 (0.83, 1.02)	1.00 (0.84, 1.18)
2	>3700g	0.94 (0.85, 1.04)	0.95 (0.79, 1.13)
3+	<3000g	1.09 (0.99, 1.20)	1.13 (0.96, 1.31)
3+	>3700g	0.99 (0.90, 1.09)	1.15 (1.00, 1.34)
	Glucose-lowering drug usage		
None	<3000g	0.98 (0.85, 1.12)	1.22 (0.99, 1.50)
None	>3700g	1.01 (0.88, 1.16)	0.96 (0.77, 1.20)
Oral	<3000g	0.99 (0.96, 1.02)	0.93 (0.88, 0.99)
Oral	>3700g	0.99 (0.96, 1.02)	0.99 (0.94, 1.05)
Insulin + oral or insulin only	<3000g	1.17 (0.94, 1.45)	1.28 (0.92, 1.78)
Insulin + oral or insulin only	>3700g	1.05 (0.84, 1.32)	1.20 (0.84, 1.72)
0	<3000g	0.98 (0.85, 1.12)	1.23 (0.99, 1.51)
0	>3700g	1.01 (0.89, 1.16)	0.96 (0.77, 1.20)
1	<3000g	0.96 (0.92, 1.01)	0.90 (0.83, 0.97)
1	>3700g	1.01 (0.96, 1.05)	1.03 (0.96, 1.10)
2	<3000g	1.07 (0.94, 1.22)	1.03 (0.82, 1.28)
2	>3700g	0.93 (0.81, 1.07)	0.89 (0.69, 1.13)

3+	<3000g	1.33 (1.06, 1.65)	1.52 (1.06, 2.17)
3+	>3700g	1.11 (0.87, 1.40)	1.09 (0.72, 1.65)
	Charlson Comorbidity Index score		
0	<3000g	0.97 (0.94, 1.01)	0.97 (0.91, 1.03)
0	>3700g	1.01 (0.97, 1.05)	1.00 (0.93, 1.07)
1–2	<3000g	1.01 (0.91, 1.11)	1.01 (0.86, 1.17)
1–2	>3700g	0.96 (0.87, 1.06)	0.91 (0.78, 1.06)
3+	<3000g	1.36 (1.07, 1.73)	1.41 (0.96, 2.07)
3+	>3700g	1.04 (0.82, 1.32)	1.43 (0.98, 2.09)
	Macrovascular complications		
Yes	<3000g	1.08 (0.95, 1.22)	0.99 (0.81, 1.21)
Yes	>3700g	0.92 (0.82, 1.04)	0.98 (0.82, 1.18)
	Microvascular complications		
Yes	<3000g	0.79 (0.66, 0.94)	0.70 (0.52, 0.94)
Yes	>3700g	0.96 (0.82, 1.14)	1.07 (0.82, 1.39)
	Diabetes-associated eye disease		
Yes	<3000g	0.97 (0.81, 1.17)	0.87 (0.64, 1.17)
Yes	>3700g	0.84 (0.70, 1.01)	0.90 (0.67, 1.20)
	Diabetes-associated neurological disease		
Yes	<3000g	0.74 (0.61, 0.90)	0.66 (0.48, 0.90)
Yes	>3700g	0.97 (0.81, 1.15)	0.98 (0.73, 1.30)
	Diabetes-associated kidney disease		
Yes	<3000g	1.18 (0.84, 1.65)	0.88 (0.49, 1.61)
Yes	>3700g	0.87 (0.62, 1.24)	1.47 (0.89, 2.45)

Legend: Prevalence ratios of birthweight and main outcomes adjusting for 1: base model (sex, age at enrolment and family history of type 2 diabetes); and 2: further adjusting for polygenic risk scores of type 2 diabetes and birthweight. Abbreviations: PRS = polygenic risk scores, BW = birthweight, aPR = adjusted prevalence ratio, 95% CI = 95% confidence interval, BMI = body mass index.

ESM Table 8 – Linear regression of polygenic risk scores and birthweight

Linear regression: increase in grams per 1 SD of PRS

Outcome	Exposure	Coefficient	95% CI	P-value	R^2
Birthweight	PRS type 2 diabetes	−16.83	−38.19, 4.52	0.1227	0.054%
Birthweight	PRS birthweight	127.30	106.51, 148.08	<0.0001	9.5%

Legend: Linear regression models using polygenic risk scores for type 2 diabetes or birthweight with birthweight. Coefficient = grams increase per one standard deviation increase in polygenic risk scores. Models were calculated using scaled PRS centering around the mean. Abbreviations: PRS = polygenic risk scores, CI = confidence intervals, SD = standard deviation.

ESM Table 9: Further adjustment of born-at-term-status.

		Base model	+ Born-at-term
Outcome	**Exposure**	**aPR**	**aPR**
	Age at Diagnosis (years)		
<45	<3000g	1.28 (1.10, 1.48)	1.22 (1.03, 1.45)
<45	>3700g	0.69 (0.58, 0.83)	0.69 (0.58, 0.83)
45–55	<3000g	1.32 (1.20, 1.46)	1.32 (1.19, 1.48)
45–55	>3700g	0.87 (0.77, 0.97)	0.87 (0.77, 0.97)
55–65	<3000g	0.94 (0.87, 1.02)	0.92 (0.84, 1.02)
55–65	>3700g	0.93 (0.85, 1.01)	0.93 (0.86, 1.01)
65–75	<3000g	0.75 (0.67, 0.84)	0.78 (0.69, 0.89)
65–75	>3700g	1.24 (1.13, 1.36)	1.24 (1.13, 1.36)
≥75	<3000g	0.70 (0.53, 0.93)	0.80 (0.58, 1.09)
≥75	>3700g	1.71 (1.37, 2.12)	1.70 (1.37, 2.11)
	Family History of type 2 diabetes (n)		
0	<3000g	1.07 (1.01, 1.14)	1.08 (1.01, 1.15)
0	>3700g	0.88 (0.83, 0.94)	0.88 (0.83, 0.94)
1	<3000g	0.99 (0.91, 1.08)	1.02 (0.92, 1.12)
1	>3700g	1.09 (1.00, 1.18)	1.09 (1.00, 1.18)
2	<3000g	0.96 (0.83, 1.10)	0.92 (0.79, 1.08)
2	>3700g	1.12 (0.97, 1.29)	1.12 (0.97, 1.29)
3+	<3000g	0.67 (0.52, 0.88)	0.59 (0.43, 0.82)
3+	>3700g	1.25 (0.99, 1.58)	1.26 (0.99, 1.59)
	BMI (kg/m2)		
<25	<3000g	1.12 (1.03, 1.22)	1.14 (1.04, 1.25)
<25	>3700g	0.90 (0.82, 0.98)	0.90 (0.82, 0.98)
25–30	<3000g	1.11 (1.01, 1.22)	1.11 (1.00, 1.24)
25–30	>3700g	0.84 (0.76, 0.93)	0.84 (0.76, 0.93)
30–35	<3000g	0.98 (0.88, 1.09)	0.97 (0.86, 1.10)
30–35	>3700g	1.12 (1.01, 1.24)	1.12 (1.01, 1.24)
35–40	<3000g	0.84 (0.71, 0.99)	0.82 (0.68, 0.99)
35–40	>3700g	1.26 (1.09, 1.46)	1.28 (1.10, 1.48)
≥40	<3000g	0.57 (0.45, 0.72)	0.53 (0.40, 0.71)
≥40	>3700g	1.28 (1.05, 1.56)	1.25 (1.03, 1.53)
	Waist circumference (male/female in cm)		
<94/80	<3000g	1.34 (1.14, 1.58)	1.38 (1.14, 1.65)
<94/80	>3700g	0.70 (0.57, 0.85)	0.70 (0.57, 0.85)

94–102/80–88	<3000g	1.21 (1.05, 1.40)	1.20 (1.02, 1.41)
94–102/80–88	>3700g	0.75 (0.65, 0.88)	0.75 (0.65, 0.88)
≥102/88	<3000g	0.93 (0.90, 0.96)	0.93 (0.89, 0.96)
≥102/88	>3700g	1.09 (1.06, 1.12)	1.09 (1.06, 1.12)
Systolic blood pressure (mmHg)			
<125	<3000g	0.96 (0.87, 1.07)	0.94 (0.84, 1.06)
<125	>3700g	1.10 (1.00, 1.21)	1.10 (1.00, 1.21)
125–134	<3000g	0.97 (0.88, 1.07)	0.97 (0.87, 1.08)
125–134	>3700g	0.96 (0.86, 1.06)	0.96 (0.86, 1.06)
135–154	<3000g	1.02 (0.92, 1.13)	1.03 (0.91, 1.16)
135–154	>3700g	0.91 (0.82, 1.01)	0.91 (0.82, 1.01)
≥155	<3000g	1.26 (0.99, 1.59)	1.31 (1.01, 1.71)
≥155	>3700g	1.18 (0.92, 1.52)	1.18 (0.92, 1.52)
Hypertension			
Yes	<3000g	1.02 (1.00, 1.05)	1.02 (1.00, 1.05)
Yes	>3700g	1.00 (0.97, 1.02)	1.00 (0.97, 1.02)
Antihypertensive drug usage			
Yes	<3000g	1.04 (1.00, 1.08)	1.02 (0.98, 1.07)
Yes	>3700g	0.98 (0.95, 1.02)	0.98 (0.95, 1.02)
0	<3000g	0.91 (0.83, 1.00)	0.94 (0.85, 1.04)
0	>3700g	1.04 (0.95, 1.14)	1.04 (0.94, 1.13)
1	<3000g	1.12 (1.01, 1.25)	1.10 (0.97, 1.25)
1	>3700g	1.02 (0.91, 1.14)	1.02 (0.92, 1.14)
2	<3000g	0.92 (0.83, 1.02)	0.93 (0.83, 1.05)
2	>3700g	0.94 (0.85, 1.04)	0.94 (0.85, 1.04)
3+	<3000g	1.09 (0.99, 1.20)	1.05 (0.94, 1.17)
3+	>3700g	0.99 (0.90, 1.09)	0.99 (0.90, 1.09)
Glucose-lowering drug usage			
None	<3000g	0.98 (0.85, 1.12)	0.98 (0.84, 1.15)
None	>3700g	1.01 (0.88, 1.16)	1.01 (0.88, 1.16)
Oral	<3000g	0.99 (0.96, 1.02)	0.99 (0.96, 1.03)
Oral	>3700g	0.99 (0.96, 1.02)	0.99 (0.96, 1.02)
Insulin + oral or insulin only	<3000g	1.17 (0.94, 1.45)	1.15 (0.90, 1.47)
Insulin + oral or insulin only	>3700g	1.05 (0.84, 1.32)	1.05 (0.84, 1.32)
0	<3000g	0.98 (0.85, 1.12)	0.98 (0.84, 1.15)
0	>3700g	1.01 (0.89, 1.16)	1.01 (0.89, 1.16)
1	<3000g	0.96 (0.92, 1.01)	0.98 (0.93, 1.03)

1	>3700g	1.01 (0.96, 1.05)	1.01 (0.96, 1.05)
2	<3000g	1.07 (0.94, 1.22)	1.03 (0.88, 1.21)
2	>3700g	0.93 (0.81, 1.07)	0.93 (0.81, 1.07)
3+	<3000g	1.33 (1.06, 1.65)	1.24 (0.96, 1.60)
3+	>3700g	1.11 (0.87, 1.40)	1.11 (0.87, 1.40)
	Charlson Comorbidity Index		
0	<3000g	0.97 (0.94, 1.01)	0.98 (0.94, 1.03)
0	>3700g	1.01 (0.97, 1.05)	1.01 (0.97, 1.05)
1–2	<3000g	1.01 (0.91, 1.11)	1.00 (0.89, 1.12)
1–2	>3700g	0.96 (0.87, 1.06)	0.95 (0.87, 1.05)
3+	<3000g	1.36 (1.07, 1.73)	1.27 (0.97, 1.68)
3+	>3700g	1.04 (0.82, 1.32)	1.04 (0.82, 1.32)
	Macrovascular complications		
Yes	<3000g	1.08 (0.95, 1.22)	1.11 (0.97, 1.28)
Yes	>3700g	0.92 (0.82, 1.04)	0.92 (0.82, 1.04)
	Microvascular complications		
Yes	<3000g	0.79 (0.66, 0.94)	0.77 (0.63, 0.95)
Yes	>3700g	0.96 (0.82, 1.14)	0.96 (0.82, 1.14)
	Diabetes-associated eye disease		
Yes	<3000g	0.97 (0.81, 1.17)	1.04 (0.85, 1.28)
Yes	>3700g	0.84 (0.70, 1.01)	0.84 (0.70, 1.01)
	Diabetes-associated kidney disease		
Yes	<3000g	1.18 (0.84, 1.65)	1.17 (0.81, 1.69)
Yes	>3700g	0.87 (0.62, 1.24)	0.87 (0.62, 1.24)
	Diabetes-associated neurological disease		
Yes	<3000g	0.74 (0.61, 0.90)	0.70 (0.56, 0.88)
Yes	>3700g	0.97 (0.81, 1.15)	0.97 (0.81, 1.16)

Legend: Prevalence ratios, base model: sex, age at enrolment, and family history of type 2 diabetes. Abbreviations: BMI = body mass index, BP = blood pressure, HOMA2 = Homeostasis Model Assessment 2, aPR = adjusted prevalence ratio.

ESM Table 10: Prevalence ratios of main analysis birthweights and conventional birthweights

		Main analysis BW	Conventional BW
Outcome	**Exposure**	**aPR**	**aPR**
	Age at diagnosis (years)		
<45	<3000g / <2500g	1.28 (1.10, 1.48)	1.58 (1.27, 1.97)
<45	>3700g / >4500g	0.69 (0.58, 0.83)	0.66 (0.34, 1.28)
45–55	<3000g / <2500g	1.32 (1.20, 1.46)	1.26 (1.07, 1.48)
45–55	>3700g / >4500g	0.87 (0.77, 0.97)	0.79 (0.53, 1.18)
55–65	<3000g / <2500g	0.94 (0.87, 1.02)	1.05 (0.92, 1.20)
55–65	>3700g / >4500g	0.93 (0.85, 1.01)	0.94 (0.70, 1.25)
65–75	<3000g / <2500g	0.75 (0.67, 0.84)	0.58 (0.46, 0.73)
65–75	>3700g / >4500g	1.24 (1.13, 1.36)	1.30 (0.98, 1.74)
≥75	<3000g / <2500g	0.70 (0.53, 0.93)	0.48 (0.26, 0.86)
≥75	>3700g / >4500g	1.71 (1.37, 2.12)	1.76 (0.94, 3.31)
	Family history of type 2 diabetes (n)		
0	<3000g / <2500g	1.07 (1.01, 1.14)	1.07 (0.96, 1.18)
0	>3700g / >4500g	0.88 (0.83, 0.94)	0.76 (0.59, 0.97)
1	<3000g / <2500g	0.99 (0.91, 1.08)	0.95 (0.82, 1.10)
1	>3700g / >4500g	1.09 (1.00, 1.18)	1.00 (0.75, 1.34)
2	<3000g / <2500g	0.96 (0.83, 1.10)	0.93 (0.73, 1.18)
2	>3700g / >4500g	1.12 (0.97, 1.29)	1.51 (1.03, 2.21)
3+	<3000g / <2500g	0.67 (0.52, 0.88)	0.98 (0.66, 1.46)
3+	>3700g / >4500g	1.25 (0.99, 1.58)	1.98 (1.07, 3.67)
	BMI (kg/m2)		
<25	<3000g / <2500g	1.12 (1.03, 1.22)	1.17 (1.03, 1.34)
<25	>3700g / >4500g	0.90 (0.82, 0.98)	0.85 (0.62, 1.17)
25–30	<3000g / <2500g	1.11 (1.01, 1.22)	1.26 (1.07, 1.47)
25–30	>3700g / >4500g	0.84 (0.76, 0.93)	0.82 (0.56, 1.18)
30–35	<3000g / <2500g	0.98 (0.88, 1.09)	0.82 (0.67, 1.01)
30–35	>3700g / >4500g	1.12 (1.01, 1.24)	1.09 (0.78, 1.52)
35–40	<3000g / <2500g	0.84 (0.71, 0.99)	0.73 (0.54, 0.98)
35–40	>3700g / >4500g	1.26 (1.09, 1.46)	1.68 (1.12, 2.52)
≥40	<3000g / <2500g	0.57 (0.45, 0.72)	0.59 (0.38, 0.90)
≥40	>3700g / >4500g	1.28 (1.05, 1.56)	1.01 (0.47, 2.16)
	Waist circumference (male/female in cm)		
<94/80	<3000g / <2500g	1.34 (1.14, 1.58)	1.33 (1.01, 1.75)
<94/80	>3700g / >4500g	0.70 (0.57, 0.85)	0.58 (0.27, 1.27)

94–102/80–88	<3000g / <2500g	1.21 (1.05, 1.40)	1.31 (1.03, 1.66)
94–102/80–88	>3700g / >4500g	0.75 (0.65, 0.88)	0.80 (0.46, 1.37)
≥102/88	<3000g / <2500g	0.93 (0.90, 0.96)	0.92 (0.87, 0.98)
≥102/88	>3700g / >4500g	1.09 (1.06, 1.12)	1.09 (1.00, 1.20)
	Systolic blood pressure (mmHg)		
<125	<3000g / <2500g	0.96 (0.87, 1.07)	1.01 (0.86, 1.19)
<125	>3700g / >4500g	1.10 (1.00, 1.21)	1.03 (0.74, 1.42)
125–134	<3000g / <2500g	0.97 (0.88, 1.07)	0.96 (0.82, 1.13)
125–134	>3700g / >4500g	0.96 (0.86, 1.06)	0.82 (0.57, 1.17)
135–154	<3000g / <2500g	1.02 (0.92, 1.13)	0.98 (0.81, 1.18)
135–154	>3700g / >4500g	0.91 (0.82, 1.01)	1.16 (0.85, 1.58)
≥155	<3000g / <2500g	1.26 (0.99, 1.59)	1.21 (0.80, 1.83)
≥155	>3700g / >4500g	1.18 (0.92, 1.52)	1.08 (0.52, 2.25)
	Hypertension		
Yes	<3000g / <2500g	1.02 (1.00, 1.05)	1.03 (0.99, 1.07)
Yes	>3700g / >4500g	1.00 (0.97, 1.02)	0.92 (0.84, 1.01)
	Antihypertensive drug usage		
Yes	<3000g / <2500g	1.04 (1.00, 1.08)	1.08 (1.02, 1.15)
Yes	>3700g / >4500g	0.98 (0.95, 1.02)	0.95 (0.84, 1.07)
0	<3000g / <2500g	0.91 (0.83, 1.00)	1.05 (0.88, 1.26)
0	>3700g / >4500g	1.04 (0.95, 1.14)	1.09 (0.76, 1.55)
1	<3000g / <2500g	1.12 (1.01, 1.25)	0.98 (0.81, 1.17)
1	>3700g / >4500g	1.02 (0.91, 1.14)	0.91 (0.64, 1.31)
2	<3000g / <2500g	0.92 (0.83, 1.02)	1.22 (1.04, 1.43)
2	>3700g / >4500g	0.94 (0.85, 1.04)	0.89 (0.62, 1.26)
3+	<3000g / <2500g	1.09 (0.99, 1.20)	0.82 (0.70, 0.97)
3+	>3700g / >4500g	0.99 (0.90, 1.09)	1.15 (0.86, 1.53)
	Glucose-lowering drug usage		
None	<3000g / <2500g	0.98 (0.85, 1.12)	0.82 (0.62, 1.07)
None	>3700g / >4500g	1.01 (0.88, 1.16)	1.04 (0.67, 1.61)
Oral	<3000g / <2500g	0.99 (0.96, 1.02)	1.02 (0.97, 1.08)
Oral	>3700g / >4500g	0.99 (0.96, 1.02)	0.99 (0.88, 1.10)
Insulin + oral or insulin only	<3000g / <2500g	1.17 (0.94, 1.45)	1.12 (0.79, 1.58)
Insulin + oral or insulin only	>3700g / >4500g	1.05 (0.84, 1.32)	1.06 (0.49, 2.31)
0	<3000g / <2500g	0.98 (0.85, 1.12)	0.82 (0.63, 1.07)
0	>3700g / >4500g	1.01 (0.89, 1.16)	1.06 (0.68, 1.64)
1	<3000g / <2500g	0.96 (0.92, 1.01)	0.99 (0.92, 1.07)

1	>3700g / >4500g	1.01 (0.96, 1.05)	1.05 (0.91, 1.20)
2	<3000g / <2500g	1.07 (0.94, 1.22)	1.05 (0.84, 1.31)
2	>3700g / >4500g	0.93 (0.81, 1.07)	0.76 (0.44, 1.32)
3+	<3000g / <2500g	1.33 (1.06, 1.65)	1.40 (1.01, 1.95)
3+	>3700g / >4500g	1.11 (0.87, 1.40)	1.13 (0.52, 2.44)
	Charlson Comorbidity Index		
0	<3000g / <2500g	0.97 (0.94, 1.01)	0.92 (0.86, 0.99)
0	>3700g / >4500g	1.01 (0.97, 1.05)	0.95 (0.82, 1.11)
1–2	<3000g / <2500g	1.01 (0.91, 1.11)	1.11 (0.94, 1.30)
1–2	>3700g / >4500g	0.96 (0.87, 1.06)	0.95 (0.68, 1.32)
3+	<3000g / <2500g	1.36 (1.07, 1.73)	1.72 (1.20, 2.45)
3+	>3700g / >4500g	1.04 (0.82, 1.32)	1.77 (0.98, 3.20)
	Macrovascular complications		
Yes	<3000g / <2500g	1.08 (0.95, 1.22)	1.16 (0.94, 1.44)
Yes	>3700g / >4500g	0.92 (0.82, 1.04)	0.69 (0.43, 1.11)
	Microvascular complications		
Yes	<3000g / <2500g	0.79 (0.66, 0.94)	0.86 (0.63, 1.17)
Yes	>3700g / >4500g	0.96 (0.82, 1.14)	1.09 (0.64, 1.87)
	Diabetes-associated eye disease		
Yes	<3000g / <2500g	0.97 (0.81, 1.17)	1.07 (0.78, 1.47)
Yes	>3700g / >4500g	0.84 (0.70, 1.01)	1.04 (0.58, 1.87)
	Diabetes-associated kidney disease		
Yes	<3000g / <2500g	1.18 (0.84, 1.65)	0.85 (0.44, 1.65)
Yes	>3700g / >4500g	0.87 (0.62, 1.24)	1.32 (0.49, 3.50)
	Diabetes-associated neurological disease		
Yes	<3000g / <2500g	0.74 (0.61, 0.90)	0.88 (0.64, 1.22)
Yes	>3700g / >4500g	0.97 (0.81, 1.15)	1.16 (0.66, 2.03)

Legend: Prevalence ratios according to main analysis using birthweight (BW <3000 g and BW >3700 g) and conventional birthweights (low BW <2500 g and high BW >4500 g); adjusted for sex, age at enrolment, and family history of type 2 diabetes. Abbreviations: BMI = body mass index, BP = blood pressure, HOMA2 = Homeostasis Model Assessment 2, aPR = adjusted prevalence ratio.

ESM Table 11: Alternative sex and born-at-term birthweight categories.
The following logic has been used to create the alternative sex and born-at-term quartile BW categories:
Male and born-at-term: <25% = 3200 g and >75% = 3800 g
Male and not born-at-term: <25% = 2275 g and >75% = 2800 g
Female and born-at-term: <25% = 3100 g and >75% = 3685 g
Female and not born-at-term: <25% = 2250 g and >75% = 2800 g

		Main analysis BW	Alternative sex and born-at-term BW
Outcome	**Exposure**	**aPR**	**aPR**
	Age at diagnosis (years)		
<45	<3000g / <25%	1.28 (1.10, 1.48)	1.29 (1.11, 1.50)
<45	>3700g / >75%	0.69 (0.58, 0.83)	0.77 (0.64, 0.92)
45–55	<3000g / <25%	1.32 (1.20, 1.46)	1.28 (1.16, 1.42)
45–55	>3700g / >75%	0.87 (0.77, 0.97)	0.90 (0.80, 1.01)
55–65	<3000g / <25%	0.94 (0.87, 1.02)	0.94 (0.87, 1.02)
55–65	>3700g / >75%	0.93 (0.85, 1.01)	0.91 (0.84, 0.99)
65–75	<3000g / <25%	0.75 (0.67, 0.84)	0.77 (0.69, 0.86)
65–75	>3700g / >75%	1.24 (1.13, 1.36)	1.20 (1.09, 1.31)
≥75	<3000g / <25%	0.70 (0.53, 0.93)	0.73 (0.55, 0.97)
≥75	>3700g / >75%	1.71 (1.37, 2.12)	1.66 (1.33, 2.06)
	Family history of type 2 diabetes (n)		
0	<3000g / <25%	1.07 (1.01, 1.14)	1.04 (0.98, 1.10)
0	>3700g / >75%	0.88 (0.83, 0.94)	0.90 (0.84, 0.95)
1	<3000g / <25%	0.99 (0.91, 1.08)	1.01 (0.93, 1.10)
1	>3700g / >75%	1.09 (1.00, 1.18)	1.07 (0.98, 1.16)
2	<3000g / <25%	0.96 (0.83, 1.10)	0.98 (0.85, 1.13)
2	>3700g / >75%	1.12 (0.97, 1.29)	1.13 (0.98, 1.29)
3+	<3000g / <25%	0.67 (0.52, 0.88)	0.70 (0.53, 0.92)
3+	>3700g / >75%	1.25 (0.99, 1.58)	1.21 (0.96, 1.53)
	BMI (kg/m2)		
<25	<3000g / <25%	1.12 (1.03, 1.22)	1.12 (1.03, 1.21)
<25	>3700g / >75%	0.90 (0.82, 0.98)	0.88 (0.81, 0.97)
25–30	<3000g / <25%	1.11 (1.01, 1.22)	1.11 (1.01, 1.22)
25–30	>3700g / >75%	0.84 (0.76, 0.93)	0.87 (0.78, 0.96)
30–35	<3000g / <25%	0.98 (0.88, 1.09)	0.99 (0.89, 1.10)
30–35	>3700g / >75%	1.12 (1.01, 1.24)	1.09 (0.98, 1.21)
35–40	<3000g / <25%	0.84 (0.71, 0.99)	0.80 (0.68, 0.95)
35–40	>3700g / >75%	1.26 (1.09, 1.46)	1.25 (1.08, 1.44)
≥40	<3000g / <25%	0.57 (0.45, 0.72)	0.60 (0.47, 0.77)
≥40	>3700g / >75%	1.28 (1.05, 1.56)	1.35 (1.12, 1.64)
	Waist circumference (male/female in cm)		
<94/80	<3000g / <25%	1.34 (1.14, 1.58)	1.35 (1.15, 1.59)
<94/80	>3700g / >75%	0.70 (0.57, 0.85)	0.66 (0.54, 0.81)
94–102/80–88	<3000g / <25%	1.21 (1.05, 1.40)	1.18 (1.02, 1.35)

94–102/80–88	>3700g / >75%	0.75 (0.65, 0.88)	0.81 (0.70, 0.95)
≥102/88	<3000g / <25%	0.93 (0.90, 0.96)	0.93 (0.90, 0.96)
≥102/88	>3700g / >75%	1.09 (1.06, 1.12)	1.08 (1.05, 1.11)
	Systolic blood pressure (mmHg)		
<125	<3000g / <25%	0.96 (0.87, 1.07)	0.97 (0.88, 1.08)
<125	>3700g / >75%	1.10 (1.00, 1.21)	1.06 (0.96, 1.18)
125–134	<3000g / <25%	0.97 (0.88, 1.07)	0.98 (0.89, 1.08)
125–134	>3700g / >75%	0.96 (0.86, 1.06)	0.97 (0.89, 1.06)
135–154	<3000g / <25%	1.02 (0.92, 1.13)	1.00 (0.90, 1.12)
135–154	>3700g / >75%	0.91 (0.82, 1.01)	0.93 (0.83, 1.04)
≥155	<3000g / <25%	1.26 (0.99, 1.59)	1.22 (0.96, 1.55)
≥155	>3700g / >75%	1.18 (0.92, 1.52)	1.19 (0.94, 1.51)
	Hypertension		
Yes	<3000g / <25%	1.02 (1.00, 1.05)	1.03 (1.00, 1.05)
Yes	>3700g / >75%	1.00 (0.97, 1.02)	1.00 (0.98, 1.03)
	C-peptide (pmol/l)		
<850	<3000g / <25%	1.02 (0.91, 1.13)	1.05 (0.93, 1.18)
<850	>3700g / >75%	0.96 (0.86, 1.07)	0.95 (0.84, 1.06)
850–1550	<3000g / <25%	1.01 (0.94, 1.08)	1.00 (0.93, 1.07)
850–1550	>3700g / >75%	0.95 (0.89, 1.02)	0.95 (0.89, 1.02)
≥1550	<3000g / <25%	0.98 (0.87, 1.10)	0.95 (0.85, 1.07)
≥1550	>3700g / >75%	1.14 (1.02, 1.27)	1.15 (1.03, 1.29)
	HOMA2-beta		
<70	<3000g / <25%	1.03 (0.94, 1.14)	1.06 (0.95, 1.18)
<70	>3700g / >75%	0.97 (0.87, 1.07)	0.93 (0.82, 1.05)
70–121	<3000g / <25%	1.01 (0.93, 1.09)	1.02 (0.95, 1.09)
70–121	>3700g / >75%	0.95 (0.88, 1.02)	0.96 (0.89, 1.03)
>121	<3000g / <25%	0.95 (0.84, 1.07)	0.90 (0.79, 1.02)
>121	>3700g / >75%	1.15 (1.03, 1.28)	1.16 (1.04, 1.30)
	Antihypertensive drug usage		
Yes	<3000g / <25%	1.04 (1.00, 1.08)	1.03 (0.99, 1.06)
Yes	>3700g / >75%	0.98 (0.95, 1.02)	0.99 (0.96, 1.03)
0	<3000g / <25%	0.91 (0.83, 1.00)	0.94 (0.86, 1.02)
0	>3700g / >75%	1.04 (0.95, 1.14)	1.00 (0.91, 1.10)
1	<3000g / <25%	1.12 (1.01, 1.25)	1.07 (0.96, 1.19)
1	>3700g / >75%	1.02 (0.91, 1.14)	1.07 (0.96, 1.19)
2	<3000g / <25%	0.92 (0.83, 1.02)	0.98 (0.88, 1.08)
2	>3700g / >75%	0.94 (0.85, 1.04)	0.95 (0.86, 1.06)
3+	<3000g / <25%	1.09 (0.99, 1.20)	1.04 (0.94, 1.15)
3+	>3700g / >75%	0.99 (0.90, 1.09)	0.97 (0.88, 1.07)
	Glucose-lowering drug usage		
None	<3000g / <25%	0.98 (0.85, 1.12)	0.93 (0.81, 1.07)

None	>3700g / >75%	1.01 (0.88, 1.16)	0.95 (0.83, 1.09)
Oral	<3000g / <25%	0.99 (0.96, 1.02)	1.01 (0.97, 1.04)
Oral	>3700g / >75%	0.99 (0.96, 1.02)	1.00 (0.97, 1.04)
Insulin + Oral or Insulin only	<3000g / <25%	1.17 (0.94, 1.45)	1.10 (0.88, 1.36)
Insulin + Oral or Insulin only	>3700g / >75%	1.05 (0.84, 1.32)	1.04 (0.82, 1.30)
0	<3000g / <25%	0.98 (0.85, 1.12)	0.93 (0.81, 1.07)
0	>3700g / >75%	1.01 (0.89, 1.16)	0.96 (0.83, 1.10)
1	<3000g / <25%	0.96 (0.92, 1.01)	0.99 (0.95, 1.04)
1	>3700g / >75%	1.01 (0.96, 1.05)	1.02 (0.98, 1.07)
2	<3000g / <25%	1.07 (0.94, 1.22)	1.02 (0.89, 1.17)
2	>3700g / >75%	0.93 (0.81, 1.07)	0.94 (0.81, 1.08)
3+	<3000g / <25%	1.33 (1.06, 1.65)	1.18 (0.95, 1.48)
3+	>3700g / >75%	1.11 (0.87, 1.40)	1.09 (0.86, 1.38)
	Charlson Comorbidity Index score		
0	<3000g / <25%	0.97 (0.94, 1.01)	0.98 (0.95, 1.02)
0	>3700g / >75%	1.01 (0.97, 1.05)	1.02 (0.98, 1.06)
1–2	<3000g / <25%	1.01 (0.91, 1.11)	1.01 (0.91, 1.11)
1–2	>3700g / >75%	0.96 (0.87, 1.06)	0.94 (0.85, 1.04)
3+	<3000g / <25%	1.36 (1.07, 1.73)	1.17 (0.92, 1.50)
3+	>3700g / >75%	1.04 (0.82, 1.32)	1.00 (0.78, 1.27)
	Macrovascular complications		
Yes	<3000g / <25%	1.08 (0.95, 1.22)	1.09 (0.97, 1.23)
Yes	>3700g / >75%	0.92 (0.82, 1.04)	0.86 (0.76, 0.97)
	Microvascular complications		
Yes	<3000g / <25%	0.79 (0.66, 0.94)	0.85 (0.72, 1.02)
Yes	>3700g / >75%	0.96 (0.82, 1.14)	0.95 (0.81, 1.12)
	Diabetes-associated eye disease		
Yes	<3000g / <25%	0.97 (0.81, 1.17)	1.02 (0.84, 1.23)
Yes	>3700g / >75%	0.84 (0.70, 1.01)	0.88 (0.73, 1.05)
	Diabetes-associated kidney disease		
Yes	<3000g / <25%	1.18 (0.84, 1.65)	0.80 (0.56, 1.14)
Yes	>3700g / >75%	0.87 (0.62, 1.24)	0.65 (0.46, 0.94)
	Diabetes-associated neurological disease		
Yes	<3000g / <25%	0.74 (0.61, 0.90)	0.83 (0.69, 1.00)
Yes	>3700g / >75%	0.97 (0.81, 1.15)	0.99 (0.83, 1.18)

Legend: Prevalence ratios using main analysis birthweight categories: BW <3000 g and BW >3700 g, and alternative birthweight categories defined by sex and born-at-term specific quartiles <25% = <Q1 BW and >75% >Q3 BW. All models adjusted for sex, age at enrolment, and family history of type 2 diabetes. Abbreviations: BMI = body mass index, HOMA2 = Homeostasis Model Assessment 2, PR = prevalence ratio.

ESM Figures

ESM Fig. 1: Forest plot of lipids, glucose homeostasis, and subclinical inflammation.

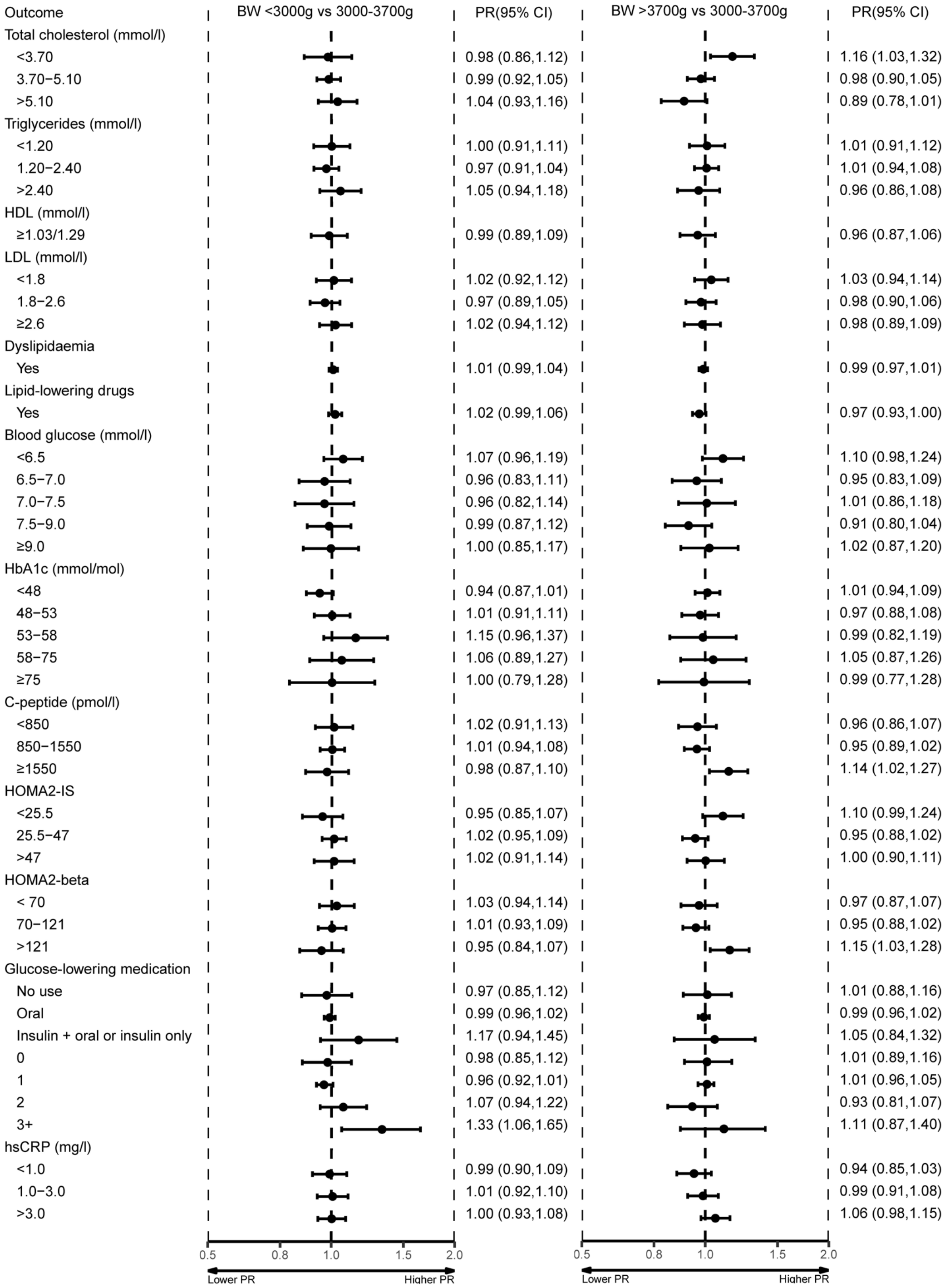

Legend: Adjusted prevalence ratios of measures of lipids, glucose homeostasis and subclinical inflammation according to birthweight. Adjusted for sex, age at enrolment, and family history of type 2 diabetes. Abbreviations: CI = confidence interval, HbA_{1c} = haemoglobin A_{1c}, HOMA2 = Homeostasis Model Assessment 2, IS = insulin sensitivity, hsCRP = high-sensitivity C-reactive protein, HDL = high-density lipoprotein, LDL = low-density lipoprotein, PR = prevalence ratio.

ESM Fig. 2: Forest plot of polygenic risk scores and birthweight

Outcome	Exposure	n	mean (sd)	PR (95% CI)
Birthweight (g)		2563	52.69 (0.11)	
<3000g	PRS T2D	641	52.69 (0.11)	1.02 (0.96,1.10)
3000−3700g	PRS T2D	1299	52.69 (0.11)	1.01 (0.97,1.05)
>3700g	PRS T2D	623	52.68 (0.1)	0.95 (0.89,1.02)
FH of type 2 diabetes		2563		
0	PRS T2D	1269	52.68 (0.11)	0.94 (0.90,0.98)
1	PRS T2D	829	52.69 (0.11)	1.01 (0.95,1.07)
2	PRS T2D	343	52.7 (0.1)	1.09 (0.98,1.20)
3+	PRS T2D	122	52.73 (0.11)	1.48 (1.24,1.75)
Birthweight (g)		2563	0.18 (0.13)	
<3000g	PRS BW	641	0.15 (0.12)	0.76 (0.70,0.82)
3000−3700g	PRS BW	1299	0.18 (0.13)	0.97 (0.93,1.07)
>3700g	PRS BW	623	0.23 (0.13)	1.42 (1.32,1.54)

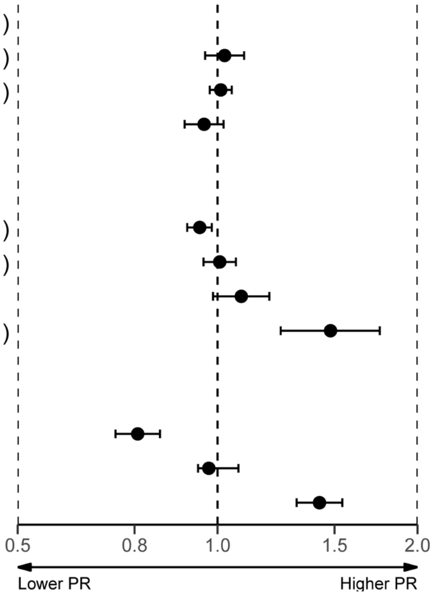

Legend: Forest plot of polygenic risk score sensitivity analysis. Polygenic risk scores were computed for risk of type 2 diabetes and birthweight. Scaled mean (sd) was scaled for centring around the mean and calculating standard deviations. Prevalence ratios were calculated by log-binomial and robust Poisson regression, using the scaled values. Abbreviations: g = grams, sd = standard deviations, T2D = type 2 diabetes, PRS = polygenic risk scores, PR = prevalence ratios, CI = confidence intervals, BW = birthweight; FH = family history.

Supplementary References:

[1] Bo A, Thomsen RW, Nielsen JS, et al. (2018) Early-onset type 2 diabetes: Age gradient in clinical and behavioural risk factors in 5115 persons with newly diagnosed type 2 diabetes-Results from the DD2 study. Diabetes Metab Res Rev 34(3). 10.1002/dmrr.2968
[2] Schneider HJ, Glaesmer H, Klotsche J, et al. (2007) Accuracy of anthropometric indicators of obesity to predict cardiovascular risk. J Clin Endocrinol Metab 92(2): 589-594. 10.1210/jc.2006-0254
[3] Association AD (2004) Dyslipidemia Management in Adults With Diabetes. Diabetes Care 27(suppl_1): s68-s71. 10.2337/diacare.27.2007.S68
[4] Haffner SM (2003) Management of dyslipidemia in adults with diabetes. Diabetes Care 26 Suppl 1: S83-86. 10.2337/diacare.26.2007.s83
[5] Olesen SS, Svane HML, Nicolaisen SK, et al. (2021) Clinical and biochemical characteristics of postpancreatitis diabetes mellitus: A cross-sectional study from the Danish nationwide DD2 cohort. J Diabetes 13(12): 960-974. 10.1111/1753-0407.13210
[6] Christensen DH, Knudsen ST, Gylfadottir SS, et al. (2020) Metabolic Factors, Lifestyle Habits, and Possible Polyneuropathy in Early Type 2 Diabetes: A Nationwide Study of 5,249 Patients in the Danish Centre for Strategic Research in Type 2 Diabetes (DD2) Cohort. Diabetes Care 43(6): 1266-1275. 10.2337/dc19-2277
[7] Hill NR, Levy JC, Matthews DR (2013) Expansion of the homeostasis model assessment of β-cell function and insulin resistance to enable clinical trial outcome modeling through the interactive adjustment of physiology and treatment effects: iHOMA2. Diabetes Care 36(8): 2324-2330. 10.2337/dc12-0607
[8] Nielsen AA, Christensen H, Lund ED, Christensen C, Brandslund I, Green A (2014) Diabetes mortality differs between registers due to various disease definitions. Dan Med J 61(5): A4840
[9] Svensson E, Berencsi K, Sander S, et al. (2016) Association of parental history of type 2 diabetes with age, lifestyle, anthropometric factors, and clinical severity at type 2 diabetes diagnosis: results from the DD2 study. Diabetes Metab Res Rev 32(3): 308-315. 10.1002/dmrr.2721
[10] Lynge E, Sandegaard JL, Rebolj M (2011) The Danish National Patient Register. Scand J Public Health 39(7 Suppl): 30-33. 10.1177/1403494811401482
[11] Charlson ME, Pompei P, Ales KL, MacKenzie CR (1987) A new method of classifying prognostic comorbidity in longitudinal studies: development and validation. J Chronic Dis 40(5): 373-383. 10.1016/0021-9681(87)90171-8
[12] Thygesen SK, Christiansen CF, Christensen S, Lash TL, Sørensen HT (2011) The predictive value of ICD-10 diagnostic coding used to assess Charlson comorbidity index conditions in the population-based Danish National Registry of Patients. BMC Med Res Methodol 11: 83. 10.1186/1471-2288-11-83
[13] Arkivskaber: Landetslægedistrikter, lægekredse, hospitaler og sygehuse. In:
[14] Archives DN Arkivskaber: Sundhedsstyrelsen. In:
[15] Pedersen ENKP (1998) På embeds vegne.
[16] Rigshospitalet GGRW (14/09-2018) Videnscenter for tidligt fødte børn
[17] Thestrup P (1991) Pund og Alen
[18] Stone CJ (1986) [Generalized Additive Models]: Comment. Statistical Science 1(3): 312-314
[19] Harrell FE (2016) Regression modeling strategies. Springer International Publishing, Cham, Switzerland
[20] van Buuren S, Groothuis-Oudshoorn K (2011) mice: Multivariate Imputation by Chained Equations in R. Journal of Statistical Software 45(3): 1 - 67. 10.18637/jss.v045.i03
[21] Khera AV, Chaffin M, Aragam KG, et al. (2018) Genome-wide polygenic scores for common diseases identify individuals with risk equivalent to monogenic mutations. Nature Genetics 50(9): 1219-1224. 10.1038/s41588-018-0183-z
[22] Privé F, Aschard H, Carmi S, et al. (2022) Portability of 245 polygenic scores when derived from the UK Biobank and applied to 9 ancestry groups from the same cohort. Am J Hum Genet 109(1): 12-23. 10.1016/j.ajhg.2021.11.008
[23] Stidsen JV, Henriksen JE, Olsen MH, et al. (2018) Pathophysiology-based phenotyping in type 2 diabetes: A clinical classification tool. Diabetes Metab Res Rev 34(5): e3005. 10.1002/dmrr.3005
[24] Ahlqvist E, Storm P, Käräjämäki A, et al. (2018) Novel subgroups of adult-onset diabetes and their association with outcomes: a data-driven cluster analysis of six variables. Lancet Diabetes Endocrinol 6(5): 361-369. 10.1016/s2213-8587(18)30051-2
[25] Christensen DH, Nicolaisen SK, Ahlqvist E, et al. (2022) Type 2 diabetes classification: a data-driven cluster study of the Danish Centre for Strategic Research in Type 2 Diabetes (DD2) cohort. BMJ Open Diabetes Research & Care 10(2): e002731. 10.1136/bmjdrc-2021-002731
